# Supplementary material for: Proteomic predictors of individualized nutrient-specific insulin secretion in health and disease
Source: Cell Metab. 2024 Jul 2;36(7):1619–1633.e5. doi: 10.1016/j.cmet.2024.06.001 (PMC11250105; doi:10.1016/j.cmet.2024.06.001)
Supplement: Document S1. Figures S1–S15 [file mmc1.pdf]

Cell Metabolism, Volume 36

## Supplemental information

# Proteomic predictors of individualized nutrient-specific insulin secretion in health and disease

**Jelena Kolic, WenQing Grace Sun, Haoning Howard Cen, Jessica D. Ewald, Jason C. Rogalski, Shugo Sasaki, Han Sun, Varsha Rajesh, Yi Han Xia, Renata Moravcova, Søs Skovsø, Aliya F. Spigelman, Jocelyn E. Manning Fox, James Lyon, Leanne Beet, Jianguo Xia, Francis C. Lynn, Anna L. Gloyn, Leonard J. Foster, Patrick E. MacDonald, and James D. Johnson**

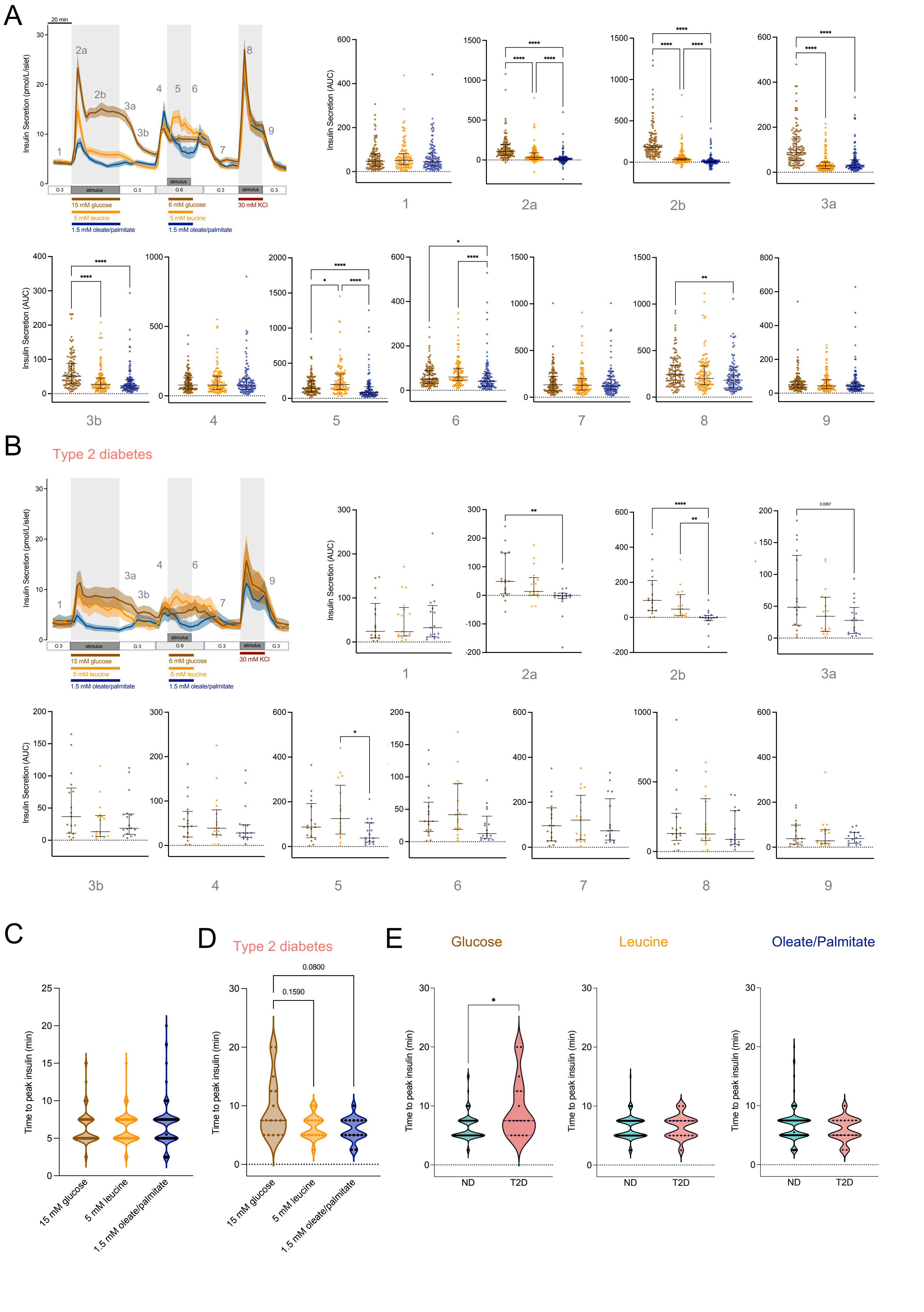
A
20 min
Insulin Secretion (pmol/L/islet)
1
2a
2b
3a
3b
4
5
6
7
8
9
G 3
stimulus
G 6
15 mM glucose
5 mM leucine
1.5 mM oleate/palmitate
6 mM glucose
30 mM KCl
Insulin Secretion (AUC)
B
Type 2 diabetes
0.0957
C
D
E
Time to peak insulin (min)
0.1590
0.0800
Glucose
Leucine
Oleate/Palmitate
ND
T2D

Figure S1

**Fig. S1. Related to Figure 1: Average dynamic insulin secretion perifusion AUC calculations in non-diabetic donors and donors with type-2 diabetes. (A)** Averaged traces of dynamic insulin secretion measurements in response to glucose (15 or 6 mM), leucine (5 mM), oleate and palmitate (1.5 mM. 1:1 mixture) or KCl (30 mM) in islets isolated from ND donors (n=123). Numbers in grey represent the following time intervals: 1. baseline (0-20 min) 2a. 1st phase (20-35 min) 2b. second phase (35-60 min) 3a. 3mM glucose (60-75 min) 3b. 3mM glucose (75-90 min) 4. 6 mM glucose (90-100 min) 5. 6 mM glucose (100-120 min) 6. 6 mM glucose (120-130 min) 7. 3mM glucose (130-160 min) 8. 30 mM KCl (160-180 min) and 9. 3 mM glucose (190-200 min). Corresponding areas under the curve (AUC) for each indicated section, with median and interquartile range are shown. **(B)** Shows the same as (A) except in islets from donors previously diagnosed with T2D (n=17). **(C)** Time-to-reach peak insulin secretion following a 15 mM glucose stimulus (brown), 5 mM leucine stimulus (orange) or 1.5 mM oleate/palmitate stimulus (blue) in non-diabetic donors (n=123). **(D)** Shows the same as (C) except in islets from donors diagnosed with type 2 diabetes (n=17). **(E)** Shows the direct comparisons of time-to-reach peak between non-diabetic donors (n=123) and donors with type-2 diabetes (n=17) for each macronutrient tested. * indicates $p<0.05$, ** indicates $p<0.01$, *** indicates $p<0.001$, and **** indicates $p<0.0001$.

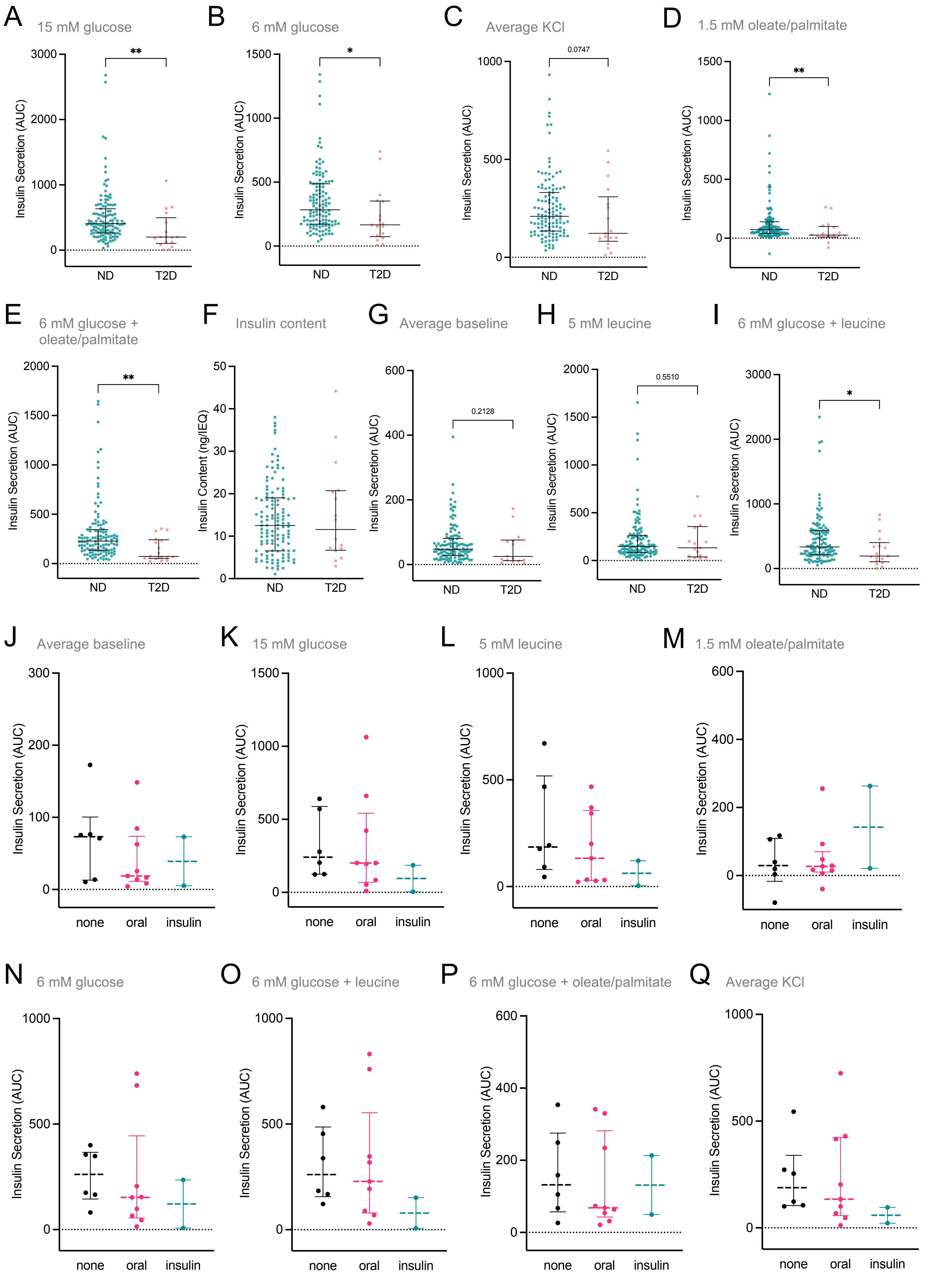

A
15 mM glucose
Insulin Secretion (AUC)
3000
2000
1000
0
**
ND
T2D
B
6 mM glucose
Insulin Secretion (AUC)
1500
1000
500
0
*
ND
T2D
C
Average KCl
Insulin Secretion (AUC)
1000
500
0
0.0747
ND
T2D
D
1.5 mM oleate/palmitate
Insulin Secretion (AUC)
1500
1000
500
0
**
ND
T2D
E
6 mM glucose + oleate/palmitate
Insulin Secretion (AUC)
2000
1500
1000
500
0
**
ND
T2D
F
Insulin content
Insulin Content (ng/IEQ)
50
40
30
20
10
0
ND
T2D
G
Average baseline
Insulin Secretion (AUC)
600
400
200
0
0.2128
ND
T2D
H
5 mM leucine
Insulin Secretion (AUC)
2000
1500
1000
500
0
0.5510
ND
T2D
I
6 mM glucose + leucine
Insulin Secretion (AUC)
3000
2000
1000
0
*
ND
T2D
J
Average baseline
Insulin Secretion (AUC)
300
200
100
0
none
oral
insulin
K
15 mM glucose
Insulin Secretion (AUC)
1500
1000
500
0
none
oral
insulin
L
5 mM leucine
Insulin Secretion (AUC)
1000
500
0
none
oral
insulin
M
1.5 mM oleate/palmitate
Insulin Secretion (AUC)
600
400
200
0
none
oral
insulin
N
6 mM glucose
Insulin Secretion (AUC)
1000
500
0
none
oral
insulin
O
6 mM glucose + leucine
Insulin Secretion (AUC)
1000
500
0
none
oral
insulin
P
6 mM glucose + oleate/palmitate
Insulin Secretion (AUC)
600
400
200
0
none
oral
insulin
Q
Average KCl
Insulin Secretion (AUC)
1000
500
0
none
oral
insulin


Figure S2

**Fig. S2. Related to Figure 1: Comparing nutrient-stimulated insulin secretion in ND donors and donors with T2D. (A-I)** Direct comparisons of AUC values, or islet insulin content (ng/IEQ) between ND donors and donors with T2D. **(J-Q)** Shows direct comparisons of AUC values of donors with T2D grouped by known medication status. “None” indicates that the donors were not taking any known glucose-lowering medications. “Oral” indicates that donors were taking oral hypoglycemic mediations (metformin in most cases). “Insulin” indicates that the donors had progressed to needing exogenous insulin for glucose control. Median and interquartile range are shown. * indicates p<0.05, and ** indicates p<0.01.

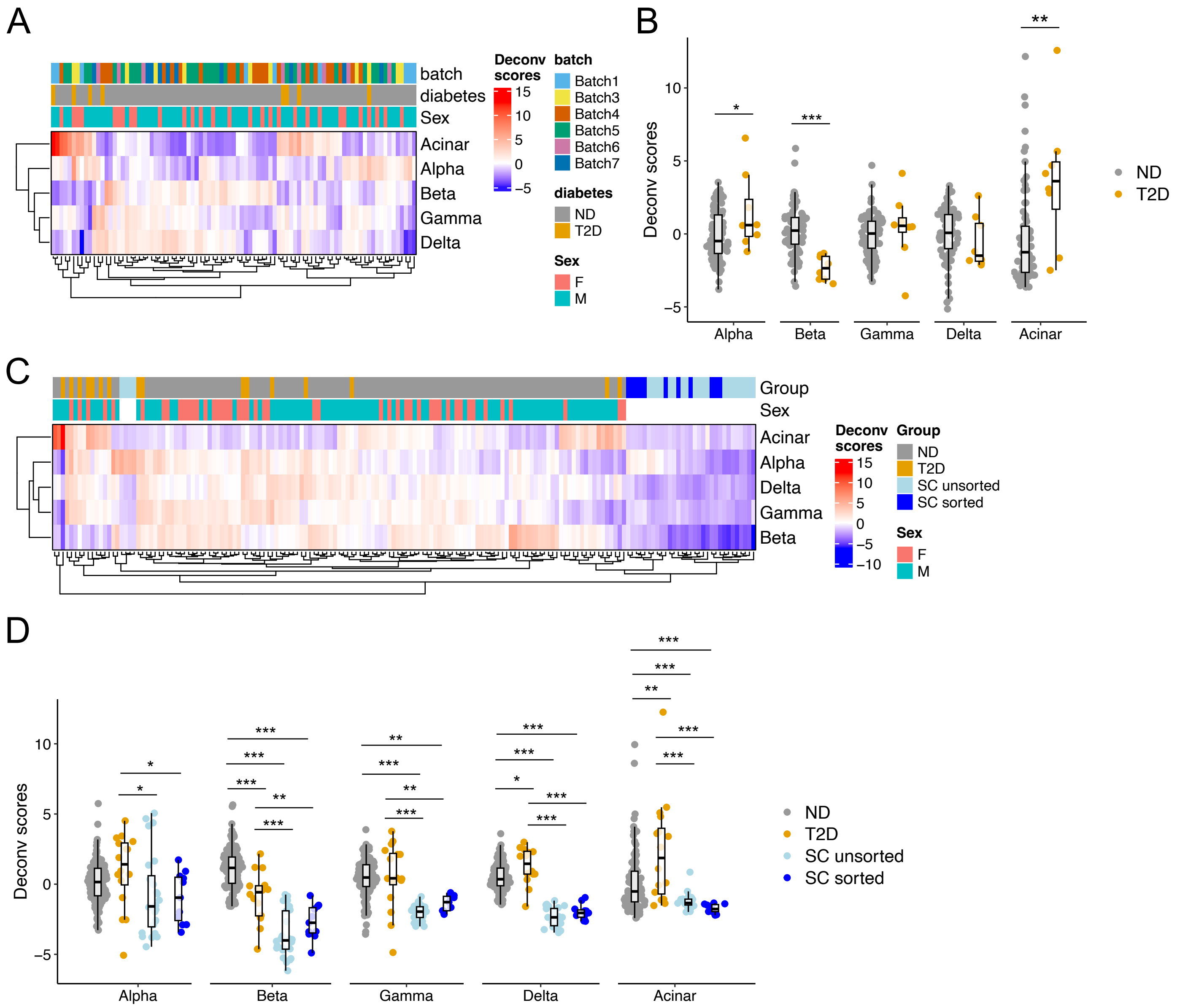


**Fig. S3. Related to Figure 1 and Figure 5: Islet cell type deconvolution scores of human islets and stem cell-derived islet clusters from RNAseq or proteomics. (A)** Heatmap showing the deconvolution scores from human islet RNAseq. Batches, diabetes status and sex are annotated on top. **(B)** Deconvolution scores from human islet RNAseq comparing ND and T2D donors. **(C)** Heatmap showing the deconvolution scores from human islet or stem cell (SC)-derived islet-like cluster proteomics. **(D)** Deconvolution scores from proteomics comparing ND and T2D human islets and unsorted or sorted stem cell (SC)-derived islet-clusters. * indicates p<0.05, ** indicates p<0.01, and *** indicates p<0.001.

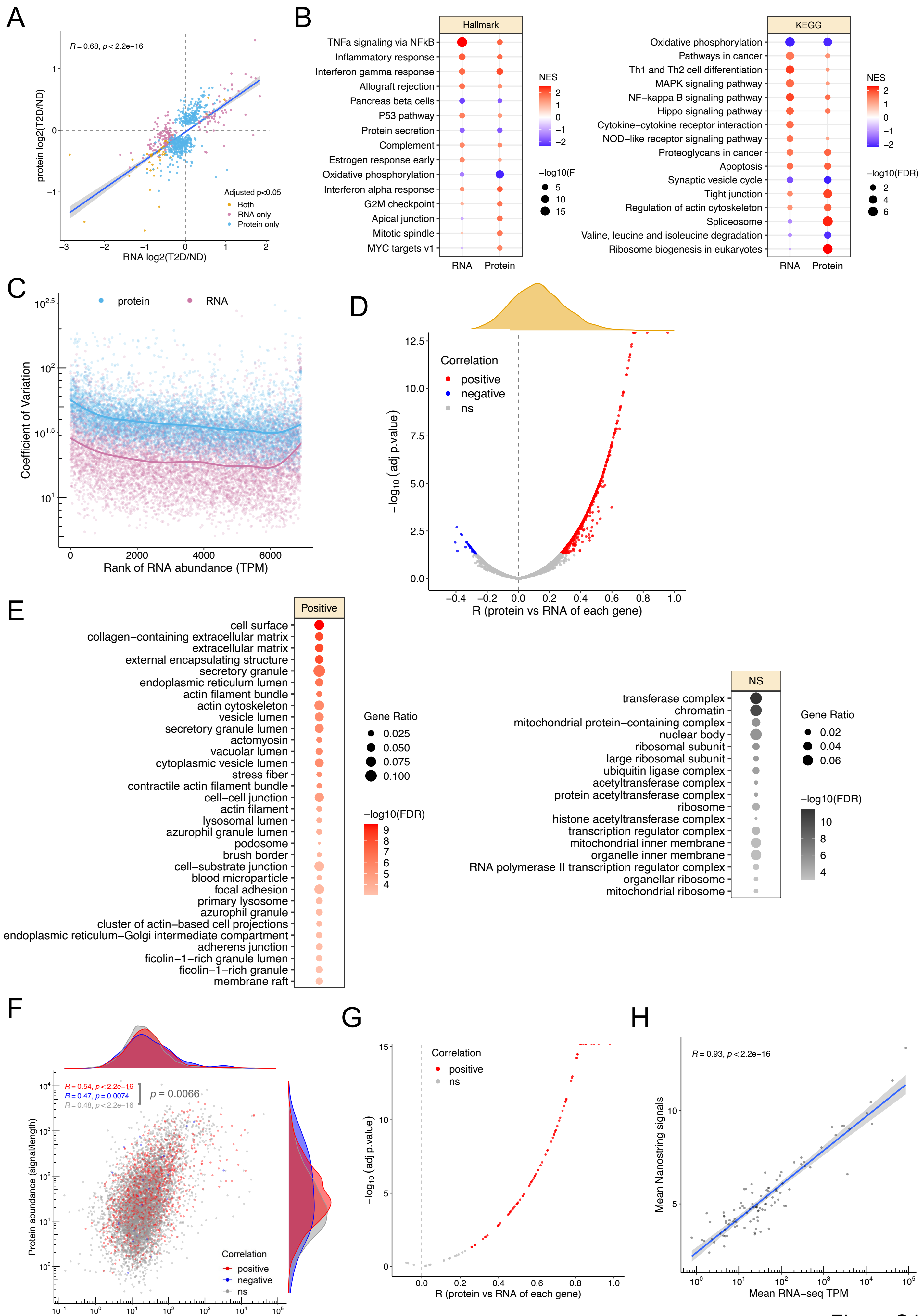

A
R = 0.68, p < 2.2e−16
protein log2(T2D/ND)
RNA log2(T2D/ND)
Adjusted p<0.05
Both
RNA only
Protein only
B
Hallmark
TNFa signaling via NFkB
Inflammatory response
Interferon gamma response
Allograft rejection
Pancreas beta cells
P53 pathway
Protein secretion
Complement
Estrogen response early
Oxidative phosphorylation
Interferon alpha response
G2M checkpoint
Apical junction
Mitotic spindle
MYC targets v1
RNA
Protein
NES
−log10(F
KEGG
Oxidative phosphorylation
Pathways in cancer
Th1 and Th2 cell differentiation
MAPK signaling pathway
NF−kappa B signaling pathway
Hippo signaling pathway
Cytokine−cytokine receptor interaction
NOD−like receptor signaling pathway
Proteoglycans in cancer
Apoptosis
Synaptic vesicle cycle
Tight junction
Regulation of actin cytoskeleton
Spliceosome
Valine, leucine and isoleucine degradation
Ribosome biogenesis in eukaryotes
−log10(FDR)
C
protein
RNA
Coefficient of Variation
Rank of RNA abundance (TPM)
D
Correlation
positive
negative
ns
−log10 (adj p.value)
R (protein vs RNA of each gene)
E
Positive
cell surface
collagen−containing extracellular matrix
extracellular matrix
external encapsulating structure
secretory granule
endoplasmic reticulum lumen
actin filament bundle
actin cytoskeleton
vesicle lumen
secretory granule lumen
actomyosin
vacuolar lumen
cytoplasmic vesicle lumen
stress fiber
contractile actin filament bundle
cell−cell junction
actin filament
lysosomal lumen
azurophil granule lumen
podosome
brush border
cell−substrate junction
blood microparticle
focal adhesion
primary lysosome
azurophil granule
cluster of actin−based cell projections
endoplasmic reticulum−Golgi intermediate compartment
adherens junction
ficolin−1−rich granule lumen
ficolin−1−rich granule
membrane raft
Gene Ratio
−log10(FDR)
NS
transferase complex
chromatin
mitochondrial protein−containing complex
nuclear body
ribosomal subunit
large ribosomal subunit
ubiquitin ligase complex
acetyltransferase complex
protein acetyltransferase complex
ribosome
histone acetyltransferase complex
transcription regulator complex
mitochondrial inner membrane
organelle inner membrane
RNA polymerase II transcription regulator complex
organellar ribosome
mitochondrial ribosome
Gene Ratio
−log10(FDR)
F
R = 0.54, p < 2.2e−16
R = 0.47, p = 0.0074
R = 0.48, p < 2.2e−16
p = 0.0066
Protein abundance (signal/length)
RNA abundance (mean TPM)
Correlation
positive
negative
ns
G
Correlation
positive
ns
−log10 (adj p.value)
R (protein vs RNA of each gene)
H
R = 0.93, p < 2.2e−16
Mean Nanostring signals
Mean RNA−seq TPM


Figure S4

**Fig. S4. Related to Figure 1: Comparative analyses of human islet transcriptomes and proteomes. (A)** Correlation between the log2 fold changes of differentially expressed RNAs and proteins (T2D vs ND, adjusted $p < 0.05$). **(B)** Hallmark and selected KEGG gene sets enriched from the transcriptomic or proteomic changes in T2D (T2D vs ND, FDR<0.005 from transcriptome or proteome). **(C)** Variance (Coefficient of Variation) of RNA and protein of each gene (ranked by the RNA abundance). **(D)** Within gene, across sample RNA-protein correlations among the 90 donor samples (82 ND and 8 T2D) and 7609 genes that were commonly measured in RNA-seq and proteomics. 1422 (19%) or 37 (0.5%) genes are positive or negatively correlated, respectively. **(E)** GO - Cellular Component (GO_CC) functional enrichment analysis of the gene sets enriched by the positive (left) or non-significant (right) RNA-protein correlations. (FDR<0.0001). **(F)** Across-gene correlation between RNAs and proteins among the genes that have positive, negative, or non-significant (ns) within-gene correlations in (D). Statistical difference between their correlation coefficient (R) were calculated using the cocor R package **(G)** Within gene, across sample correlation between RNAs measured by RNA-seq and NanoString. 82 out of the 99 commonly detected genes (83%) are positively correlated among the 64 common donor samples. **(H)** Across-gene correlation between RNAs measured by RNA-seq and NanoString.

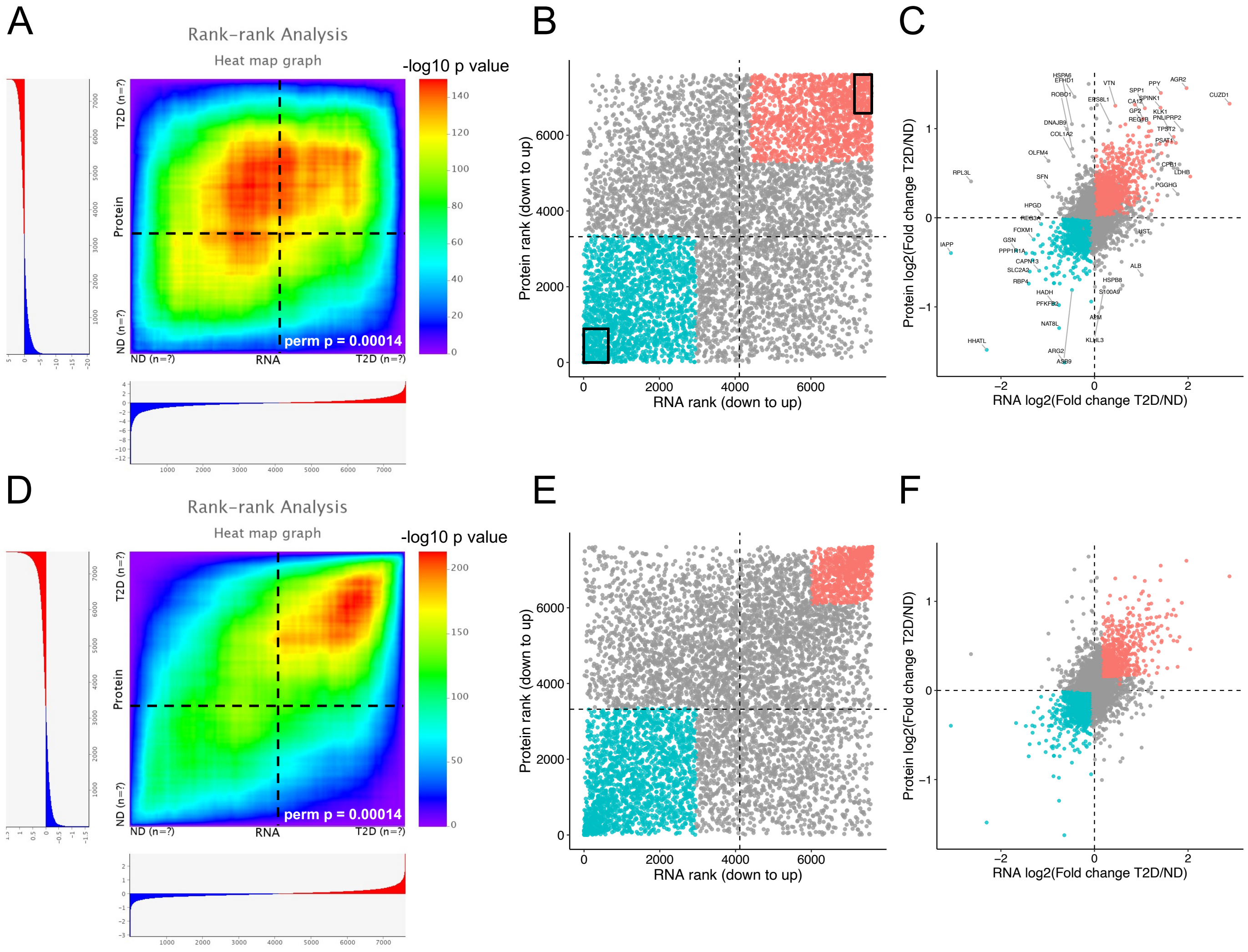


**Figure S5. Related to Figure 1: Rank-rank hypergeometric overlap (RRHO) test comparing proteomic and transcriptomic changes in T2D.** Proteins and RNAs commonly detected in our proteomics and RNA-seq datasets were ranked by –log10 p value **(A-C)** or by log2 fold change **(D-F)** in our differential expression analysis (T2D/ND). **(A)** Hypergeometric overlap map showing the hypergeometric p values (adjusted by Benjamini-Yekutieli FDR) at each rank threshold. **(B)** Rank-Rank scatter plot with the most statistically significant overlapping gene set colored in pink (~1500 up-up) or blue (~1800 down-down). Proteins and RNAs within the black square were altered in T2D (using a less stringent cut off - unadjusted p value < 0.05 in differential expression analysis), which are only a small subset of the overlapping gene set determined by RRHO. **(C)** The log2 fold changes of proteins and RNAs with the most statistically significant overlapping gene set colored in pink (up-up) or blue (down-down). **(D-F)** Same as (A-C) but using log2 fold changes to rank the proteins and RNAs in RRHO test. In E there are are ~800 up-up and ~1800 down-down overlapping gene sets.

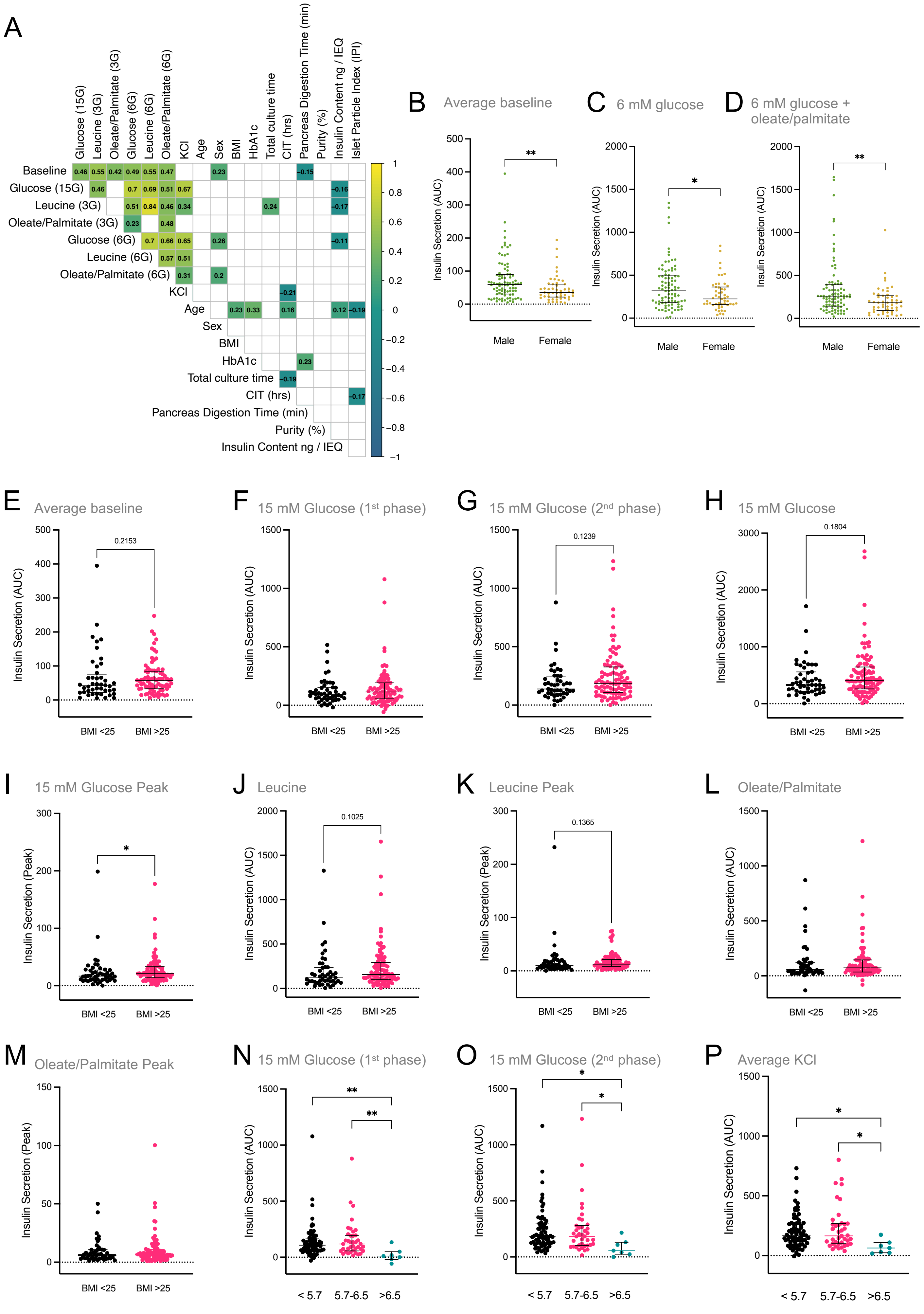
A
Baseline
Glucose (15G)
Leucine (3G)
Oleate/Palmitate (3G)
Glucose (6G)
Leucine (6G)
Oleate/Palmitate (6G)
KCl
Age
Sex
BMI
HbA1c
Total culture time
CIT (hrs)
Pancreas Digestion Time (min)
Purity (%)
Insulin Content ng / IEQ
Islet Particle Index (IPI)
B Average baseline
C 6 mM glucose
D 6 mM glucose + oleate/palmitate
Insulin Secretion (AUC)
Male
Female
E Average baseline
F 15 mM Glucose (1st phase)
G 15 mM Glucose (2nd phase)
H 15 mM Glucose
I 15 mM Glucose Peak
J Leucine
K Leucine Peak
L Oleate/Palmitate
M Oleate/Palmitate Peak
N 15 mM Glucose (1st phase)
O 15 mM Glucose (2nd phase)
P Average KCl
Insulin Secretion (Peak)
BMI <25
BMI >25
0.2153
0.1239
0.1804
0.1025
0.1365
< 5.7
5.7-6.5
>6.5

Figure S6

**Fig. S6. Related to Figure 1 and Figure 2: Donor metrics and islet function. (A)** Multiple comparisons matrix showing correlations between indicated parameters. Only significant R-values are shown. **(B-D)** Shows direct comparisons of AUC values of indicated areas of the perifusion curve. between all donors separated by biological sex (male (n=89) and female (n=51)). Only measurements that show significant differences between the male and female donors are shown. **(E-M)** Shows direct comparisons of selected AUC values or peak values of indicated areas of the perifusion curve between all donors separated by BMI. **(N-P)** Shows direct comparisons of 1st and 2nd phase 15 mM glucose-stimulated insulin secretion as well as KCl-stimulated insulin secretion between all donors stratified by HbA1c. * indicates $p<0.05$, ** indicates $p<0.01$.

A

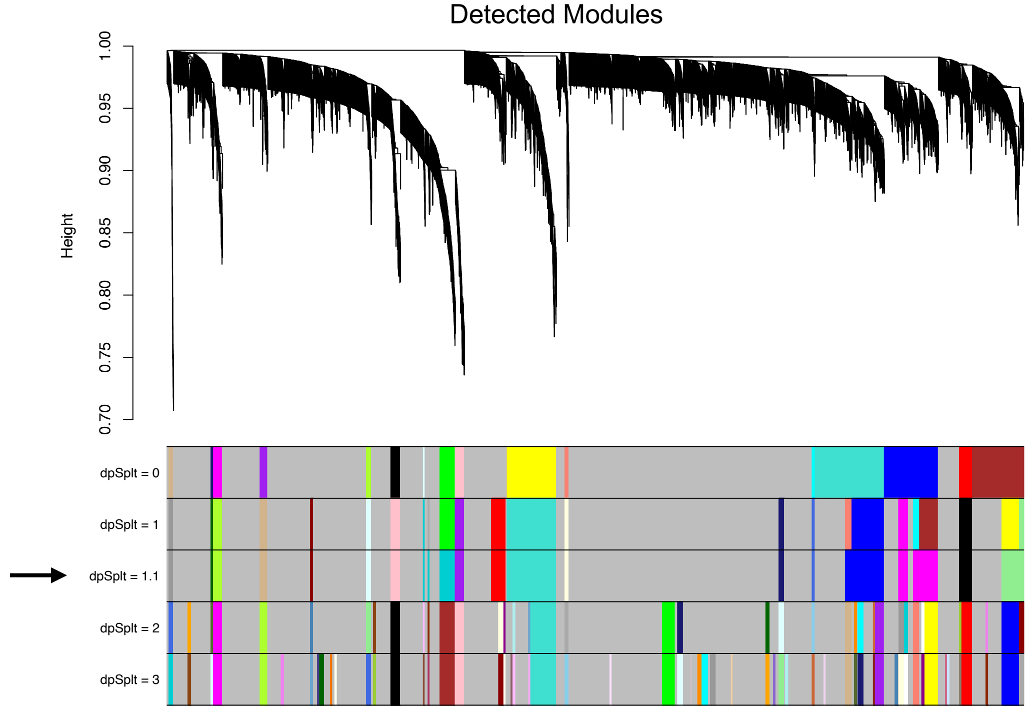

Detected Modules
Height
1.00
0.95
0.90
0.85
0.80
0.75
0.70
dpSplt = 0
dpSplt = 1
dpSplt = 1.1
dpSplt = 2
dpSplt = 3


B

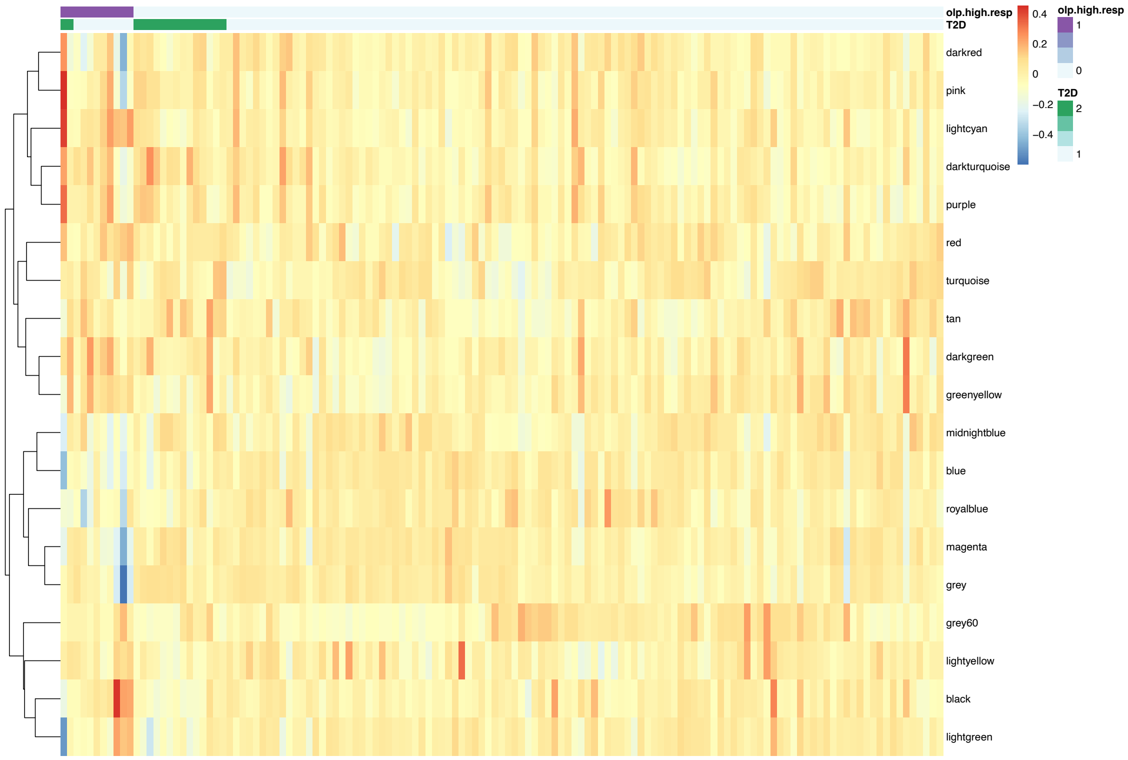

olp.high.resp
T2D
darkred
pink
lightcyan
darkturquoise
purple
red
turquoise
tan
darkgreen
greenyellow
midnightblue
blue
royalblue
magenta
grey
grey60
lightyellow
black
lightgreen
0.4
0.2
0
−0.2
−0.4
olp.high.resp
1
0
T2D
2
1


Figure S7

**Fig. S7. Related to Figure 2: Detailed proteomics co-expression network results. (A)** Diagram showing hierarchically clustered proteins and their module membership. The height of the diagram corresponds to the dissimilarity of the two merged proteins for each node in the dendrogram. The bands below the dendrogram show detected modules for 4 different values of the 'deepSplit' parameter (dpSplit = 0, 1, 2, 3), as well as modules merged based on the correlation of their eigengenes for deepSplit parameter = 1 (dpSplit = 1.1; these module definitions were used for all downstream analyses). **(B)** Heatmap of module eigengenes for all donors. Donors are annotated with whether they were oleate palmitate high responders (purple = yes, light blue = no) and whether they were diagnosed with T2D (green = yes, light blue = no). Modules (rows) are hierarchically clustered while donors (columns) are sorted first according to whether they are oleate palmitate high responders, and second according to whether they were reported to have T2D.

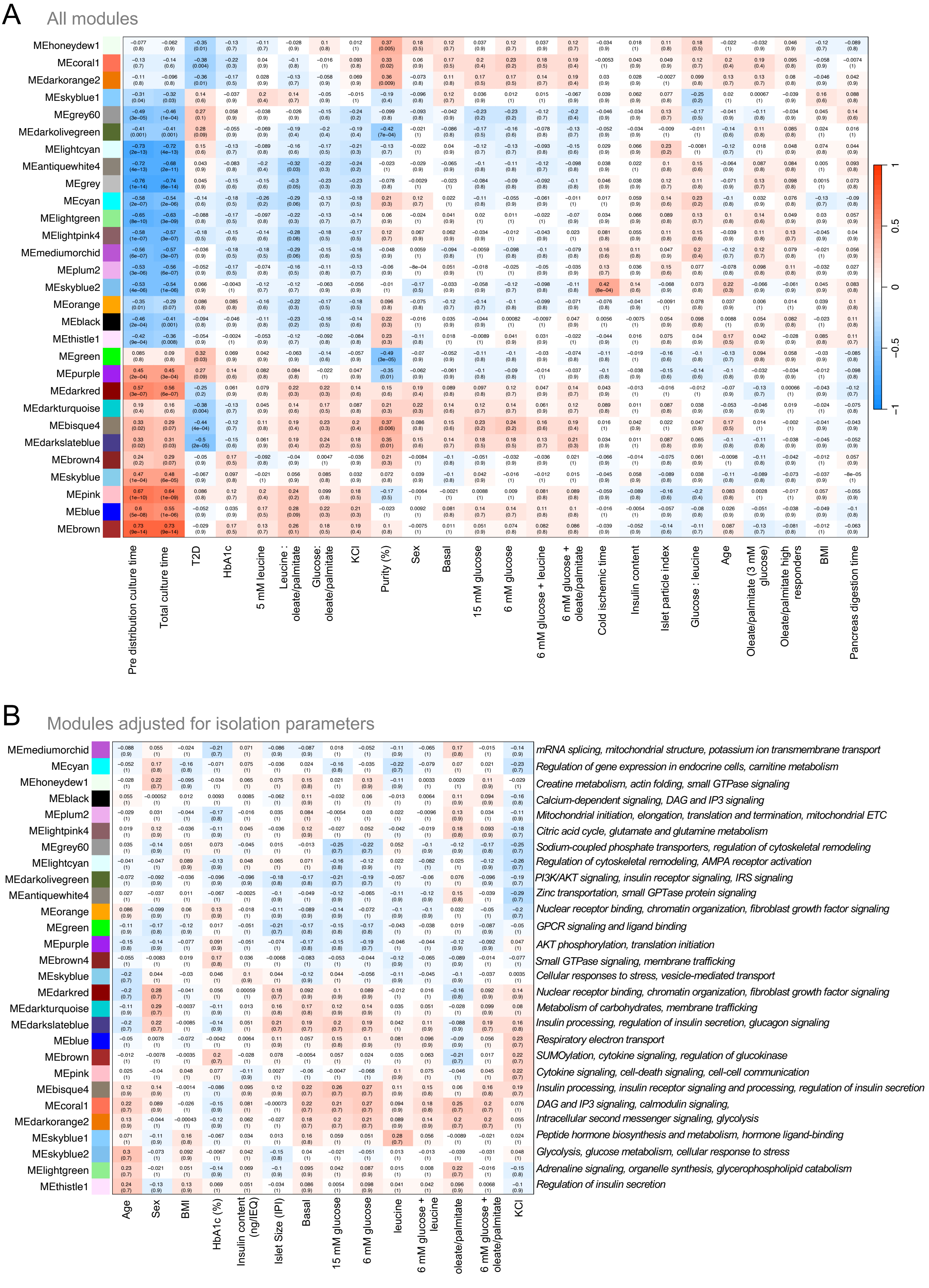
A
All modules
MEhoneydew1
MEcoral1
MEdarkorange2
MEskyblue1
MEgrey60
MEdarkolivegreen
MElightcyan
MEantiquewhite4
MEgrey
MEcyan
MElightgreen
MElightpink4
MEmediumorchid
MEplum2
MEskyblue2
MEorange
MEblack
MEthistle1
MEgreen
MEpurple
MEdarkred
MEdarkturquoise
MEbisque4
MEdarkslateblue
MEbrown4
MEskyblue
MEpink
MEblue
MEbrown
Pre distribution culture time
Total culture time
T2D
HbA1c
5 mM leucine
Leucine : oleate/palmitate
Glucose: oleate/palmitate
KCl
Purity (%)
Sex
Basal
15 mM glucose
6 mM glucose
6 mM glucose + leucine
6 mM glucose + oleate/palmitate
Cold ischemic time
Insulin content
Islet particle index
Glucose : leucine
Age
Oleate/palmitate (3 mM glucose)
Oleate/palmitate high responders
BMI
Pancreas digestion time
1
0.5
0
−0.5
−1
B
Modules adjusted for isolation parameters
MEmediumorchid
MEcyan
MEhoneydew1
MEblack
MEplum2
MElightpink4
MEgrey60
MElightcyan
MEdarkolivegreen
MEantiquewhite4
MEorange
MEgreen
MEpurple
MEbrown4
MEskyblue
MEdarkred
MEdarkturquoise
MEdarkslateblue
MEblue
MEbrown
MEpink
MEbisque4
MEcoral1
MEdarkorange2
MEskyblue1
MEskyblue2
MElightgreen
MEthistle1
mRNA splicing, mitochondrial structure, potassium ion transmembrane transport
Regulation of gene expression in endocrine cells, carnitine metabolism
Creatine metabolism, actin folding, small GTPase signaling
Calcium-dependent signaling, DAG and IP3 signaling
Mitochondrial initiation, elongation, translation and termination, mitochondrial ETC
Citric acid cycle, glutamate and glutamine metabolism
Sodium-coupled phosphate transporters, regulation of cytoskeletal remodeling
Regulation of cytoskeletal remodeling, AMPA receptor activation
PI3K/AKT signaling, insulin receptor signaling, IRS signaling
Zinc transportation, small GPTase protein signaling
Nuclear receptor binding, chromatin organization, fibroblast growth factor signaling
GPCR signaling and ligand binding
AKT phosphorylation, translation initiation
Small GTPase signaling, membrane trafficking
Cellular responses to stress, vesicle-mediated transport
Nuclear receptor binding, chromatin organization, fibroblast growth factor signaling
Metabolism of carbohydrates, membrane trafficking
Insulin processing, regulation of insulin secretion, glucagon signaling
Respiratory electron transport
SUMOylation, cytokine signaling, regulation of glucokinase
Cytokine signaling, cell-death signaling, cell-cell communication
Insulin processing, insulin receptor signaling and processing, regulation of insulin secretion
DAG and IP3 signaling, calmodulin signaling,
Intracellular second messenger signaling, glycolysis
Peptide hormone biosynthesis and metabolism, hormone ligand-binding
Glycolysis, glucose metabolism, cellular response to stress
Adrenaline signaling, organelle synthesis, glycerophospholipid catabolism
Regulation of insulin secretion
Age
Sex
BMI
HbA1c (%)
Insulin content (ng/IEQ)
Islet Size (IPI)
Basal
15 mM glucose
6 mM glucose
leucine
6 mM glucose + leucine
oleate/palmitate
6 mM glucose + oleate/palmitate
KCl

Figure S8

**Fig. S8. Related to Figure 2: Association between RNAseq modules and donor metadata.** Heatmap of module - metadata associations, with pairwise correlations computed as **(A)** the Pearson correlation between module eigengenes and metadata values, and **(B)** the partial Pearson correlation between module eigengenes and metadata values other than technical isolation parameters (culture time, digestion time, cold ischemia time, and percent purity), with these parameters included as covariates in the partial correlation calculations. Cell color corresponds to correlation value, and each cell is annotated with the correlation value and FDR-adjusted p-value (FDR = 0.05) in brackets. Heatmap row (modules) and columns (metadata values) are hierarchically clustered based on correlation magnitude. Modules in B) are additionally labeled with representative functional themes from overrepresentation gene set analysis with the KEGG, Reactome, Gene Ontology Cellular Component, Molecular Function, and Biological Process functional libraries.

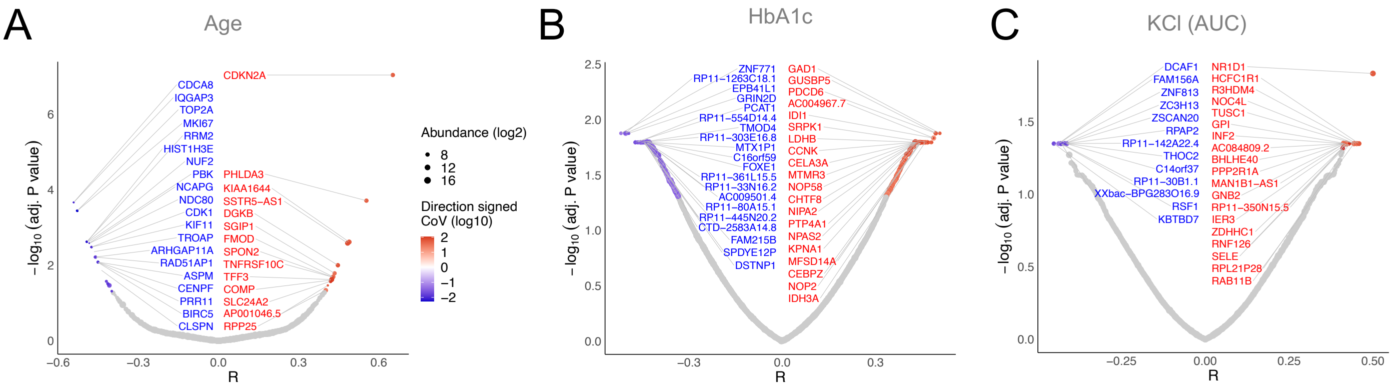


**Fig. S9. Related to Figure 3: RNAseq versus donor metadata and islet function.** Volcano plots for mRNAs significantly negatively (blue) or positively (red) correlated to **(A)** donor age, **(B)** donor HbA1c, and **(C)** insulin secretion stimulated by direct depolarization with KCl (30 mM). Protein abundances (log2) are depicted by size of circle, and the coefficient of variance (log10) is depicted by color gradient. Volcano plots are only shown if significant correlations were found.

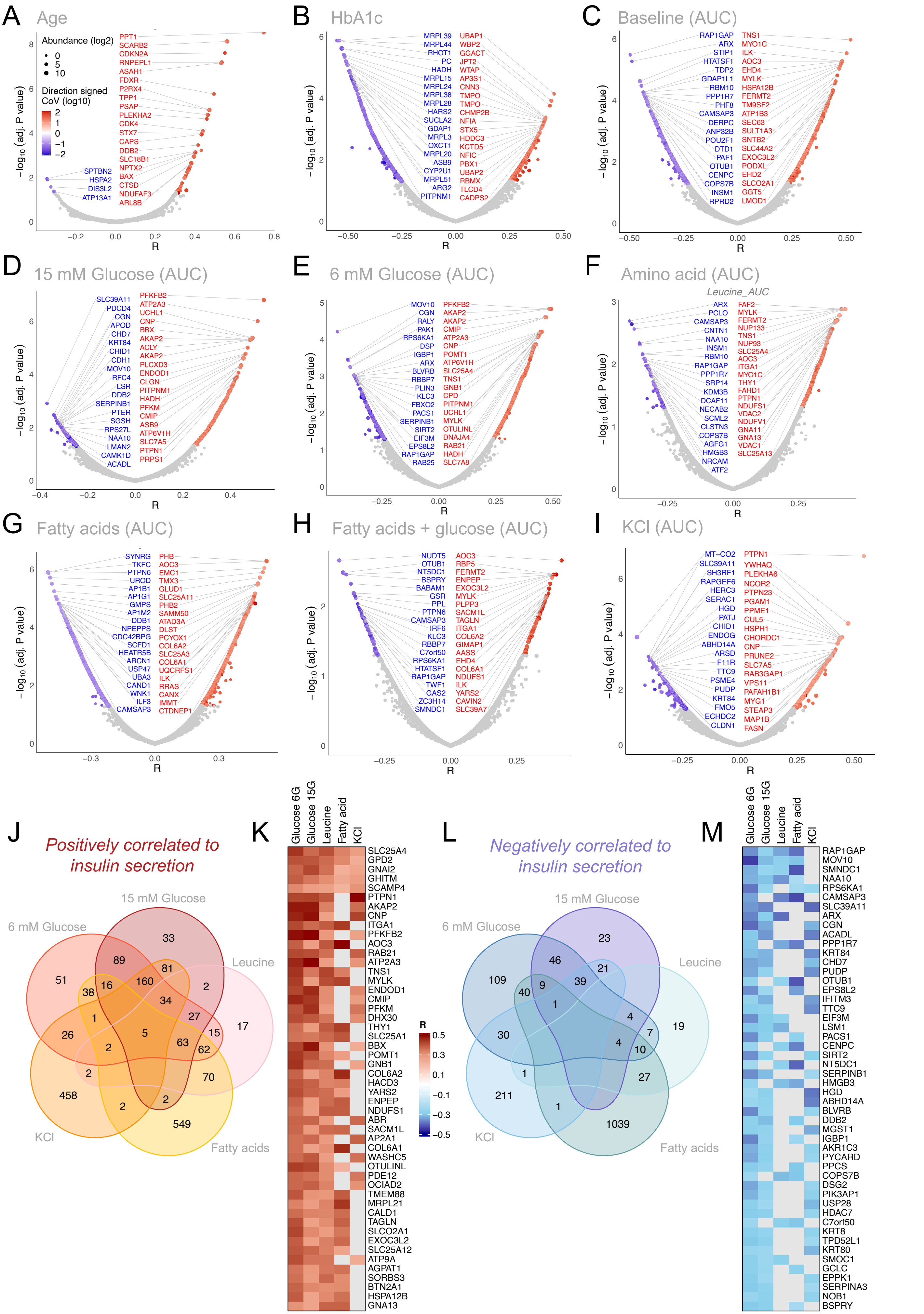

A Age
Abundance (log2)
0
5
10
Direction signed CoV (log10)
2
1
0
−1
−2
PPT1 SCARB2 CDKN2A RNPEPL1 ASAH1 FDXR P2RX4 TPP1 PSAP PLEKHA2 CDK4 STX7 CAPS DDB2 SLC18B1 NPTX2 BAX CTSD NDUFAF3 ARL8B
SPTBN2 HSPA2 DIS3L2 ATP13A1
−log10 (adj. P value)
R
B HbA1c
MRPL39 MRPL44 RHOT1 PC HADH MRPL15 MRPL24 MRPL38 MRPL28 HARS2 SUCLA2 GDAP1 MRPL3 OXCT1 MRPL20 ASB9 CYP2U1 MRPL51 ARG2 PITPNM1
UBAP1 WBP2 GGACT JPT2 WTAP AP3S1 CNN3 TMPO TMPO CHMP2B NFIA STX5 HDDC3 KCTD5 NFIC PBX1 UBAP2 RBMX TLCD4 CADPS2
C Baseline (AUC)
RAP1GAP ARX STIP1 HTATSF1 TDP2 GDAP1L1 RBM10 PPP1R7 PHF8 CAMSAP3 DERPC ANP32B POU2F1 DTD1 PAF1 OTUB1 CENPC COPS7B INSM1 RPRD2
TNS1 MYO1C ILK AOC3 EHD4 MYLK HSPA12B FERMT2 TM9SF2 ATP1B3 SEC63 SULT1A3 SNTB2 SLC44A2 EXOC3L2 PODXL EHD2 SLCO2A1 GGT5 LMOD1
D 15 mM Glucose (AUC)
SLC39A11 PDCD4 CGN APOD CHD7 KRT84 CHID1 CDH1 MOV10 RFC4 LSR DDB2 SERPINB1 PTER SGSH RPS27L NAA10 LMAN2 CAMK1D ACADL
PFKFB2 ATP2A3 UCHL1 CNP BBX AKAP2 ACLY AKAP2 PLCXD3 ENDOD1 CLGN PITPNM1 HADH PFKM CMIP ASB9 ATP6V1H SLC7A5 PTPN1 PRPS1
E 6 mM Glucose (AUC)
MOV10 CGN RALY PAK1 RPS6KA1 DSP IGBP1 ARX BLVRB RBBP7 PLIN3 KLC3 FBXO2 PACS1 SERPINB1 SIRT2 EIF3M EPS8L2 RAP1GAP RAB25
PFKFB2 AKAP2 AKAP2 CMIP ATP2A3 CNP POMT1 ATP6V1H SLC25A4 TNS1 GNB1 CPD PITPNM1 UCHL1 MYLK OTULINL DNAJA4 RAB21 HADH SLC7A8
F Amino acid (AUC)
Leucine_AUC
ARX PCLO CAMSAP3 CNTN1 NAA10 INSM1 RBM10 RAP1GAP PPP1R7 SRP14 KDM3B DCAF11 NECAB2 SCML2 CLSTN3 COPS7B AGFG1 HMGB3 NRCAM ATF2
FAF2 MYLK FERMT2 NUP133 TNS1 NUP93 SLC25A4 AOC3 ITGA1 MYO1C THY1 FAHD1 PTPN1 NDUFS1 VDAC2 NDUFV1 GNA11 GNA13 VDAC1 SLC25A13
G Fatty acids (AUC)
SYNRG TKFC PTPN6 UROD AP1B1 AP1G1 GMPS AP1M2 DDB1 NPEPPS CDC42BPG SCFD1 HEATR5B ARCN1 USP47 UBA3 CAND1 WNK1 ILF3 CAMSAP3
PHB AOC3 EMC1 TMX3 GLUD1 SLC25A11 PHB2 SAMM50 ATAD3A DLST PCYOX1 COL6A2 SLC25A3 COL6A1 UQCRFS1 ILK RRAS CANX IMMT CTDNEP1
H Fatty acids + glucose (AUC)
NUDT5 OTUB1 NT5DC1 BSPRY BABAM1 GSR PPL PTPN6 CAMSAP3 IRF6 KLC3 RBBP7 C7orf50 RPS6KA1 HTATSF1 RAP1GAP TWF1 GAS2 ZC3H14 SMNDC1
AOC3 RBP5 FERMT2 ENPEP EXOC3L2 MYLK PLPP3 SACM1L TAGLN ITGA1 COL6A2 GIMAP1 AASS EHD4 COL6A1 NDUFS1 ILK YARS2 CAVIN2 SLC39A7
I KCl (AUC)
MT−CO2 SLC39A11 SH3RF1 RAPGEF6 HERC3 SERAC1 HGD PATJ CHID1 ENDOG ABHD14A ARSD F11R TTC9 PSME4 PUDP KRT84 FMO5 ECHDC2 CLDN1
PTPN1 YWHAQ PLEKHA6 NCOR2 PTPN23 PGAM1 PPME1 CUL5 HSPH1 CHORDC1 CNP PRUNE2 SLC7A5 RAB3GAP1 VPS11 PAFAH1B1 MYG1 STEAP3 MAP1B FASN
J Positively correlated to insulin secretion
15 mM Glucose
6 mM Glucose
Leucine
KCl
Fatty acids
33 89 51 81 38 16 160 2 34 1 27 17 26 5 15 63 62 2 2 70 458 2 2 549
K
Glucose 6G Glucose 15G Leucine Fatty acid KCl
SLC25A4 GPD2 GNAI2 GHITM SCAMP4 PTPN1 AKAP2 CNP ITGA1 PFKFB2 AOC3 RAB21 ATP2A3 TNS1 MYLK ENDOD1 CMIP PFKM DHX30 THY1 SLC25A1 BBX POMT1 GNB1 COL6A2 HACD3 YARS2 ENPEP NDUFS1 ABR SACM1L AP2A1 COL6A1 WASHC5 OTULINL PDE12 OCIAD2 TMEM88 MRPL21 CALD1 TAGLN SLCO2A1 EXOC3L2 SLC25A12 ATP9A AGPAT1 SORBS3 BTN2A1 HSPA12B GNA13
R
0.5 0.3 0.1 −0.1 −0.3 −0.5
L Negatively correlated to insulin secretion
15 mM Glucose
6 mM Glucose
Leucine
KCl
Fatty acids
23 46 109 21 40 9 39 1 4 19 30 7 4 10 1 27 211 1 1039
M
Glucose 6G Glucose 15G Leucine Fatty acid KCl
RAP1GAP MOV10 SMNDC1 NAA10 RPS6KA1 CAMSAP3 SLC39A11 ARX CGN ACADL PPP1R7 KRT84 CHD7 PUDP OTUB1 EPS8L2 IFITM3 TTC9 EIF3M LSM1 PACS1 CENPC SIRT2 NT5DC1 SERPINB1 HMGB3 HGD ABHD14A BLVRB DDB2 MGST1 IGBP1 AKR1C3 PYCARD PPCS COPS7B DSG2 PIK3AP1 USP28 HDAC7 C7orf50 KRT8 TPD52L1 KRT80 SMOC1 GCLC EPPK1 SERPINA3 NOB1 BSPRY


Figure S10

**Fig. S10. Related to Figure 3: Correlations of individual proteins and mRNAs with insulin secretory responses.** Volcano plots are shown depicting significant positive (red) and negative (blue) correlations of proteins to continuous donor characteristics: **(A)** donor age and **(B)** HbA1c; or functional parameters in response to: **(C)** 3 mM glucose **(D)** 15 mM glucose **(E)** 6 mM glucose **(F)** 5 mM leucine **(G)** 1.5 mM oleate/palmitate (1:1 mix) **(H)** 1.5 mM oleate/palmitate + 6 mM glucose and **(I)** 30 mM KCl. Protein abundances (log2) are depicted by size of circle, and the coefficient of variance (log10) is depicted by color gradient. **(J)** Venn diagram showing the overlap of the number of protein abundances that positively correlate to the indicated nutrient stimuli. **(K)** Heat map depicts the top 50 most positively correlative proteins to the indicated stimuli. **(L-M)** Shows the same as (J-K), but for negative correlations.

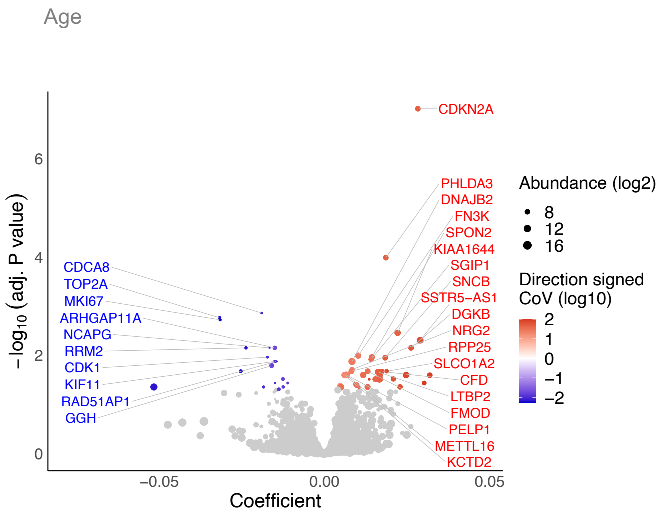


**Fig. S11. Related to Figure 3: Association between RNAseq donor metadata and islet function.** Volcano plot depicting significant positive (red) and negative (blue) linear regression coefficients (adjusting for T2D status) of transcripts associated with donor age. RNA expression (abundances) are depicted by size of circle (log2), and the coefficient of variation (log10) is depicted by color gradient. Volcano plots are only shown if significant associations were found.

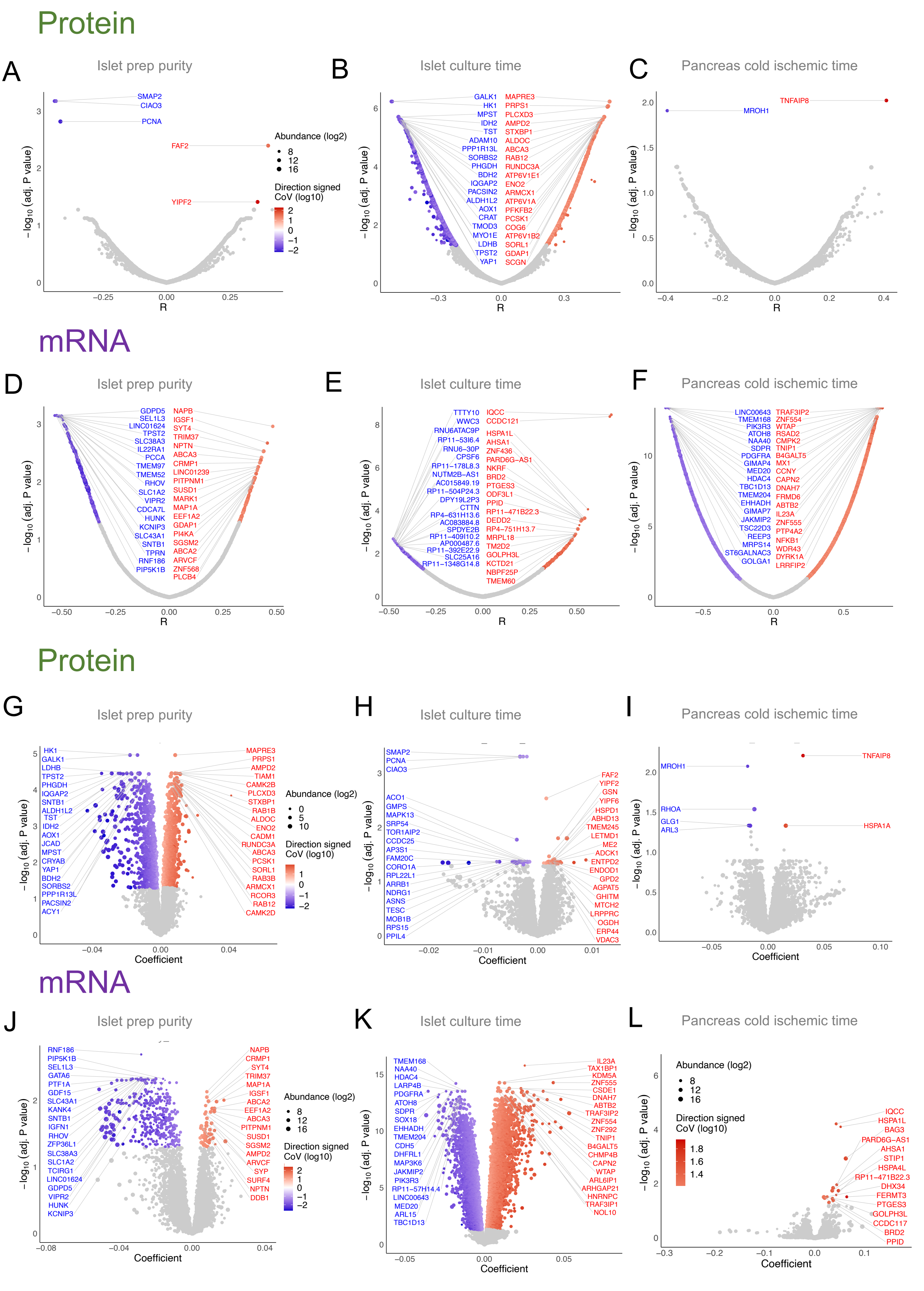
Protein
A
Islet prep purity
SMAP2
CIAO3
PCNA
FAF2
YIPF2
Abundance (log2)
8
12
16
Direction signed CoV (log10)
2
1
0
−1
−2
−log10 (adj. P value)
R
B
Islet culture time
GALK1
HK1
MPST
IDH2
TST
ADAM10
PPP1R13L
SORBS2
PHGDH
BDH2
IQGAP2
PACSIN2
ALDH1L2
AOX1
CRAT
TMOD3
MYO1E
LDHB
TPST2
YAP1
MAPRE3
PRPS1
PLCXD3
AMPD2
STXBP1
ALDOC
ABCA3
RAB12
RUNDC3A
ATP6V1E1
ENO2
ARMCX1
ATP6V1A
PFKFB2
PCSK1
COG6
ATP6V1B2
SORL1
GDAP1
SCGN
C
Pancreas cold ischemic time
TNFAIP8
MROH1
mRNA
D
Islet prep purity
GDPD5
SEL1L3
LINC01624
TPST2
SLC38A3
IL22RA1
PCCA
TMEM97
TMEM52
RHOV
SLC1A2
VIPR2
CDCA7L
HUNK
KCNIP3
SLC43A1
SNTB1
TPRN
RNF186
PIP5K1B
NAPB
IGSF1
SYT4
TRIM37
NPTN
ABCA3
CRMP1
LINC01239
PITPNM1
SUSD1
MARK1
MAP1A
EEF1A2
GDAP1
PI4KA
SGSM2
ABCA2
ARVCF
ZNF568
PLCB4
E
Islet culture time
TTTY10
WWC3
RNU6ATAC9P
RP11−5316.4
RNU6−30P
CPSF6
RP11−178L8.3
NUTM2B−AS1
AC015849.19
RP11−504P24.3
DPY19L2P3
CTTN
RP4−631H13.6
AC083884.8
SPDYE2B
RP11−409I10.2
AP000487.6
RP11−392E22.9
SLC25A16
RP11−1348G14.8
IQCC
CCDC121
HSPA1L
AHSA1
ZNF436
PARD6G−AS1
NKRF
BRD2
PTGES3
ODF3L1
PPID
RP11−471B22.3
DEDD2
RP4−751H13.7
MRPL18
TM2D2
GOLPH3L
KCTD21
NBPF25P
TMEM60
F
Pancreas cold ischemic time
LINC00643
TMEM168
PIK3R3
ATOH8
NAA40
SDPR
PDGFRA
GIMAP4
MED20
HDAC4
TBC1D13
TMEM204
EHHADH
GIMAP7
JAKMIP2
TSC22D3
REEP3
MRPS14
ST6GALNAC3
GOLGA1
TRAF3IP2
ZNF554
WTAP
RSAD2
CMPK2
TNIP1
B4GALT5
MX1
CCNY
CAPN2
DNAH7
FRMD6
ABTB2
IL23A
ZNF555
PTP4A2
NFKB1
WDR43
DYRK1A
LRRFIP2
Protein
G
Islet prep purity
HK1
GALK1
LDHB
TPST2
PHGDH
IQGAP2
SNTB1
ALDH1L2
TST
IDH2
AOX1
JCAD
MPST
CRYAB
YAP1
BDH2
SORBS2
PPP1R13L
PACSIN2
ACY1
MAPRE3
PRPS1
AMPD2
TIAM1
CAMK2B
PLCXD3
STXBP1
RAB1B
ALDOC
ENO2
CADM1
RUNDC3A
ABCA3
PCSK1
SORL1
RAB3B
ARMCX1
RCOR3
RAB12
CAMK2D
Abundance (log2)
0
5
10
Direction signed CoV (log10)
Coefficient
H
Islet culture time
SMAP2
PCNA
CIAO3
ACO1
GMPS
MAPK13
SRP54
TOR1AIP2
CCDC25
AP3S1
FAM20C
CORO1A
RPL22L1
ARRB1
NDRG1
ASNS
TESC
MOB1B
RPS15
PPIL4
FAF2
YIPF2
GSN
YIPF6
HSPD1
ABHD13
TMEM245
LETMD1
ME2
ADCK1
ENTPD2
ENDOD1
GPD2
AGPAT5
GHITM
MTCH2
LRPPRC
OGDH
ERP44
VDAC3
I
Pancreas cold ischemic time
MROH1
TNFAIP8
RHOA
GLG1
ARL3
HSPA1A
mRNA
J
Islet prep purity
RNF186
PIP5K1B
SEL1L3
GATA6
PTF1A
GDF15
SLC43A1
KANK4
SNTB1
IGFN1
RHOV
ZFP36L1
SLC38A3
SLC1A2
TCIRG1
LINC01624
GDPD5
VIPR2
HUNK
KCNIP3
NAPB
CRMP1
SYT4
TRIM37
MAP1A
IGSF1
ABCA2
EEF1A2
ABCA3
PITPNM1
SUSD1
SGSM2
AMPD2
ARVCF
SYP
SURF4
NPTN
DDB1
K
Islet culture time
TMEM168
NAA40
HDAC4
LARP4B
PDGFRA
ATOH8
SDPR
SOX18
EHHADH
TMEM204
CDH5
DHFRL1
MAP3K6
JAKMIP2
PIK3R3
RP11−57H14.4
LINC00643
MED20
ARL15
TBC1D13
IL23A
TAX1BP1
KDM5A
ZNF555
CSDE1
DNAH7
ABTB2
TRAF3IP2
ZNF554
ZNF292
TNIP1
B4GALT5
CHMP4B
CAPN2
WTAP
ARL6IP1
ARHGAP21
HNRNPC
TRAF3IP1
NOL10
L
Pancreas cold ischemic time
Abundance (log2)
8
12
16
Direction signed CoV (log10)
1.8
1.6
1.4
IQCC
HSPA1L
BAG3
PARD6G−AS1
AHSA1
STIP1
HSPA4L
RP11−471B22.3
DHX34
FERMT3
PTGES3
GOLPH3L
CCDC117
BRD2
PPID
Coefficient

Figure S12

**Fig. S12. Related to Figure 3: Proteome and transcriptome versus islet isolation parameters.** Volcano plots for proteins significantly negatively (blue) or positively (red) correlated to **(A)** islet preparation purity, **(B)** total islet culture time and **(C)** cold ischemic time of the pancreas. **(D-F)** Shows the same as (A-C) except for correlations with the transcriptome. Protein abundances (log2) are depicted by size of circle, and the coefficient of variance (log10) is depicted by color gradient. All donors (independent of diabetes diagnosis) were used in these analyses. **(G-I)** depicts volcano plots with significant positive (red) and negative (blue) linear regression coefficients (adjusting for T2D status) of proteins to islet preparation purity, total islet culture time and cold ischemic time of the pancreas. **(J-L)** Shows the same as (G-I) except for associations with the transcriptome. Protein and transcript abundances (log2) are depicted by size of circle, and the coefficient of variation (log10) is depicted by color gradient.

A

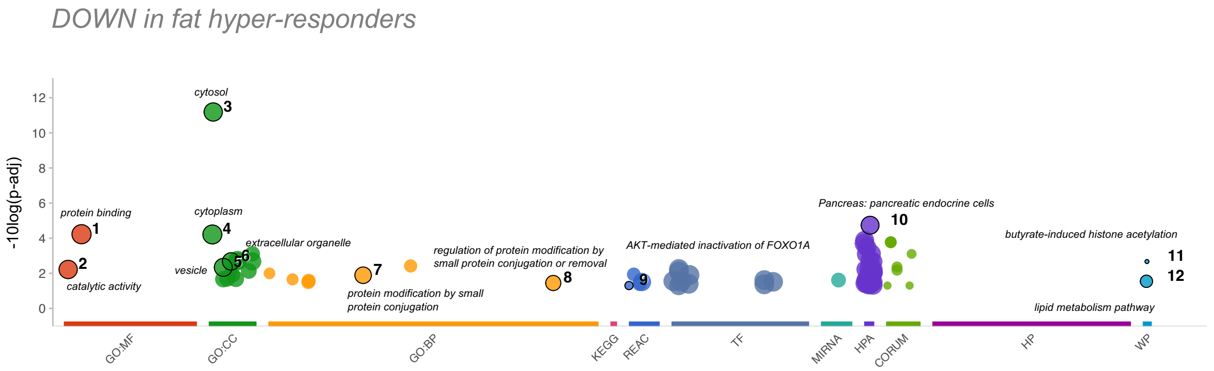


B

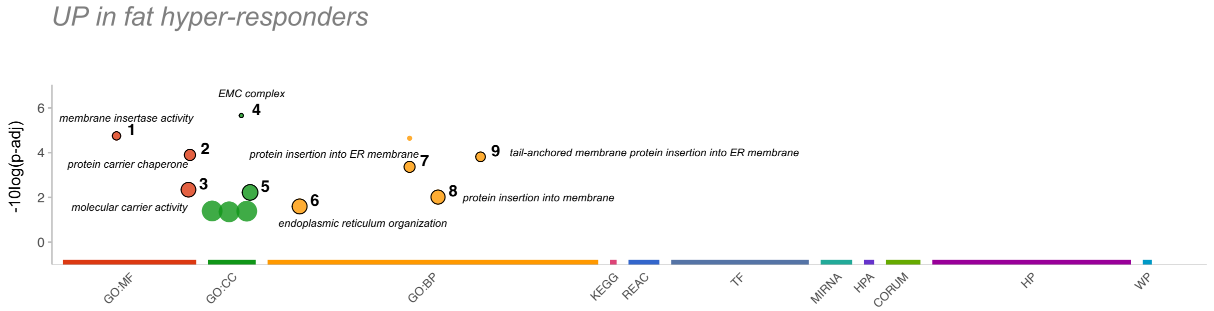


**Fig. S13. Related to Figure 4: Pathway analysis of differentially abundant proteins in fat hyper-responders. (A)** Pathway analysis of proteins that are less abundant in fat hyper-responsive donors showing enriched biological functions and pathways obtained from multiple databases including (among others), Gene Ontology (GO), KEGG and Reactome. **(B)** Shows the same as (A) except for proteins that are more abundant in fat hyper-responsive donors.

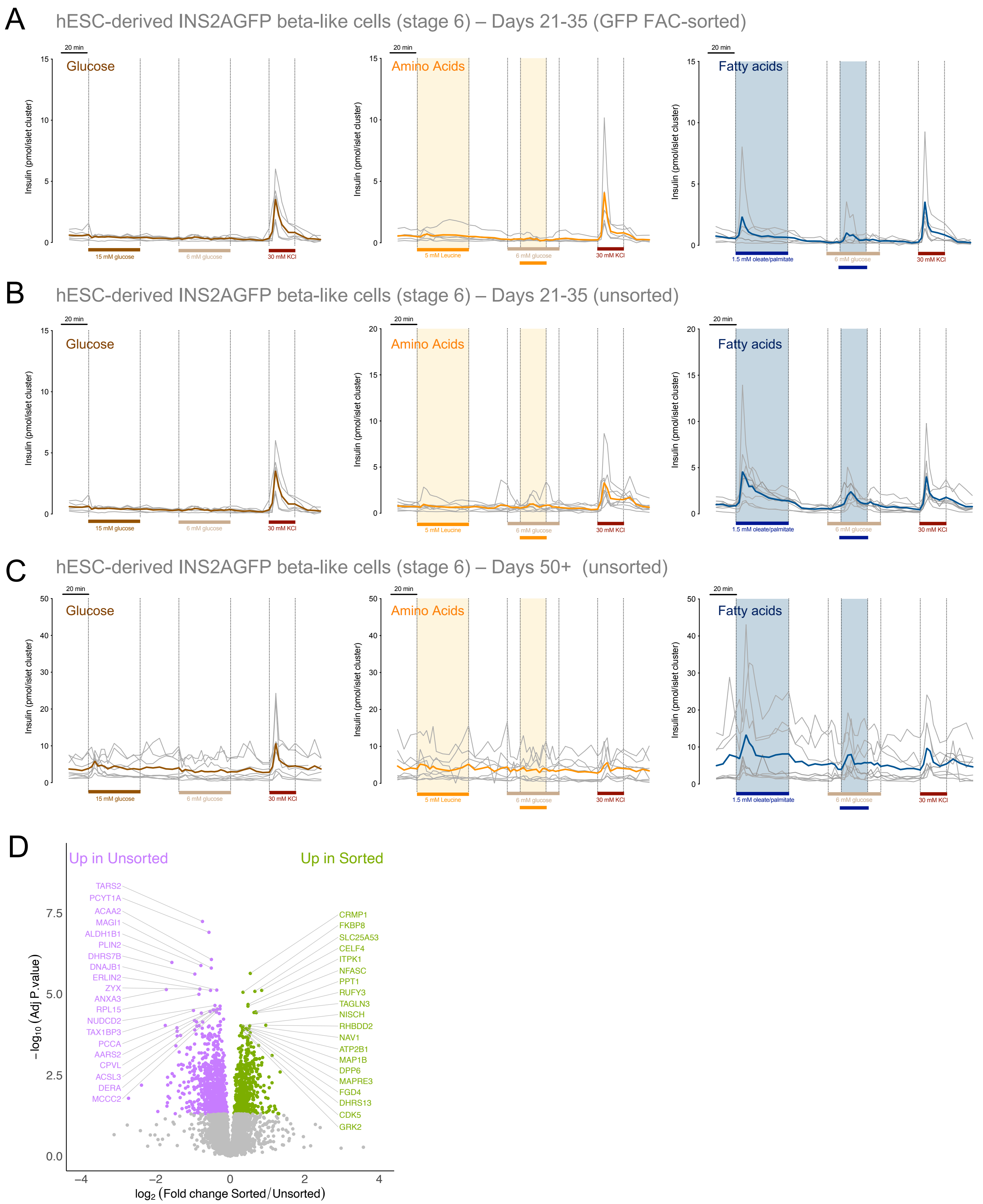
A
hESC-derived INS2AGFP beta-like cells (stage 6) – Days 21-35 (GFP FAC-sorted)
B
hESC-derived INS2AGFP beta-like cells (stage 6) – Days 21-35 (unsorted)
C
hESC-derived INS2AGFP beta-like cells (stage 6) – Days 50+ (unsorted)
20 min
Glucose
Amino Acids
Fatty acids
Insulin (pmol/islet cluster)
15 mM glucose
6 mM glucose
30 mM KCl
5 mM Leucine
1.5 mM oleate/palmitate
D
Up in Unsorted
Up in Sorted
TARS2
PCYT1A
ACAA2
MAGI1
ALDH1B1
PLIN2
DHRS7B
DNAJB1
ERLIN2
ZYX
ANXA3
RPL15
NUDCD2
TAX1BP3
PCCA
AARS2
CPVL
ACSL3
DERA
MCCC2
CRMP1
FKBP8
SLC25A53
CELF4
ITPK1
NFASC
PPT1
RUFY3
TAGLN3
NISCH
RHBDD2
NAV1
ATP2B1
MAP1B
DPP6
MAPRE3
FGD4
DHRS13
CDK5
GRK2
$-\log_{10}$(Adj P.value)
$\log_2$(Fold change Sorted/Unsorted)

Figure S14

**Fig. S14. Related to Figure 5: Stem cell-derived islet individual response traces. (A)** Individual (grey) and averaged traces (colored) of dynamic insulin secretion measurements in response to glucose (15 or 6 mM), leucine (5 mM), oleate and palmitate (1.5 mM, 1:1 mixture) or KCl (30 mM) in stem cell-derived islet (5 differentiations, GFP-Fluorescence-activated cell (FAC) sorted, stage 6 (days 21-25). Basal glucose was 3 mM. **(B)** Same as (A) except in 8 differentiations of unsorted clusters (stage 6, days 21-30). **(C)** Same as (B) except in 8 differentiations of older-maturing clusters (stage 6, days 55+). **(D)** Volcano plot showing differential protein abundance between GFP-FAC sorted stem cell derived islet like clusters (purple, n=11) and GFP-unsorted clusters (green, n=25). The top 30 most significant differentially abundant proteins are highlighted by labelling with gene name.

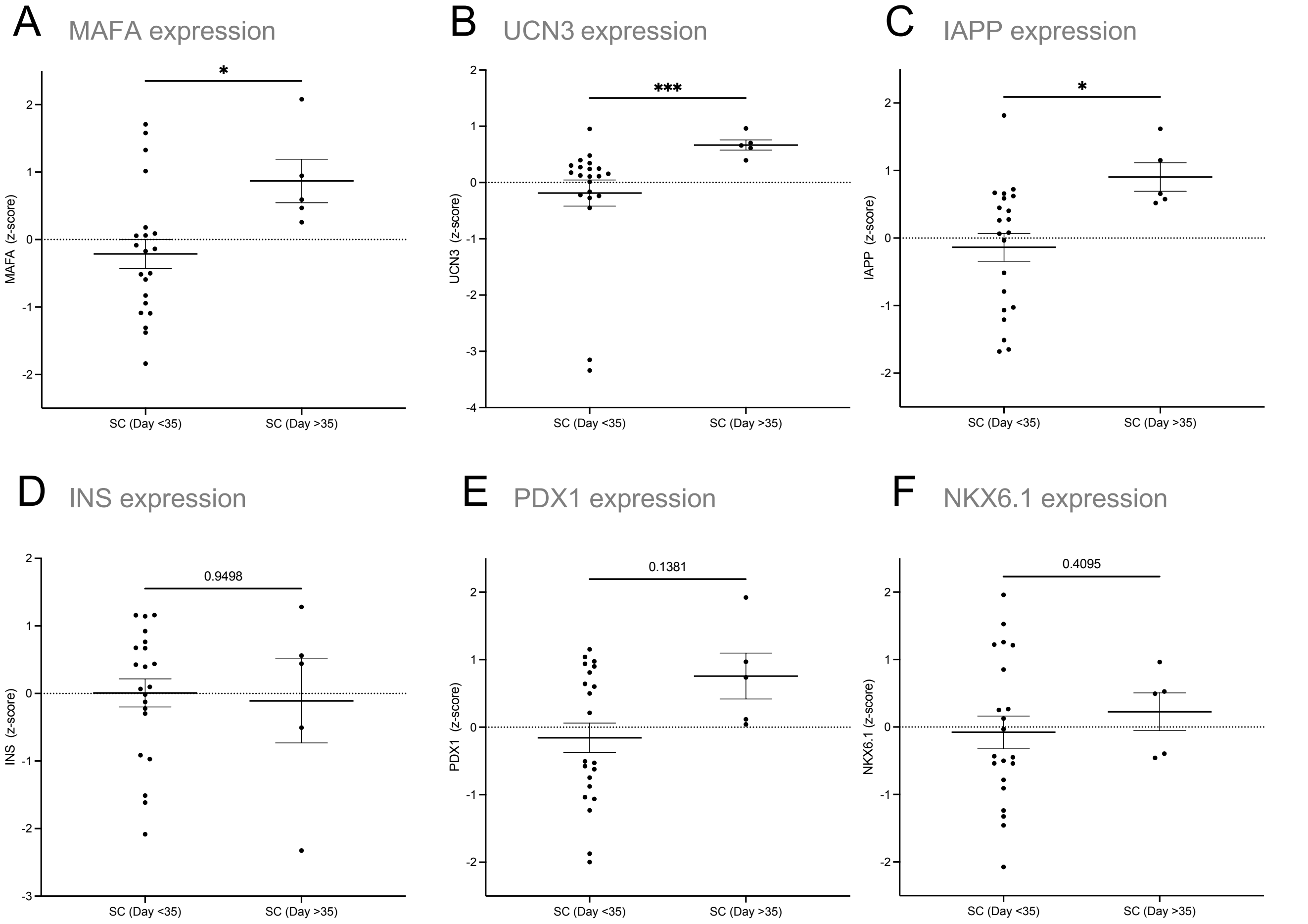


**Fig. S15. Related to Figure 5: Transcriptional maturation of stem cell derived islets.** Calculated z-scores of key maturation transcripts **(A)** MAFA, **(B)** UCN3, **(C)** IAPP, **(D)** INS, **(E)** PDX1, and **(F)** NKX6.1 are compared between stem cell-derived islet clusters less than and greater than 35 days of age. * indicates $p<0.05$, and *** indicates $p<0.001$.
