# Supplementary material for: Proteomic predictors of individualized nutrient-specific insulin secretion in health and disease
Source: Cell Metab. 2024 Jul 2;36(7):1619–1633.e5. doi: 10.1016/j.cmet.2024.06.001 (PMC11250105; doi:10.1016/j.cmet.2024.06.001)
Supplement: Document S2. Article plus supplemental information [file mmc15.pdf]

Cell Metabolism

Resource

Proteomic predictors of individualized nutrient-specific insulin secretion in health and disease

Graphical abstract

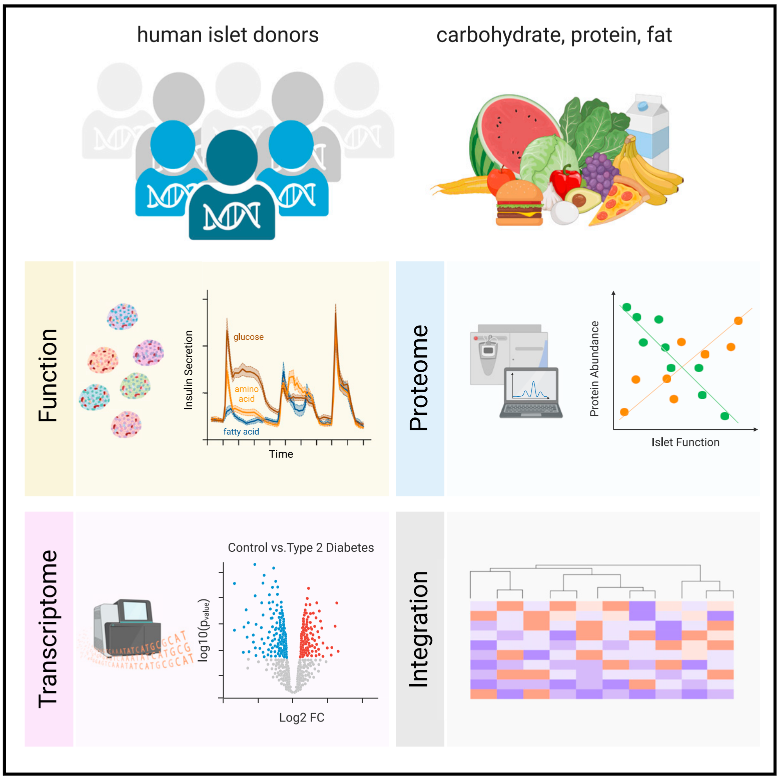

Authors

Jelena Kolic, WenQing Grace Sun, Haoning Howard Cen, ..., Leonard J. Foster, Patrick E. MacDonald, James D. Johnson

Correspondence

jkolic@mail.ubc.ca (J.K.), james.d.johnson@ubc.ca (J.D.J.)

In brief

A comprehensive analysis of insulin secretion dynamics from human islets (with and without type 2 diabetes) exposed to the three main macronutrients—carbohydrate, protein, and fat—complemented with integrated transcriptomic and proteomic analyses reveals the diversity and mechanisms of nutrient responses between 140 individuals.

Highlights

- Insulin secretion to 3 model macronutrients in 140 human islet donors is defined
- Deep phenotyping and multi-omics reveal personalized nutrient-specific insulin release
- A subset of donors with a unique proteome exhibits large insulin responses to lipids
- Nutrient responsiveness in stem cell-derived islets is dysregulated

Kolic et al., 2024, Cell Metabolism *36*, 1619–1633
July 2, 2024 © 2024 The Authors. Published by Elsevier Inc.
https://doi.org/10.1016/j.cmet.2024.06.001

CellPress

Cell Metabolism

CellPress
OPEN ACCESS

Resource

Proteomic predictors of individualized nutrient-specific insulin secretion in health and disease

Jelena Kolic,[1,*] WenQing Grace Sun,[1,13] Haoning Howard Cen,[1,13] Jessica D. Ewald,[2] Jason C. Rogalski,[3] Shugo Sasaki,[4,5] Han Sun,[6] Varsha Rajesh,[6] Yi Han Xia,[1] Renata Moravcova,[3] Søs Skovsø,[7] Aliya F. Spigelman,[8,9] Jocelyn E. Manning Fox,[8,9] James Lyon,[8,9] Leanne Beet,[1] Jianguo Xia,[2] Francis C. Lynn,[4,5] Anna L. Gloyn,[6,10,11] Leonard J. Foster,[3] Patrick E. MacDonald,[8,9] and James D. Johnson[1,12,14,*]

[1]Department of Cellular and Physiological Sciences, Life Sciences Institute, University of British Columbia, Vancouver, BC, Canada
[2]Institute of Parasitology, McGill University, Montreal, QC, Canada
[3]Department of Biochemistry and Molecular Biology, University of British Columbia, Vancouver, BC, Canada
[4]Diabetes Research Group, BC Children's Hospital Research Institute, Vancouver, BC, Canada
[5]Department of Surgery, School of Biomedical Engineering, University of British Columbia, Vancouver, BC, Canada
[6]Department of Pediatrics, Division of Endocrinology, Stanford School of Medicine, Stanford, CA, USA
[7]Valkyrie Life Sciences, Vancouver, BC, Canada
[8]Department of Pharmacology, University of Alberta, Edmonton, AB, Canada
[9]Alberta Diabetes Institute, University of Alberta, Edmonton, AB, Canada
[10]Stanford Diabetes Research Center, Stanford School of Medicine, Stanford, CA, USA
[11]Wellcome Center for Human Genetics, University of Oxford, Oxford, UK
[12]Vancouver Coastal Health Research Institute, Vancouver, BC, Canada
[13]These authors contributed equally
[14]Lead contact
*Correspondence: jkolic@mail.ubc.ca (J.K.), james.d.johnson@ubc.ca (J.D.J.)
https://doi.org/10.1016/j.cmet.2024.06.001

SUMMARY

Population-level variation and mechanisms behind insulin secretion in response to carbohydrate, protein, and fat remain uncharacterized. We defined prototypical insulin secretion responses to three macronutrients in islets from 140 cadaveric donors, including those with type 2 diabetes. The majority of donors' islets exhibited the highest insulin response to glucose, moderate response to amino acid, and minimal response to fatty acid. However, 9% of donors' islets had amino acid responses, and 8% had fatty acid responses that were larger than their glucose-stimulated insulin responses. We leveraged this heterogeneity and used multi-omics to identify molecular correlates of nutrient responsiveness, as well as proteins and mRNAs altered in type 2 diabetes. We also examined nutrient-stimulated insulin release from stem cell-derived islets and observed responsiveness to fat but not carbohydrate or protein—potentially a hallmark of immaturity. Understanding the diversity of insulin responses to carbohydrate, protein, and fat lays the groundwork for personalized nutrition.

INTRODUCTION

Insulin is released by pancreatic islet beta cells in response to nutrient stimuli to maintain energy homeostasis. The major driver of insulin secretion is glucose. However, proteins and fats may also modulate insulin release, and the effects of non-carbohydrate nutrients on insulin secretion remain underexplored. Much of our limited understanding of nutrient-stimulated insulin secretion is extrapolated from rodents, although the more recent availability of cadaveric human islets for research purposes has expanded our pre-clinical knowledge. However, current human islet datasets generally only examine a single nutrient stimulus, glucose.[1–3] A few small studies examined other nutrients,[4,5] but no large-scale direct comparison of insulin secretion in human islets stimulated by carbohydrates, proteins, and fats has been reported. Understanding nutrient-induced insulin secretion is important in the context of type 2 diabetes (T2D) and emerging studies linking hyperinsulinemia with a range of maladies, including cancer.[6] Indeed, large prospective clinical trials show broad beneficial effects of diets targeting hyperinsulinemia.[7]

Individuals respond differently to diets,[8] and there is high interpersonal variability in postprandial responses to even one macronutrient, glucose.[9] *Ex vivo studies* measuring insulin secretion from human islets show high variability, only some of which can be explained by donor characteristics or islet isolation parameters.[2] The concept that insulin release from islets is

This is an open access article under the CC BY license (http://creativecommons.org/licenses/by/4.0/).

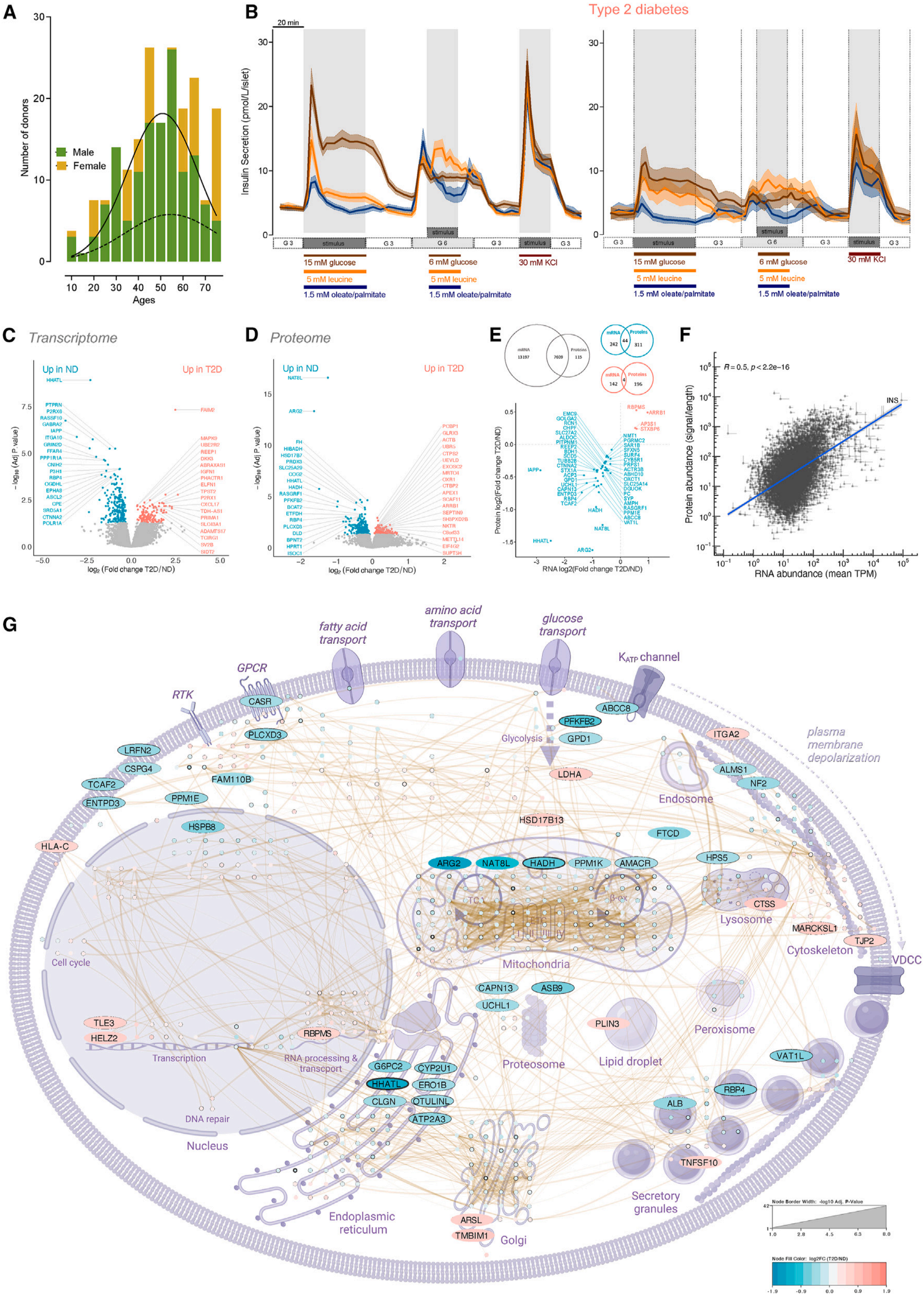

Figure 1. Nutrient-stimulated insulin secretion, transcriptomes, and proteomes from ND and T2D islets

(A) Histogram with trend line of ages for male (green, solid line) and female (yellow, dashed line) donors. Additional details of islet isolation parameters and donor characteristics are available in Table S1 and on www.humanislets.com.

(legend continued on next page)

individualized in response to food types or different macronutrients has not been investigated mechanistically; no study has leveraged macronutrient-induced insulin secretion heterogeneity and large-scale multi-omics to elucidate associated molecular mechanisms.

Here we addressed these critical knowledge gaps by measuring dynamic insulin secretion in response to three model macronutrient stimuli in islets from non-diabetic (ND) donors and donors with T2D, as well as stem cell-derived islets. Our transcriptomic and proteomic analysis revealed molecular signatures of each donor's islets and identified distinct clusters of proteins that predict insulin secretion responses to carbohydrates, proteins, and fats. We also report on a subset of individual's islets that are relatively hyper-responsive to lipids in a way that resembles functionally immature human embryonic stem cell-derived islets. This is the largest human islet dataset that includes macronutrient-stimulated insulin secretion measurements and multi-omic profiling, coupled with the first side-by-side comparison of nutrient responses and proteomes between human islets and stem cell-derived islets. This resource helps us understand why individuals' islets respond differently to sugar, protein, and fat and advocates for greater application of personalized recommendations and treatments for individuals living with diabetes.

RESULTS

Prototypical nutrient-stimulated insulin secretion dynamics from human islets

Between 2016 and 2022, we systematically measured insulin secretion in response to carbohydrate (15 or 6 mM glucose), amino acid (5 mM leucine), and fatty acids (1.5 mM oleate/palmitate mix) from islets isolated from 140 cadaveric donors[2] reflective of the general population (Figure 1A).[10] In most islet donors, we confirmed that carbohydrate was the strongest insulin secretagogue, followed by amino acid and then fatty acids, which only weakly stimulated insulin secretion on average (Figures 1B, S1A, and S1B). Islets exhibited biphasic insulin secretion in response to glucose.[11] We found that insulin secretion in response to amino acid is also biphasic, with a distinct 1st phase lasting ~15 min and a sustained 2nd phase lasting the duration of the challenge (Figures 1B, S1A, and S1B). In contrast, the response to fatty acid, when present, was monophasic (Figures 1B, S1A, and S1B).

Our experimental design also gave us the opportunity to assess the role of macronutrient order on insulin secretion, inspired by clinical meal-order studies.[12,13] In our experiments, prior exposure to high glucose, amino acid, or fatty acid did not alter insulin secretion stimulated by moderately elevated 6 mM glucose (Figures S1A and S1B). Amino acid on top of 6 mM glucose further increased insulin secretion (Figure S1A). Fatty acid response was not enhanced in the presence of 6 mM glucose (Figures S1A and S1B), in contrast to previous small-scale rodent or human islet observations.[14,15] Interestingly, insulin secretion stimulated by direct depolarization with KCl was inhibited after prior exposure to lipid in islets from ND donors (Figure S1A), perhaps foreshadowing lipotoxic effects on the insulin secretory machinery.[16] This large dataset provides the first side-by-side response profiles for each of the three main macronutrients in human islets.

Prototypical nutrient-stimulated insulin secretion dynamics in type 2 diabetes

We next examined the relationship between T2D and nutrient-stimulated insulin secretion. When focusing on the kinetics of insulin secretion, we saw a significant delay in time-to-insulin peak in response to high glucose in islets from donors with family-reported T2D (Table S1; Figures S1C–S1E). Islets from donors with T2D had ~40% lower insulin secretion in response to 15 mM glucose and ~35% lower insulin secretion in response to moderate 6 mM glucose (Figures 1B, S2A, and S2B). Insulin secretion in response to direct depolarization by KCl was reduced by ~22% but failed to reach statistical significance (Figure S2C, $p = 0.07$). Insulin secretion in response to fat was lowered by ~55% (Figures 1B, S2D, and S2E). However, insulin content, baseline insulin secretion, and insulin secretion in response to leucine were not statistically different (Figures 1B and S2F–S2H). This preserved amino acid-stimulated insulin secretion is consistent with clinical data[17] and suggests that therapeutic protein intake could be exploited in diabetes management. However, leucine together with 6 mM glucose induced ~36% (on average) less insulin secretion in donors with diabetes (Figure S2I), and leucine was not stimulatory on top of 6 mM glucose in these donors (Figure S1B), emphasizing a need for additional clinical research into context-dependent amino acid-stimulated insulin secretion. Known glucose-lowering medication status of these donors with diabetes did not significantly correlate with any insulin-secretion parameters; however, there was an overall trend with the need for exogenous insulin and lower overall insulin secretory capacity (Figures S2J–S2Q).

Comprehensive transcriptomics and proteomics of islets with and without type 2 diabetes

To better understand the relationship between secretory response and variation in gene expression in islets from donors

(B) Averaged traces of dynamic insulin secretion measurements in response to glucose (15 or 6 mM), leucine (5 mM), oleate and palmitate (1.5 mM, 1:1 mixture), or KCl (30 mM) in islets isolated from ND donors ($n = 123$) (left) and donors with T2D ($n = 17$) (right) are shown as indicated. Basal glucose was 3 mM. Error bars (SEM) are depicted by continuous shaded regions around the corresponding solid lines.

(C) RNA-seq identified enriched mRNAs in islets from ND donors ($n = 82$), shown in teal, and enriched mRNAs in islets from donors with T2D ($n = 8$), shown in red. The top 40 significant differentially expressed transcripts are labeled with their gene names.

(D) Mass-spectrometry-based proteomics identified enriched proteins in ND ($n = 118$) in teal and T2D ($n = 16$) in red. The top 40 significant differentially abundant proteins are highlighted by labeling with their gene names.

(E) (Top) Venn diagrams show the overlap of differentially expressed mRNAs and abundant proteins, further depicted in the correlation plot (bottom).

(F) Across-gene correlation between mRNAs and proteins.

(G) Differentially abundant proteins (with a greater than 0.5 $\log_2$ fold change) between the ND and T2D donor islets were connected in a protein-protein interaction network (gold lines) using STRING and depicted in the context of their beta cell compartments and functions. The color of the nodes represents the fold change (ND/T2D), while the thickness of the line around the nodes represents the p value (adjusted).

with and without T2D, we performed comprehensive transcriptomic and proteomic analysis from the majority of the phenotyped batches of donor islets. On average, after quality control, we measured >20,000 mRNAs using RNA sequencing (RNA-seq) (from 82 ND and 8 T2D donors) and ~8,000 proteins (from 118 ND and 16 T2D donors) using mass-spectrometry. We used our multi-omic dataset to estimate islet cell composition in our donor preps based on abundance of proteins typically enriched in islet and acinar cells.[18] This model indicates that isolated islets from donors with T2D have altered islet cell composition, with lower beta cell and higher alpha cell markers, as well as a higher percentage of acinar cell markers (Figure S3).

RNA-seq of a large subset of human islets showed that 286 mRNAs were enriched in the ND donors and 146 were enriched in the donors with diabetes (Figure 1C; Table S2). Importantly, differences were more apparent at the protein level, with 355 proteins significantly more abundant in islets from ND donors and 200 proteins more abundant in islets from donors with diabetes (Figure 1D; Table S3). Despite only finding 48 gene products that were significantly altered at both mRNA and protein levels (Figure 1E), individual protein fold changes were mostly consistent with mRNA fold changes (Figure S4A), and both lists of features were enriched with many of the same pathways (Figure S4B).

Gene products decreased in both expression and abundance in islets from donors with diabetes included the sulfonylurea receptor subunit of the K_{ATP} channel (*ABCC8*), the well-known target of oral hypoglycemic agents, as well as pyruvate carboxylase (*PC*), which is critical for mitochondrial metabolism and glucose-stimulated insulin release,[19] hedgehog acyltransferase-like (*HHATL*), which negatively regulates protein palmitoylation, a process implicated in T2D[20], and islet amyloid polypeptide (*IAPP*), a hormone co-secreted with insulin with roles in glycemic control and gastric emptying[21] (Figure 1E). Only four gene products were increased in both expression and abundance in T2D: RNA-binding protein with multiple splicing (RBPMS), AP-3 complex subunit sigma-1 (AP3S1), which is involved in lysosomal trafficking,[22] syntaxin binding protein 6 (*STXBP6*), which is thought to limit insulin release by limiting the size of the granule fusion pore,[23] and beta arrestin 1(*ARRB1*), which is suggested to be involved in glucagon-like peptide-1 (GLP-1) stimulated insulin secretion.[24]

The overall discordance between islet RNA expression and protein abundance (7,609 gene products) from the same donor/isolations had an R value of 0.5 (Figure 1F), consistent with other studies of primary tissues.[25,26] Proteins had a lower coefficient of variation than transcripts (Figure S4C). As such, we did not detect significant RNA-protein correlations for 81% of the gene products (Figures S4D–S4F). This discrepancy was likely not due to unreliable RNA-seq measurements because we observed a strong correlation between the RNA-seq and NanoString analysis of 130 mRNAs (Figures S4G and S4H). We also used an alternative approach to compare the protein and RNA changes in T2D by utilizing the rank-rank hypergeometric overlap test,[27] which identified ~3,300 proteins and RNAs that were changed in the same direction in T2D (Figures S5A–S5C). However, we found that this method was not stringent enough for our purposes since the vast majority of the overlapping proteins and RNAs identified had negligible fold change difference in T2D (Figure S5).

Because proteins had lower coefficients of variation (Figure S4), were more stable in the face of isolation variables (see below), and may provide better insight into disease phenotype than mRNAs,[26] we focused on the proteomic data to elucidate key mechanisms. We mapped the protein-protein interaction networks for proteins showing fold change greater than 0.5 $\log_2$ using the Search Tool for the Retrieval of Interacting Genes/Proteins (STRING) database,[28] and subsequently positioned them in a diagram using subcellular location information found in UniProt and using the literature (Figure 1G). Consistent with a lower insulin secretory response to stimulatory glucose (Figures S2A and S2B), islets from donors with T2D had lower abundance of key proteins predicted to be involved in glucose-stimulated insulin secretion. Islet proteins reduced in T2D (Figure 1G) included mitochondrial proteins arginase 2 (ARG2), N-acetylaspartate synthetase (NAT8L), hydroxyacyl-coenzyme A dehydrogenase, mitochondrial (HADH), protein phosphatase 1K, mitochondrial (PPM1K), and alpha-methylacyl-CoA racemase (AMACR), regulators of glycolysis (6-phosphofructo-2-kinase/fructose-2,6-bisphosphatase 2 (PFKFB2) and glucose-6-phosphatase 2 (G6PC2), endoplasmic reticulum Ca^{2+} homeostasis factors ERO1-like protein beta (ERO1B), inactive ubiquitin thioesterase (OTULINL), sarcoplasmic/endoplasmic reticulum calcium ATPase 3 (ATP2A3), and the extracellular Ca^{2+}-sensing receptor (CASR). Proteins that were more abundant in islets from donors with diabetes included those crucial for cell adhesion: tight junction protein ZO-2 (TJP2), integrin alpha-2/beta-1 (ITGA2), Golgi function arylsulfatase L (ARSL), transmembrane Bcl-associated X-protein (BAX) inhibitor motif containing 1 (TMBIM1), transcriptional proteins such as TLE family member 3 (TLE3), helicase with zinc finger domain 2 (HELZ2) and RNA-binding protein with multiple splicing (RBPMS), and proteins involved in cytoskeletal reorganization like myristoylated alanine-rich C-kinase (MARCKS)-related protein (MARCKSK1). Collectively, these data identify multiple proteins that correlate with the insufficient glucose-stimulated insulin secretion in islets from donors with diabetes.

Individuality of nutrient-stimulated insulin secretion

Islets from individual donors exhibited a large range of insulin secretion rates at baseline and in response to high glucose, moderate glucose, amino acid, fat, and direct depolarization (Figure 2A). Surprisingly, we observed that some donors had more robust responses to fatty acids than to glucose, challenging the long-standing idea that dietary fats alone have negligible effects on insulin release.[5,14,29] This degree of response heterogeneity across all macronutrients was not found in C57Bl6J mouse islets, even when including both sexes and a wide range of ages (Figure 2B).

We explored the source of this variation by examining known donor characteristics (Table S1) and islet isolation parameters. Cold ischemic time of the pancreas was negatively correlated with insulin secretion in response to direct depolarization (Figure S6A), but it was not correlated to insulin secretion stimulated by any of the three macronutrients tested. We also found that islets from female donors had lower insulin secretion at 3 mM glucose, 6 mM glucose, and 6 mM glucose with fatty acids (Figures S6B–S6D). Donor age and BMI were not different between male and female donors in our study (Table S1), suggesting that these differences are a result of biological sex. Somewhat

CellPress
OPEN ACCESS

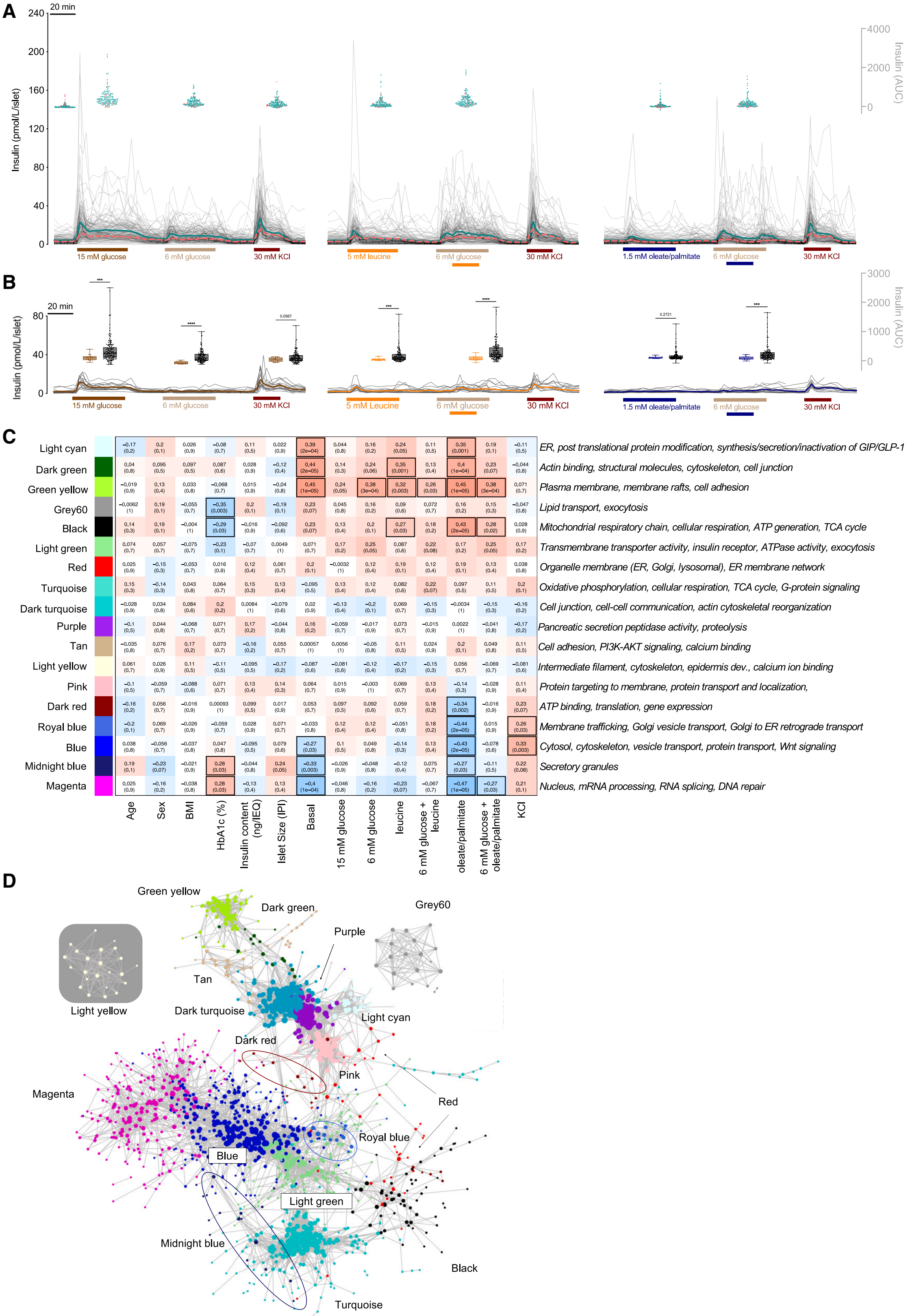

(legend on next page)

surprisingly, donor BMI had only minor effects on macronutrient-stimulated insulin secretion (Figures S6E–S6M). Expectedly, high HbA1c was negatively correlated to insulin secretion stimulated by high glucose and KCl (Figures S6N–S6P). These observations suggest that donor characteristics and islet isolation parameters minimally contribute to individualized nutrient responses and that other factors likely drive the heterogeneity we observed.

Co-expression network analysis uncovers protein networks correlated with islet function

We used co-expression network analysis to obtain an overview of protein-protein relationships within the proteome (and transcriptome) and then analyzed the network along with clinical and functional outcomes to gain insight into the molecular drivers of macronutrient-stimulated insulin secretion response heterogeneity. We identified 18 network modules of highly co-regulated proteins (Figures 2C and S7) and plotted them as a co-expression network to illustrate the connections between modules (Figure 2D). All modules were significantly enriched in gene sets (Gene Ontology cellular component, molecular function, and biological process) and pathways (Kyoto Encyclopedia of Genes and Genomes [KEGG] and Reactome), allowing for their higher-level functional annotation. The overall protein abundances of 9 modules were significantly correlated with islet functional data (Figure 2C). Several modules were positively correlated with insulin secretion in response to multiple nutrients. These modules (light cyan, dark green, green yellow, and black) contained proteins with critical roles in cytoskeletal reorganization, mitochondrial metabolism, and insulin processing. Two modules of interest (blue and royal blue) with roles in protein transport and localization were positively correlated with insulin secretion stimulated by KCl but negatively correlated with insulin secretion stimulated by lipids. Notable proteins in this module include enzymes involved in fatty acid synthesis: acetyl-CoA carboxylase 1 (ACACA) and fatty acid synthase (FASN),[30,31] and lipid metabolism, glycerol-3-phosphate dehydrogenase (GPD1).[32] Consistent with the discordance between RNA expression and protein abundance, and suggestive of more dynamic mRNA levels, we did not observe any significantly correlated transcript modules in the 29 network modules detected (Figure S8). Together, our analysis suggests that these protein network modules in human islets underpin their differential responsiveness to stimuli.

Associations between individual proteins, donor data, and islet function

We next correlated the abundance of each mRNA (Figure S9; Table S4) and protein (Figure S10; Table S5) identified in our dataset to donor traits, independent of diabetes status. However, some of these correlations were driven by disease status rather than islet function (Table S6), so we reanalyzed our dataset using a linear regression model to adjust for T2D diagnosis (Figures 3 and S11). We focused our attention on the proteome because we found that mRNAs were poorly correlated with islet function (Figures S9 and S11) and were more prominently correlated with islet culture and isolation variables (Figure S12; Tables S6, S7, and S8).

Our analysis suggests that increasing donor age was positively associated to proteins with known and postulated roles in lysosomal degradation, cellular senescence, inflammation, and mitochondrial dysfunction: palmitoyl-protein thioesterase 1 (PPT1)[33] scavenger receptor class B member 2 (SCARB2),[34] acid ceramidase (ASAH1),[35] purinergic receptor P2X4,[36] and nicotinamide adenine dinucleotide phosphate (NADPH):adrenodoxin oxidoreductase (FDXR)[37] (Figure 3A; Table S8). We found that the abundances of 161 proteins were associated with HbA1c (Figure 3B). Interestingly, the most significant positive relationships were seen to proteins with known roles in cancer progression and development: protein YIF1A,[38] ubiquitin-associated protein 1 (UBAP1),[39] DDRGK domain-containing protein 1 (DDRGK1),[40] and syntaxin-5 (STX5).[41] Poor glycemic control is associated with increased risk of multiple cancers.[42]

We also looked for relationships between protein abundance and islet function in response to each nutrient stimulus (Figures 3C–3I). The greatest number of significant associations to protein abundance were seen with insulin secretion induced by lipids (2,053 significant associations) (Figure 3G; Tables S6 and S8), pointing to somewhat unique mechanisms for this macronutrient in insulin release. The abundance of 3 proteins was positively associated to insulin secretion stimulated by all three macronutrients as well as direct depolarization by KCl (Figures 3J and 3K), suggesting that these proteins play a role in the overall secretory capacity of the beta cell. These pan-stimulus-enabling proteins were ADP-ribosylation factor GTPase 6 interacting protein 6 (ARL6IP6), solute carrier family 25 member 4 (SLC25A4), and secretory carrier membrane protein 4 (SCAMP4). These findings highlight the critical role of mitochondria in nutrient- and depolarization-stimulated insulin secretion.[43]

Figure 2. Clustering heterogeneous insulin responses to macronutrients and donor proteomes

(A) (Left) Individual traces of dynamic insulin secretion stimulated by glucose (15 or 6 mM) or KCl (30 mM). Basal glucose was 3 mM. Average responses from ND donors are illustrated with solid teal line, and average responses from donors with T2D are shown in the dashed salmon line. Floating dot plot inserts illustrate the heterogeneity in insulin AUC for the corresponding section of the perfusion curve (salmon dots illustrate the responses from donors with T2D). Middle panel shows islets stimulated with leucine (5 mM) alone or in combination with 6 mM glucose, as indicated. Right shows islets stimulated with oleate/palmitate (1.5 mM, 1:1 mix) alone or in combination with 6 mM glucose, as indicated.

(B) Illustrates the same as (A) except in mouse islets (8 males, 9 females, 7–90 weeks of age). Floating box plots illustrate the variance of AUC responses between the mouse islet (brown, orange, or blue) and human islet (gray) responses. *** indicates $p < 0.001$ and **** indicates $p < 0.0001$.

(C) Co-correlation analysis identified 18 distinct modules of proteins whose abundance shows similar patterns. Modules are annotated with KEGG pathways and GO terms. The Pearson correlation coefficients between the modules and the donor metadata or functional data (x axis) are shown in each rectangle, and the corresponding adjusted p value is shown in the brackets. Positive correlations are shown in shades of red and negative in shades of blue. Significant correlations are highlighted with black box outlines.

(D) Illustrates the main connections in the co-expression network. The WGCNA adjacency matrix was filtered to remove: (1) any protein not in a module, (2) protein-protein adjacency distances less than 0.2, and (3) any protein without any connections after filters 1 and 2. The resulting network contained 1,600 protein nodes and 16,209 edges. Each protein node is colored according to its module (same color scheme as C).

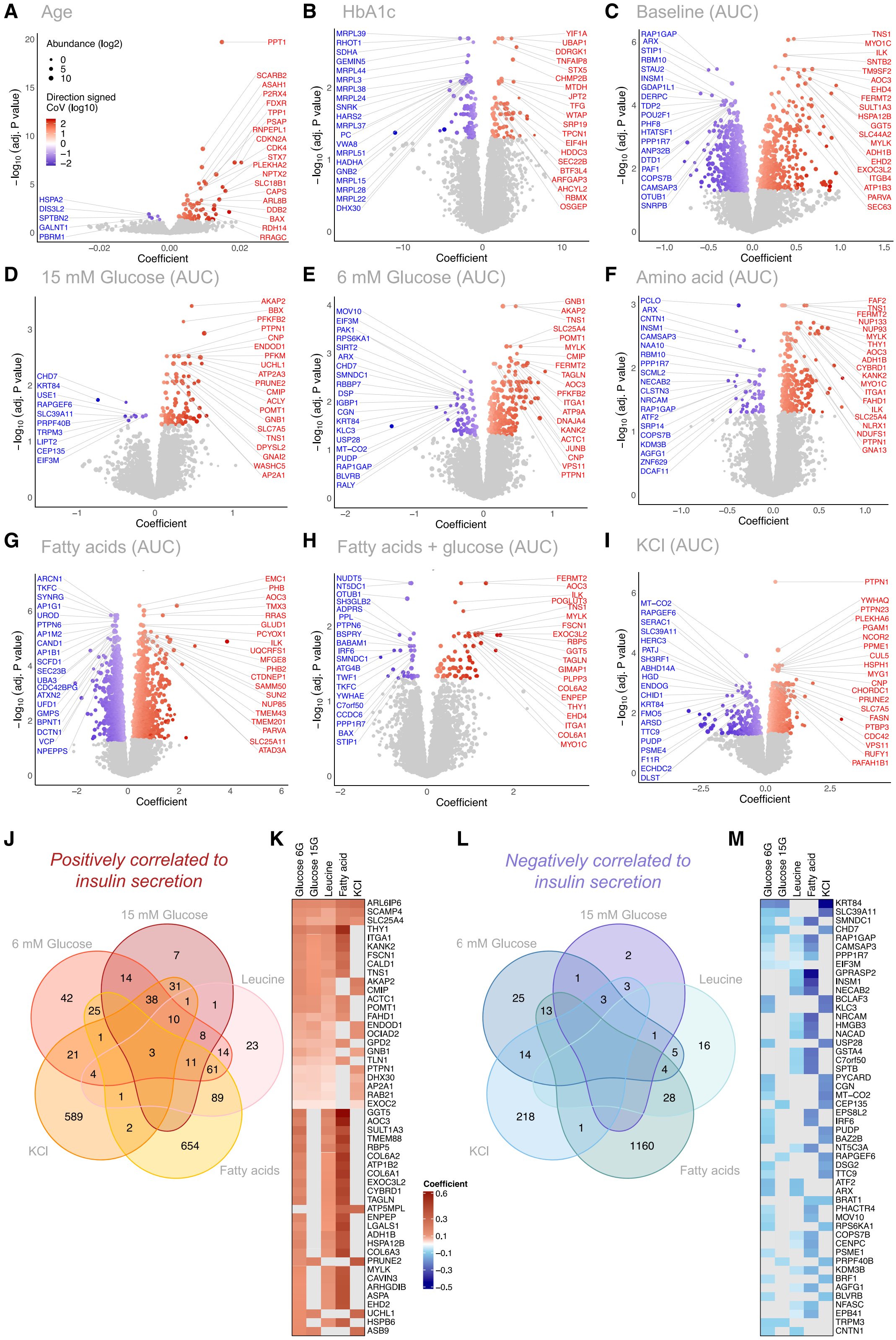

Figure 3. Regression coefficients of individual proteins with insulin secretory responses

(A–I) Volcano plots are shown depicting significant positive (red) and negative (blue) linear regression coefficients of proteins to continuous donor characteristics: (A) donor age and (B) HbA1c; or functional parameters in response to (C) 3 mM glucose, (D) 15 mM glucose, (E) 6 mM glucose, (F) 5 mM leucine, (G) 1.5 mM

(legend continued on next page)

Alpha cell transcription factor aristaless related homeobox (ARX)[44] was negatively associated with basal insulin secretion (Figure 3C), insulin secretion stimulated by glucose (Figure 3E), as well as insulin secretion stimulated by amino acid (Figure 3F) (Table S8). While these correlations may initially suggest a general decrease in beta cell ability to secrete insulin on account of higher alpha cell mass, we also observe that insulin secretion induced by direct depolarization with KCl was not correlated to ARX abundance (Figures 3L and 3M; Table S8), arguing against such a simple interpretation. Collectively, these data provide unprecedented information on the proteins that associate with islet secretory function in a general and nutrient-specific way. Our team has built an online resource where the relationship between any detected protein, donor characteristics, islet isolation parameters, and islet function can be analyzed and displayed (www.humanislets.com).

Unique proteomes of lipid or amino acid hyper-responders

During the course of our multi-nutrient phenotyping studies, we identified a previously unreported sub-group of donors with an unusually large insulin secretory response to lipids in basal glucose conditions. In fact, ~8% of donors secreted more insulin in response to oleate/palmitate than to 15 mM glucose (Figures 4A and 4B). These donors also secreted more insulin when lipid was present together with 6 mM glucose but less insulin in response to direct depolarization by KCl (Figure 4A; Table S9). Overall, lipid-hyper-responsive islets came from donors with higher HbA1c, but there were no other significant differences in islet isolation or culture parameters seen between the two groups (Table S9).

However, proteomic comparison of these two donor groups showed that the fat-hyper-responsive islets exhibited a 30% decrease in the abundance of Wnt family member 4 (WNT4), hinting at a less-mature state[45] (Figure 4C). Fat-hyper-responsive islets also had less protein phosphatase 1 catalytic subunit gamma (PPP1CC), which is involved in glycogen metabolism; hypoxanthine phosphoribosyltransferase 1 (HPRT1), which is involved in purine metabolism; and AKT serine/threonine kinase 1 (AKT1), which is involved in growth factor signaling and survival. On the other hand, lipid hyper-responders exhibited elevated hepatocyte nuclear factor 1-beta (HNF1B), the gene product underlying a rare form of diabetes, maturity onset diabetes of the young (MODY5), sulfotransferase family 1A member 1 (SULT1A1), and platelet-derived growth factor receptor alpha (PDGFRA) (Figure 4C). Pathway analysis using multiple databases[46] reveals defects in small-molecule protein modification, AKT signaling, and lipid metabolism in donors classified as fat hyper-responders (Figure S13A). Interestingly, proteins involved in endoplasmic reticulum signal integration and organization are increased in fat hyper-responders (Figure S13B), which is perhaps suggestive of endoplasmic reticulum dysfunction in these donors.[47]

We also identified a previously unreported subset of donors (~9%) that secreted more insulin in response to leucine than to high glucose (Figures 4D and 4E; Table S10). Amino acid hyper-responders were more likely to been diagnosed with T2D, but there was no difference in HbA1c between the high and low responders (Table S10). Amino acid hyper-response was also associated with a longer *in vitro* culture time (Table S10), suggesting that this observed phenotype may be influenced by adaptation to our culture conditions. Only two differentially abundant proteins (Figure 4F) were associated with this phenotype, thymocyte selection-associated high mobility group box protein (TOX; transcriptional regulator) and angiotensinogen (AGT), an essential component in the renin-angiotensin system, with important roles in the endocrine pancreas.[48] Our work now suggests that some donors' islet proteomes may be pre-programmed or may have adapted to *in vitro* culture conditions, which enables them to hyper-respond to lipids and/or amino acids.

Stem cell-derived islet-like clusters are lipid responsive

The differentiation of beta cell surrogates from embryonic stem cells has potential as a diabetes therapy and can be used to model "environmentally naive" insulin secretion.[49] The *in vitro* insulin-secretory function of these cells, even in response to supraphysiological levels of glucose, still does not match that of human islets.[49] There are no reports that directly compare how stem cell-derived islets and primary human islets respond to multiple nutrients[49]; however, recent work has suggested that human mitochondria from pluripotent stem cell-derived islets have a higher sensitivity for non-glucose fuels.[50]

In our hands, INS-2A-GFP stem cell-derived islet-like clusters[51] (Figures 5A and 5B) exhibited lower levels of typical beta cell-enriched markers (Figures S3C and S3D) and had ~1/10 the level of basal insulin release when compared with human islets (Figure 5C). They showed poor responsiveness to glucose, were unresponsive to amino acid (either alone or in combination with glucose), but did release significant amounts of insulin upon direct depolarization by KCl (Figures 5C and S14). Notably, the fatty-acid stimulated insulin response magnitude and pattern was similar between stem cell-derived islets and human islets on average. This suggests that these *in vitro*-derived cells have the capacity for regulated insulin secretion but have specific defects in glucose and amino acid responses and may prefer non-glucose substrates.[50]

Comparing their proteomes to human islets revealed massive differences; 3,196 proteins were significantly more abundant in the ND human donor islets (Figures 5D and 5E; Table S3). Some differences were due to the presence of non-islet exocrine cells trapped in our hand-picked preparations (Figures 5E and S3). Other proteomic differences were more informative. WNT4, which we found was less abundant in lipid hyper-responders, was also more than 2 times lower in these hESC-derived islet-like clusters than in islets from ND donors

oleate/palmitate (1:1 mixture), (H) 1.5 mM oleate/palmitate + 6 mM glucose, and (I) 30 mM KCl. Protein abundances (log_2) are depicted by size of circle, and the coefficient of variation (log_{10}) is depicted by color gradient.

(J) Venn diagram showing the overlap of the number of protein abundances that positively associate with the indicated nutrient stimuli following adjustment for T2D disease status diagnosis.

(K) Heat map depicts the top 50 most positively associated proteins to the indicated stimuli.

(L and M) Shows the same as (J) and (K), but for negative associations.

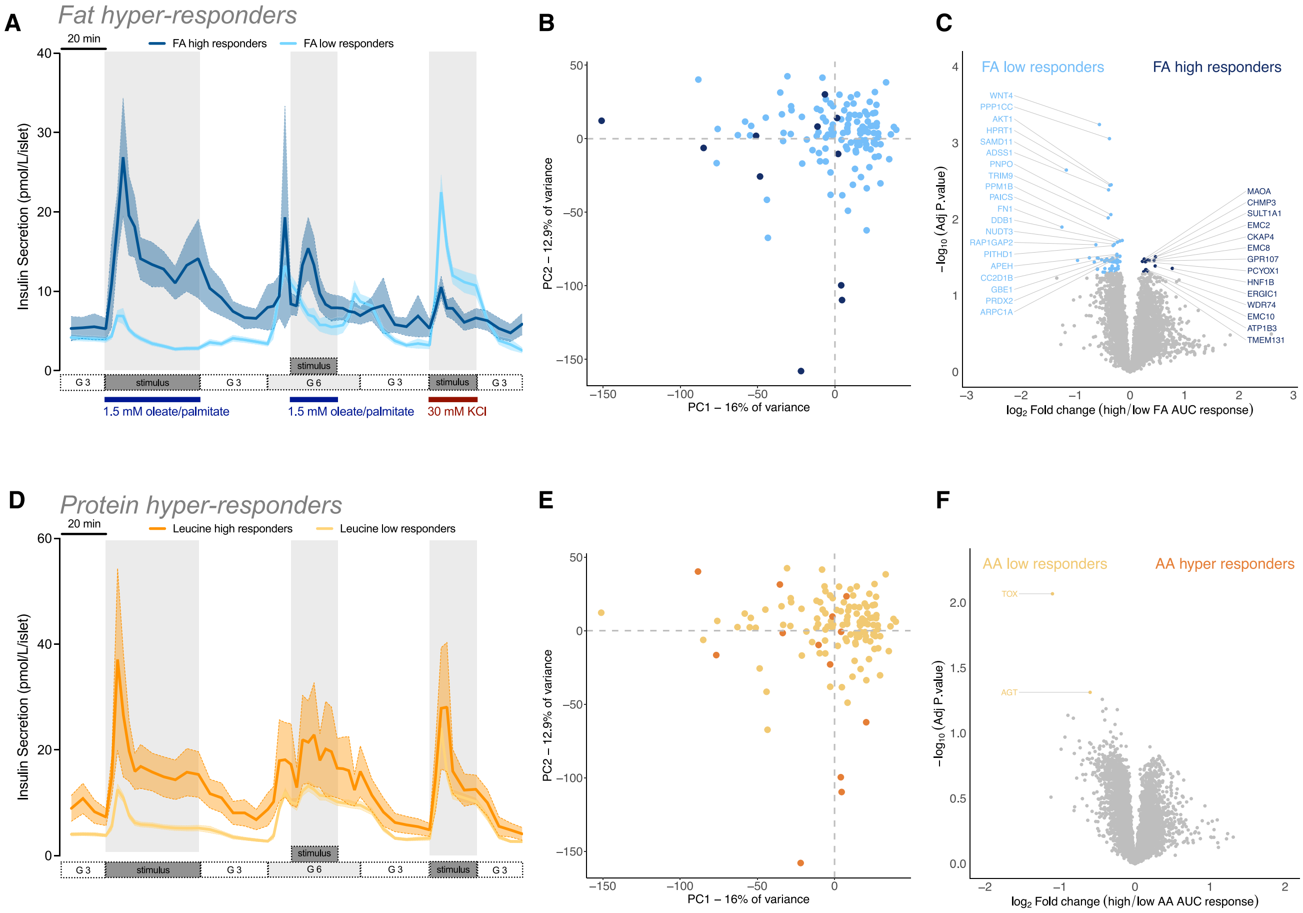

Figure 4. Prototypical responses and proteomic profiles of fat and protein hyper-responders

(A) The average insulin secretion response from donors classified as fatty acid high responders is shown in dark blue (*n* = 11), and the average insulin secretion response from donors classified as "low responders" is shown in the light blue (*n* = 129). Error bars (SEM) are depicted by continuous shaded regions around the corresponding solid lines.

(B) Principal component analysis (PCA) plot of protein abundances in the high (dark blue circles) vs. low (light blue circles) fatty acid responders.

(C) Volcano plot showing differential protein abundances between high and low fatty acid responders.

(D) The average insulin secretion response from donors classified as "high protein responders" is shown in orange (*n* = 13), and the average insulin secretion response from donors classified as "low protein responders" is shown in yellow (*n* = 127). Error bars (SEM) are depicted by continous shaded regions around the corresponding solid lines.

(E) PCA plot of protein abundances in the high (orange circles) vs. low (yellow circles) protein responders.

(F) Volcano plot showing differential protein abundances between high and low protein responders.

(Table S3). On the other hand, 2,553 proteins were more abundant in the stem cell-derived islet clusters (Table S3). Higher levels of this protein in the pancreatic beta cell are thought to limit voltage-dependent calcium channel activity and thus decrease glucose-stimulated insulin secretion.[52] The abundance of monocarboxylate transporter 1 (SLC16A1), a beta cell disallowed gene whose presence is suggestive of a neonatal-like immature beta cell phenotype,[53] increased over 6-fold in these islet-like clusters. Compellingly, proteins linked to important roles in fatty acid transport and metabolism—long-chain fatty acid transport protein 3 (SLC27A3), mitochondrial coenzyme A transporter (SLC25A42), and solute carrier family 22 member 18 (SLC22A18)—were increased ∼2-fold.

Extended maturation of stem cell-derived islet-like clusters increases the ability of these cells to respond appropriately to a glucose stimulus.[54,55] We compared nutrient-stimulated insulin secretion from younger-immature (days 21–35) and older-maturing (days 50+) stem cell preparations (Figures 5F and S14). NanoString analysis showed increased expression of key maturity markers (MAFA, UCN3, and IAPP) in cells older than day 35 (Figure S15). Basal insulin secretion was 5-fold higher in the older-maturing islet-like clusters, and they also showed evidence of glucose responsiveness and maintained their responsiveness to fatty acids (Figure S14). At the level of the proteome, 2,072 proteins were more abundant in the older-maturing clusters and 2,066 more abundant in the younger-immature clusters (Figures 5G and 5H; Table S3). Proteins with significant roles in glucose-stimulated insulin secretion and insulin synthesis, including pyruvate carboxylase (PC), mitochondrial citrate carrier (SLC25A1), high-voltage gated calcium channel subunit (CACNA2D2), glucose

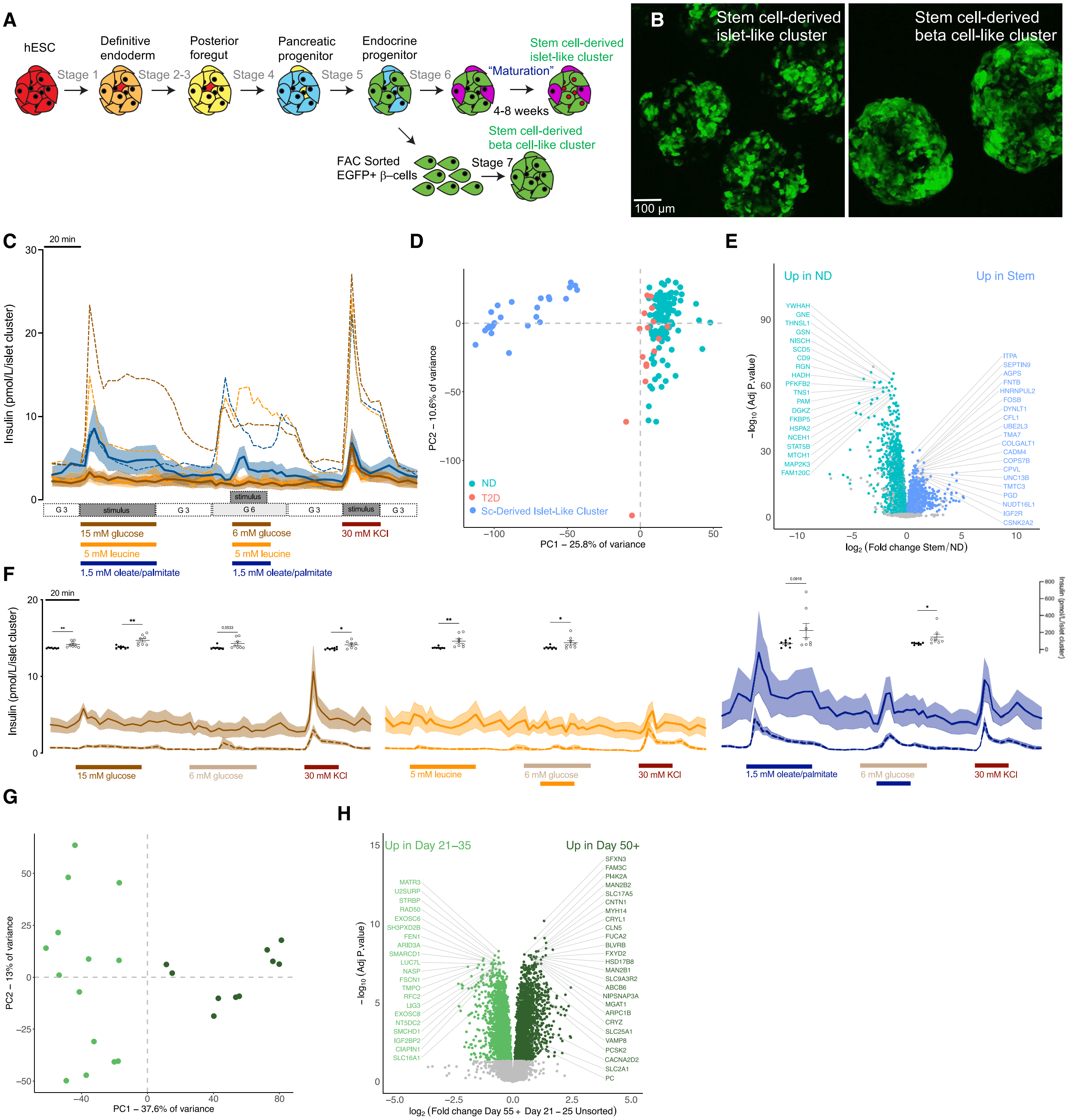

Figure 5. Nutrient responses and proteomic profiles of stem cell-derived islet-like clusters

(A) Summary of the human embryonic stem cell-derived islet-like clusters differentiation protocol.

(B) Representative images of unsorted (day 35) stem cell-derived islet-like clusters (left) and FAC-sorted (day 35) stem cell-derived beta cell-like clusters (right).

(C) Averaged traces of dynamic insulin secretion measurements in response to glucose (15 or 6 mM), leucine (5 mM), oleate and palmitate (1.5 mM, 1:1 mixture), or KCl (30 mM) in stem cell-derived islet-like clusters (average of 8 younger-immature and 8 older-maturing preparations). Basal glucose was 3 mM. For comparison, the average human islet dynamic insulin secretion measurement traces are shown in the dashed lines. Error bars (SEM) are depicted by continous shaded regions around the corresponding solid lines.

(D) PCA plot of protein abundances in the stem cell-derived islet-like clusters ($n = 25$) (blue circles) compared with donors with T2D (salmon circles, $n = 16$) and ND donors (teal circles) ($n = 118$).

(E) Volcano plot showing differential protein abundances between stem cell-derived islet-like clusters (blue circles, $n = 25$) and ND donors (teal circles, $n = 118$). The top 40 most significant differentially abundant proteins are highlighted by labeling with gene name.

(legend continued on next page)

transporter 1 (SLC2A1), an integral SNARE protein (VAMP8), and prohormone convertase 2 (PCSK2), were all significantly more abundant in the older-maturing stem cell-derived islet-like clusters. These cells also had a 73% decrease in the abundance of the beta cell disallowed gene monocarboxylate transporter (SLC16A1), when compared with more immature clusters. These proteomic data now provide a roadmap to produce more faithful human islet surrogates. Although the maturation of stem cell-derived islets remains incomplete *in vitro*,[55,56] and current cell replacement tend to focus on beta-like cells, our dataset can now be used to make better stem cell-derived islets by exploiting the differences seen at the protein levels between these cells and real human islets.

DISCUSSION

The goal of this study was to compare dynamic insulin secretion responses to carbohydrate, protein, and fat from human islets representative of the population of donors with and without T2D and to define the transcriptomic and proteomic mechanisms that underly the variation in response to each macronutrient. Our study is part of a large multi-institutional human islet deep phenotyping network that generates and links functional and multi-omic datasets for insight into normal and pathological variation in islet function. Our dataset can be accessed, browsed, and analyzed via an online platform (www.humanislets.com) that provides open access to islet donor/isolation data as well as additional phenotyping and omics datasets. Despite the high quality of our research islets, all isolated from the same center,[2] isolation parameters can have unavoidable impacts, and we found that mRNA was more sensitive than protein to technical isolation and culture parameters. Along with weak mRNA correlation to protein in our study and others',[25,26] this should give pause when inferring protein abundance (or function) from mRNA expression. Nevertheless, our dataset is the first to link deep human islet phenotyping with both transcriptomic and proteomics data, providing unprecedented and unbiased mechanistic insight into the cellular processes associated with insulin secretion in response to each nutrient class.

Our findings have relevance for personalized therapeutic nutrition and the clinical management of diabetes. For example, while both first and second phase insulin secretion in response to high glucose were blunted in islets from donors with T2D, we did not detect significantly impaired amino acid-stimulated insulin secretion, which bolsters the case that protein-rich diets could have therapeutic benefits in patients with T2D [57] and highlights the need for additional research into amino-acid stimulated insulin secretion.

A recent study sought to identify differences between islets from 17 ND donors and 12 donors with T2D but was only able to quantify 3,036 proteins, none of which were reported statistically significant after correcting for multiple comparisons.[58] Here, with more donors and more than twice the proteomic coverage, we report 555 proteins that are differentially abundant in islets from donors with T2D. This allowed us to conduct protein-centric network analysis that was not previously possible. Proteins critical to glucose and mitochondrial metabolism coupling are less abundant in islets from donors with T2D, explaining the blunted glucose-stimulated insulin secretory response. Conversely, proteins involved in cytoskeletal actin remodeling are on average more abundant in islets from donors with T2D, consistent with the role of filamentous actin limiting glucose-stimulated insulin secretion in pancreatic beta cells.[59] Identifying the pathways that are perturbed in islets from donors with T2D is crucial for better understanding the disease pathology and discovering of novel therapeutic targets.

Human islets show heterogeneous insulin secretory responses to glucose, but previous studies did not identify the source of this variation or examine other nutrients.[1–3] Here, we leveraged the heterogeneity in nutrient-stimulated insulin secretion to identify networks of proteins that drive nutrient-specific responses. Interestingly, we identified more significant correlations between protein and insulin secretion in response to physiologically relevant 6 mM glucose when compared with 15 mM, calling into question the routine use of such supraphysiological glucose concentrations *in vitro*. Heterogeneity in insulin secretion in response to the three main classes of macronutrients is reminiscent of the interindividual variability of postprandial glucose responses to carbohydrates.[9,60,61] It has been suggested that this interindividual variability in glycemic response is in part responsible for the mixed weight loss outcomes following specific diet interventions. A recent randomized clinical trial did not support this idea,[62] but post-prandial glucose and insulin responses were not measured to confirm the effectiveness of the reported personal diets. On the other hand, a diet specifically targeting hyperinsulinemia was shown to be highly effective in the prevention of multiple diseases.[7] Multi-omic profiling of blood plasma recently revealed heterogeneity in individual responses to a "healthy lifestyle intervention"[63]. Combined with our results, these studies provide the rationale for a clinical trial to test the insulin response to standardized macronutrients challenges in the general population of normoglycemic individuals and those living with T2D. One of our most surprising findings was that ~8% of donor islets showed relative hyperresponsiveness to a fat stimulus at basal glucose. Strikingly, the greatest number of correlations to protein abundance were in response to the fatty acid stimulus, meaning that these islets are quite different from those exhibiting the typical response ratio. We speculate that this lipid responsiveness may be related to beta cell immaturity because it is also observed in stem cell-derived

(F) (Left) Compares average dynamic insulin secretion stimulated by glucose (15 or 6 mM) or KCl (30 mM) between younger-immature (dashed brown lines, *n* = 8) and older-maturing (solid brown lines, *n* = 8) clusters. Basal glucose was 3 mM. Floating dot plot inserts illustrate the AUC responses for the corresponding section of the perfusion curve between younger-immature clusters (black circles) and older-maturing clusters (open circles). Error bars (SEM) are depicted by continous shaded regions around the corresponding dashed or solid lines. (Middle) Illustrates the same as (left) except stem cell-derived islet-like clusters were stimulated with leucine (5 mM) alone or in combination with 6 mM glucose as depicted in the figure panel. (Right) Illustrates the same as (left), except islet-like clusters were stimulated with oleate/palmitate (1.5 mM, 1:1 mixture) alone or in combination with 6 mM glucose.
(G and H) (G) PCA plot and (H) volcano plot of protein abundances comparing the younger-immature clusters (light green, *n* = 14) and older-maturing clusters (dark green, *n* = 10).

immature cells. In summary, we present evidence that nutrient-specific insulin secretion is heterogeneous in the general population. In the face of hyperinsulinemia being causal to numerous health conditions, our benchmark study lays the groundwork for future clinical studies aiming to advance the area of personalized therapeutic nutrition.

Limitations of the study

Despite the insights our study provides into population-level variation behind insulin secretion in response to carbohydrate, protein, and fat, we acknowledge that there are limitations to our data. For example, we recognize that family-declared T2D diagnosis at the time of organ donation is not definitive; however, it is highly unlikely that we have insulin-dependent T1D donors in our dataset as they would be outliers in our insulin abundance measurements. Another potential limitation is that our study does not have an *in vivo* human clinical trial. The magnitude of fatty acid-stimulated insulin secretion is the subject of debate,[4,14,64] and the clinical literature has not reported on individuals with higher lipid-stimulated insulin secretion compared with glucose-stimulated insulin secretion *in vivo*. However, *in vivo* studies with sufficient power and participant diversity to test this hypothesis have not been conducted and reported yet, to the best of our knowledge. A literature search of clinical studies examining the effects of glucose and oral or intravenous (i.v.) lipid infusions in human participants was only able to find studies with low n numbers that did not provide detailed information on the response distribution (Table S11),[65–70] so direct comparisons with our dataset are not possible at this time. We hope that our results will encourage clinical researchers to look for response heterogeneity for each macronutrient in heterogeneous cohorts >100. Indeed, such a trial will be essential for translating our findings into clinical practice.

STAR★METHODS

Detailed methods are provided in the online version of this paper and include the following:

- KEY RESOURCES TABLE
- RESOURCE AVAILABILITY
  - Lead contact
  - Materials availability
  - Data and code availability
- EXPERIMENTAL MODELS
  - Human islet culture
  - Mouse islet isolation and culture
  - Human embryonic stem cell-derived islet-like cluster culture and differentiation
- METHOD DETAILS
  - Deep dynamic phenotyping of human and mouse islets, and stem cell-derived islet-like clusters
  - Proteomics
  - RNAseq and Nanostring
- QUANTIFICATION AND STATISTICAL ANALYSIS
  - Co-expression analysis
  - Multi-omics data analyses
  - General statistical analyses and data visualization

SUPPLEMENTAL INFORMATION

Supplemental information can be found online at https://doi.org/10.1016/j.cmet.2024.06.001.

ACKNOWLEDGMENTS

We thank the Human Organ Procurement and Exchange (HOPE) program and Trillium Gift of Life Network (TGLN) for their work procuring human donor pancreas for research. We especially thank the organ donors and their families for their gift in support of diabetes research. The authors acknowledge that work done at UBC and BC Children's Hospital occurs on the traditional, ancestral, and unceded territories of the Coast Salish peoples, the Sḵwxwú7mesh (Squamish), səlilwətaʔɬ (Tsleil-Waututh), and xʷməθkʷəy̓əm (Musqueam) Nations. This work was supported by a Canadian Institutes for Health Research (CIHR) operating grant (168857) to J.D.J.; a CIHR Team grant (ASD-179092/5-SRA-2021-1149-S-B) to P.E.M., F.C.L., J.X., L.J.F., and J.D.J.; a CIHR-JDRF Team (ASD-173663/5-SRA-2020-1059-S-B) to F.C.L., P.E.M., and J.D.J.; and the JDRF Centre of Excellence at UBC (3-COE-2022-1103-M-B). This work was funded by Wellcome Trust grants to A.L.G. at Oxford (095101, 200837, 106130, and 203141), NIH (U01-DK105535; U01-DK085545), and by the Wellcome Trust (200837) and NIH (UM-1DK126185) at Stanford. Proteomics infrastructure was supported by the UBC Life Sciences Institute, the Canada Foundation for Innovation, the BC Knowledge Development Fund, and Genome Canada/BC (PRO264). J.K. was individually supported by a Banting Fellowship from CIHR. P.E.M. holds a Canada Research Chair in Islet Biology.

AUTHOR CONTRIBUTIONS

Conceptualization, J.K., J.X., F.C.L., A.L.G., L.J.F., P.E.M., and J.D.J.; methodology, J.K., H.H.C., J.D.E., H.S., V.R., S.S., A.F.G., J.E.M.F., J.L., L.B., J.X., F.C.L., A.L.G., L.J.F., P.E.M., and J.D.J.; investigation, J.K., R.M., J.C.R., H.S., S.S., F.C.L., and A.L.G.; visualization, J.K., W.G.S., H.H.C., J.D.E., Y.H.X., and J.D.J.; funding acquisition, J.X., F.C.L., A.L.G., L.J.F., P.E.M., and J.D.J.; supervision, J.X., F.C.L., A.L.G., L.J.F., P.E.M., and J.D.J.; writing—original draft, J.K. and J.D.J.

DECLARATION OF INTERESTS

A.L.G. declares that her spouse holds stock options in Roche and is an employee of Genentech.

Received: September 13, 2023
Revised: February 26, 2024
Accepted: June 3, 2024
Published: July 2, 2024

REFERENCES

1. Kayton, N.S., Poffenberger, G., Henske, J., Dai, C., Thompson, C., Aramandla, R., Shostak, A., Nicholson, W., Brissova, M., Bush, W.S., and Powers, A.C. (2015). Human islet preparations distributed for research exhibit a variety of insulin-secretory profiles. Am. J. Physiol. Endocrinol. Metab. *308*, E592–E602. https://doi.org/10.1152/ajpendo.00437.2014.
2. Lyon, J., Manning Fox, J.E., Spigelman, A.F., Kim, R., Smith, N., O'Gorman, D., Kin, T., Shapiro, A.M.J., Rajotte, R.V., and MacDonald, P.E. (2016). Research-Focused Isolation of Human Islets From Donors With and Without Diabetes at the Alberta Diabetes Institute IsletCore. Endocrinology *157*, 560–569. https://doi.org/10.1210/en.2015-1562.
3. Alcazar, O., and Buchwald, P. (2019). Concentration-Dependency and Time Profile of Insulin Secretion: Dynamic Perifusion Studies With Human and Murine Islets. Front. Endocrinol. (Lausanne) *10*, 680. https://doi.org/10.3389/fendo.2019.00680.
4. Cen, J., Sargsyan, E., and Bergsten, P. (2016). Fatty acids stimulate insulin secretion from human pancreatic islets at fasting glucose concentrations via mitochondria-dependent and -independent mechanisms. Nutr. Metab. (Lond.) *13*, 59. https://doi.org/10.1186/s12986-016-0119-5.
5. Henquin, J.C., Dufrane, D., and Nenquin, M. (2006). Nutrient control of insulin secretion in isolated normal human islets. Diabetes *55*, 3470–3477. https://doi.org/10.2337/db06-0868.

6. Zhang, A.M.Y., Wellberg, E.A., Kopp, J.L., and Johnson, J.D. (2021). Hyperinsulinemia in Obesity, Inflammation, and Cancer. Diabetes Metab. J. *45*, 285–311. https://doi.org/10.4093/dmj.2020.0250.

7. Wang, P., Song, M., Eliassen, A.H., Wang, M., Fung, T.T., Clinton, S.K., Rimm, E.B., Hu, F.B., Willett, W.C., Tabung, F.K., and Giovannucci, E.L. (2023). Optimal dietary patterns for prevention of chronic disease. Nat. Med. *29*, 719–728. https://doi.org/10.1038/s41591-023-02235-5.

8. Berry, S.E., Valdes, A.M., Drew, D.A., Asnicar, F., Mazidi, M., Wolf, J., Capdevila, J., Hadjigeorgiou, G., Davies, R., Al Khatib, H., et al. (2020). Human postprandial responses to food and potential for precision nutrition. Nat. Med. *26*, 964–973. https://doi.org/10.1038/s41591-020-0934-0.

9. Zeevi, D., Korem, T., Zmora, N., Israeli, D., Rothschild, D., Weinberger, A., Ben-Yacov, O., Lador, D., Avnit-Sagi, T., Lotan-Pompan, M., et al. (2015). Personalized Nutrition by Prediction of Glycemic Responses. Cell *163*, 1079–1094. https://doi.org/10.1016/j.cell.2015.11.001.

10. Canada, S. (2022). Population estimates on July 1st, by age and sex. https://www150.statcan.gc.ca/t1/tbl1/en/tv.action?pid=1710000501.

11. Henquin, J.C., Dufrane, D., Kerr-Conte, J., and Nenquin, M. (2015). Dynamics of glucose-induced insulin secretion in normal human islets. Am. J. Physiol. Endocrinol. Metab. *309*, E640–E650. https://doi.org/10.1152/ajpendo.00251.2015.

12. Basturk, B., Koc Ozerson, Z., and Yuksel, A. (2021). Evaluation of the Effect of Macronutrients Combination on Blood Sugar Levels in Healthy Individuals. Iran. J. Public Health *50*, 280–287. https://doi.org/10.18502/ijph.v50i2.5340.

13. Shukla, A.P., Iliescu, R.G., Thomas, C.E., and Aronne, L.J. (2015). Food Order Has a Significant Impact on Postprandial Glucose and Insulin Levels. Diabetes Care *38*, e98–e99. https://doi.org/10.2337/dc15-0429.

14. Gravena, C., Mathias, P.C., and Ashcroft, S.J.H. (2002). Acute effects of fatty acids on insulin secretion from rat and human islets of Langerhans. J. Endocrinol. *173*, 73–80. https://doi.org/10.1677/joe.0.1730073.

15. Ferdaoussi, M., Bergeron, V., Zarrouki, B., Kolic, J., Cantley, J., Fielitz, J., Olson, E.N., Prentki, M., Biden, T., MacDonald, P.E., and Poitout, V. (2012). G protein-coupled receptor (GPR)40-dependent potentiation of insulin secretion in mouse islets is mediated by protein kinase D1. Diabetologia *55*, 2682–2692. https://doi.org/10.1007/s00125-012-2650-x.

16. Kreutzberger, A.J.B., Kiessling, V., Doyle, C.A., Schenk, N., Upchurch, C.M., Elmer-Dixon, M., Ward, A.E., Preobraschenski, J., Hussein, S.S., Tomaka, W., et al. (2020). Distinct insulin granule subpopulations implicated in the secretory pathology of diabetes types 1 and 2. eLife *9*, e62506. https://doi.org/10.7554/eLife.62506.

17. Manders, R.J., Koopman, R., Sluijsmans, W.E., van den Berg, R., Verbeek, K., Saris, W.H., Wagenmakers, A.J., and van Loon, L.J. (2006). Co-ingestion of a protein hydrolysate with or without additional leucine effectively reduces postprandial blood glucose excursions in Type 2 diabetic men. J. Nutr. *136*, 1294–1299. https://doi.org/10.1093/jn/136.5.1294.

18. Segerstolpe, Å., Palasantza, A., Eliasson, P., Andersson, E.M., Andréasson, A.C., Sun, X., Picelli, S., Sabirsh, A., Clausen, M., Bjursell, M.K., et al. (2016). Single-Cell Transcriptome Profiling of Human Pancreatic Islets in Health and Type 2 Diabetes. Cell Metab. *24*, 593–607. https://doi.org/10.1016/j.cmet.2016.08.020.

19. Xu, J., Han, J., Long, Y.S., Epstein, P.N., and Liu, Y.Q. (2008). The role of pyruvate carboxylase in insulin secretion and proliferation in rat pancreatic beta cells. Diabetologia *51*, 2022–2030. https://doi.org/10.1007/s00125-008-1130-9.

20. Dong, G., Adak, S., Spyropoulos, G., Zhang, Q., Feng, C., Yin, L., Speck, S.L., Shyr, Z., Morikawa, S., Kitamura, R.A., et al. (2023). Palmitoylation couples insulin hypersecretion with beta cell failure in diabetes. Cell Metab. *35*, 332–344.e7. https://doi.org/10.1016/j.cmet.2022.12.012.

21. Jaikaran, E.T., and Clark, A. (2001). Islet amyloid and type 2 diabetes: from molecular misfolding to islet pathophysiology. Biochim. Biophys. Acta *1537*, 179–203. https://doi.org/10.1016/s0925-4439(01)00078-3.

22. Simpson, F., Peden, A.A., Christopoulou, L., and Robinson, M.S. (1997). Characterization of the adaptor-related protein complex, AP-3. J. Cell Biol. *137*, 835–845. https://doi.org/10.1083/jcb.137.4.835.

23. Collins, S.C., Do, H.W., Hastoy, B., Hugill, A., Adam, J., Chibalina, M.V., Galvanovskis, J., Godazgar, M., Lee, S., Goldsworthy, M., et al. (2016). Increased Expression of the Diabetes Gene SOX4 Reduces Insulin Secretion by Impaired Fusion Pore Expansion. Diabetes *65*, 1952–1961. https://doi.org/10.2337/db15-1489.

24. Sladek, R. (2023). Predicting the response to GLP-1 receptor agonists: an unexpected role for beta-arrestin-1. Lancet Diabetes Endocrinol. *11*, 3–4. https://doi.org/10.1016/S2213-8587(22)00357-6.

25. Wegler, C., Ölander, M., Wiśniewski, J.R., Lundquist, P., Zettl, K., Åsberg, A., Hjelmesæth, J., Andersson, T.B., and Artursson, P. (2020). Global variability analysis of mRNA and protein concentrations across and within human tissues. NAR Genom. Bioinform. *2*, lqz010. https://doi.org/10.1093/nargab/lqz010.

26. Jiang, L., Wang, M., Lin, S., Jian, R., Li, X., Chan, J., Dong, G., Fang, H., Robinson, A.E.; GTEx Consortium, and Snyder, M.P. (2020). A Quantitative Proteome Map of the Human Body. Cell *183*, 269–283.e19. https://doi.org/10.1016/j.cell.2020.08.036.

27. Plaisier, S.B., Taschereau, R., Wong, J.A., and Graeber, T.G. (2010). Rank-rank hypergeometric overlap: identification of statistically significant overlap between gene-expression signatures. Nucleic Acids Res. *38*, e169. https://doi.org/10.1093/nar/gkq636.

28. Szklarczyk, D., Gable, A.L., Nastou, K.C., Lyon, D., Kirsch, R., Pyysalo, S., Doncheva, N.T., Legeay, M., Fang, T., Bork, P., et al. (2021). The STRING database in 2021: customizable protein-protein networks, and functional characterization of user-uploaded gene/measurement sets. Nucleic Acids Res. *49*, D605–D612. https://doi.org/10.1093/nar/gkaa1074.

29. Warnotte, C., Nenquin, M., and Henquin, J.C. (1999). Unbound rather than total concentration and saturation rather than unsaturation determine the potency of fatty acids on insulin secretion. Mol. Cell. Endocrinol. *153*, 147–153. https://doi.org/10.1016/s0303-7207(99)00069-6.

30. Cantley, J., Davenport, A., Vetterli, L., Nemes, N.J., Whitworth, P.T., Boslem, E., Thai, L.M., Mellett, N., Meikle, P.J., Hoehn, K.L., et al. (2019). Disruption of beta cell acetyl-CoA carboxylase-1 in mice impairs insulin secretion and beta cell mass. Diabetologia *62*, 99–111. https://doi.org/10.1007/s00125-018-4743-7.

31. Mo, H., Liu, Y., Zhang, M., Qiu, Z., Li, Y., Zhang, Z., Li, Y., and Xu, G. (2022). The Role of Pancreatic Fatty Acid Synthesis in Islet Morphology and Function after Caloric Restriction or Roux-En-Y Gastric Bypass Surgery in Mice. Genes (Basel) *14*, 5. https://doi.org/10.3390/genes14010005.

32. Roomp, K., Kristinsson, H., Schvartz, D., Ubhayasekera, K., Sargsyan, E., Manukyan, L., Chowdhury, A., Manell, H., Satagopam, V., Groebe, K., et al. (2017). Combined lipidomic and proteomic analysis of isolated human islets exposed to palmitate reveals time-dependent changes in insulin secretion and lipid metabolism. PLoS One *12*, e0176391. https://doi.org/10.1371/journal.pone.0176391.

33. Mondal, A., Appu, A.P., Sadhukhan, T., Bagh, M.B., Previde, R.M., Sadhukhan, S., Stojilkovic, S., Liu, A., and Mukherjee, A.B. (2022). Ppt1-deficiency dysregulates lysosomal Ca(++) homeostasis contributing to pathogenesis in a mouse model of CLN1 disease. J. Inherit. Metab. Dis. *45*, 635–656. https://doi.org/10.1002/jimd.12485.

34. Zou, Y., Pei, J., Wang, Y., Chen, Q., Sun, M., Kang, L., Zhang, X., Zhang, L., Gao, X., and Lin, Z. (2022). The Deficiency of SCARB2/LIMP-2 Impairs Metabolism via Disrupted mTORC1-Dependent Mitochondrial OXPHOS. Int. J. Mol. Sci. *23*, 8634. https://doi.org/10.3390/ijms23158634.

35. Munk, R., Anerillas, C., Rossi, M., Tsitsipatis, D., Martindale, J.L., Herman, A.B., Yang, J.H., Roberts, J.A., Varma, V.R., Pandey, P.R., et al. (2021). Acid ceramidase promotes senescent cell survival. Aging (Albany, NY) *13*, 15750–15769. https://doi.org/10.18632/aging.203170.

36. Suurväli, J., Boudinot, P., Kanellopoulos, J., and Rüütel Boudinot, S. (2017). P2X4: A fast and sensitive purinergic receptor. Biomed J. *40*, 245–256. https://doi.org/10.1016/j.bj.2017.06.010.

37. Zhang, J., Kong, X., Zhang, Y., Sun, W., Wang, J., Chen, M., and Chen, X. (2020). FDXR regulates TP73 tumor suppressor via IRP2 to modulate aging and tumor suppression. J. Pathol. *251*, 284–296. https://doi.org/10.1002/path.5451.

38. Wang, Y., Zhu, H., and Wang, X. (2022). Prognosis and immune infiltration analysis of endoplasmic reticulum stress-related genes in bladder urothelial carcinoma. Front. Genet. *13*, 965100. https://doi.org/10.3389/fgene.2022.965100.

39. Wu, P., Mo, Y., Peng, M., Tang, T., Zhong, Y., Deng, X., Xiong, F., Guo, C., Wu, X., Li, Y., et al. (2020). Emerging role of tumor-related functional peptides encoded by lncRNA and circRNA. Mol. Cancer *19*, 22. https://doi.org/10.1186/s12943-020-1147-3.

40. Wang, X., Zhou, T., Yang, X., Cao, X., Jin, G., Zhang, P., Guo, J., Rong, K., Li, B., Hu, Y., et al. (2023). DDRGK1 Enhances Osteosarcoma Chemoresistance via Inhibiting KEAP1-Mediated NRF2 Ubiquitination. Adv Sci (Weinh). Adv. Sci. (Weinh) *10*, e2204438. https://doi.org/10.1002/advs.202204438.

41. Zhang, B., Zhao, Z., Wang, Y., Guo, T., He, M., Guan, G., Peng, P., Cai, J., Zhang, B., Liu, X., and Song, Q. (2023). STX5 Inhibits Hepatocellular Carcinoma Adhesion and Promotes Metastasis by Regulating the PI3K/mTOR Pathway. J. Clin. Transl. Hepatol. *11*, 572–583. https://doi.org/10.14218/JCTH.2022.00276.

42. Hope, C., Robertshaw, A., Cheung, K.L., Idris, I., and English, E. (2016). Relationship between HbA1c and cancer in people with or without diabetes: a systematic review. Diabet. Med. *33*, 1013–1025. https://doi.org/10.1111/dme.13031.

43. Ježek, P., Holendová, B., Jabůrek, M., Dlasková, A., and Plecitá-Hlavatá, L. (2022). Contribution of Mitochondria to Insulin Secretion by Various Secretagogues. Antioxid. Redox Signal. *36*, 920–952. https://doi.org/10.1089/ars.2021.0113.

44. Courtney, M., Gjernes, E., Druelle, N., Ravaud, C., Vieira, A., Ben-Othman, N., Pfeifer, A., Avolio, F., Leuckx, G., Lacas-Gervais, S., et al. (2013). The inactivation of Arx in pancreatic alpha-cells triggers their neogenesis and conversion into functional beta-like cells. PLoS Genet. *9*, e1003934. https://doi.org/10.1371/journal.pgen.1003934.

45. Katsumoto, K., Yennek, S., Chen, C., Silva, L.F.D., Traikov, S., Sever, D., Azad, A., Shan, J., Vainio, S., Ninov, N., et al. (2022). Wnt4 is heterogeneously activated in maturing beta-cells to control calcium signaling, metabolism and function. Nat. Commun. *13*, 6255. https://doi.org/10.1038/s41467-022-33841-5.

46. Kolberg, L., Raudvere, U., Kuzmin, I., Vilo, J., and Peterson, H. (2020). gprofiler2 – an R package for gene list functional enrichment analysis and namespace conversion toolset g:Profiler. F1000Res *9*, 9.

47. Lai, E., Bikopoulos, G., Wheeler, M.B., Rozakis-Adcock, M., and Volchuk, A. (2008). Differential activation of ER stress and apoptosis in response to chronically elevated free fatty acids in pancreatic beta-cells. Am. J. Physiol. Endocrinol. Metab. *294*, E540–E550. https://doi.org/10.1152/ajpendo.00478.2007.

48. Leung, P.S. (2003). Pancreatic renin-angiotensin system: a novel target for the potential treatment of pancreatic diseases? JOP *4*, 89–91.

49. Kolic, J., and Johnson, J.D. (2021). Promises and pitfalls of beta cell-replacement therapies. Nat. Metab. *3*, 1036–1037. https://doi.org/10.1038/s42255-021-00433-4.

50. Barsby, T., Vähäkangas, E., Ustinov, J., Montaser, H., Ibrahim, H., Lithovius, V., Kuuluvainen, E., Chandra, V., Saarimäki-Vire, J., Katajisto, P., et al. (2023). Aberrant metabolite trafficking and fuel sensitivity in human pluripotent stem cell-derived islets. Cell Rep. *42*, 112970. https://doi.org/10.1016/j.celrep.2023.112970.

51. Novakovsky, G., Sasaki, S., Fornes, O., Omur, M.E., Huang, H., Bayly, C.L., Zhang, D., Lim, N., Cherkasov, A., Pavlidis, P., et al. (2023). In silico discovery of small molecules for efficient stem cell differentiation into definitive endoderm. Stem Cell Rep. *18*, 765–781. https://doi.org/10.1016/j.stemcr.2023.01.008.

52. Jacobson, D.A., and Shyng, S.L. (2020). Ion Channels of the Islets in Type 2 Diabetes. J. Mol. Biol. *432*, 1326–1346. https://doi.org/10.1016/j.jmb.2019.08.014.

53. Puri, S., Roy, N., Russ, H.A., Leonhardt, L., French, E.K., Roy, R., Bengtsson, H., Scott, D.K., Stewart, A.F., and Hebrok, M. (2018). Replication confers beta cell immaturity. Nat. Commun. *9*, 485. https://doi.org/10.1038/s41467-018-02939-0.

54. Barsby, T., and Otonkoski, T. (2022). Maturation of beta cells: lessons from in vivo and in vitro models. Diabetologia *65*, 917–930. https://doi.org/10.1007/s00125-022-05672-y.

55. Balboa, D., Barsby, T., Lithovius, V., Saarimäki-Vire, J., Omar-Hmeadi, M., Dyachok, O., Montaser, H., Lund, P.E., Yang, M., Ibrahim, H., et al. (2022). Functional, metabolic and transcriptional maturation of human pancreatic islets derived from stem cells. Nat. Biotechnol. *40*, 1042–1055. https://doi.org/10.1038/s41587-022-01219-z.

56. Maxwell, K.G., Kim, M.H., Gale, S.E., and Millman, J.R. (2022). Differential Function and Maturation of Human Stem Cell-Derived Islets After Transplantation. Stem Cells Transl. Med. *11*, 322–331. https://doi.org/10.1093/stcltm/szab013.

57. Malaeb, S., Bakker, C., Chow, L.S., and Bantle, A.E. (2019). High-Protein Diets for Treatment of Type 2 Diabetes Mellitus: A Systematic Review. Adv. Nutr. *10*, 621–633. https://doi.org/10.1093/advances/nmz002.

58. Diamanti, K., Cavalli, M., Pereira, M.J., Pan, G., Castillejo-López, C., Kumar, C., Mundt, F., Komorowski, J., Deshmukh, A.S., Mann, M., et al. (2022). Organ-specific metabolic pathways distinguish prediabetes, type 2 diabetes, and normal tissues. Cell Rep. Med. *3*, 100763. https://doi.org/10.1016/j.xcrm.2022.100763.

59. Pigeau, G.M., Kolic, J., Ball, B.J., Hoppa, M.B., Wang, Y.W., Rückle, T., Woo, M., Manning Fox, J.E., and MacDonald, P.E. (2009). Insulin granule recruitment and exocytosis is dependent on p110gamma in insulinoma and human beta-cells. Diabetes *58*, 2084–2092. https://doi.org/10.2337/db08-1371.

60. Vega-López, S., Ausman, L.M., Griffith, J.L., and Lichtenstein, A.H. (2007). Interindividual variability and intra-individual reproducibility of glycemic index values for commercial white bread. Diabetes Care *30*, 1412–1417. https://doi.org/10.2337/dc06-1598.

61. Rein, M., Ben-Yacov, O., Godneva, A., Shilo, S., Zmora, N., Kolobkov, D., Cohen-Dolev, N., Wolf, B.C., Kosower, N., Lotan-Pompan, M., et al. (2022). Effects of personalized diets by prediction of glycemic responses on glycemic control and metabolic health in newly diagnosed T2DM: a randomized dietary intervention pilot trial. BMC Med. *20*, 56. https://doi.org/10.1186/s12916-022-02254-y.

62. Popp, C.J., Hu, L., Kharmats, A.Y., Curran, M., Berube, L., Wang, C., Pompeii, M.L., Illiano, P., St-Jules, D.E., Mottern, M., et al. (2022). Effect of a Personalized Diet to Reduce Postprandial Glycemic Response vs a Low-fat Diet on Weight Loss in Adults With Abnormal Glucose Metabolism and Obesity: A Randomized Clinical Trial. JAMA Netw Open *5*, e2233760. https://doi.org/10.1001/jamanetworkopen.2022.33760.

63. Watanabe, K., Wilmanski, T., Diener, C., Earls, J.C., Zimmer, A., Lincoln, B., Hadlock, J.J., Lovejoy, J.C., Gibbons, S.M., Magis, A.T., et al. (2023). Multiomic signatures of body mass index identify heterogeneous health phenotypes and responses to a lifestyle intervention. Nat. Med. *29*, 996–1008. https://doi.org/10.1038/s41591-023-02248-0.

64. Dos Santos, K.C., Olofsson, C., Cunha, J.P.M.C.M., Roberts, F., Catrina, S.B., Fex, M., Ekberg, N.R., and Spégel, P. (2022). The impact of macronutrient composition on metabolic regulation: an Islet-Centric view. Acta Physiol. (Oxf) *236*, e13884. https://doi.org/10.1111/apha.13884.

65. Thiébaud, D., DeFronzo, R.A., Jacot, E., Golay, A., Acheson, K., Maeder, E., Jéquier, E., and Felber, J.P. (1982). Effect of long chain triglyceride infusion on glucose metabolism in man. Metabolism *31*, 1128–1136. https://doi.org/10.1016/0026-0495(82)90163-9.

66. Jensen, C.B., Storgaard, H., Holst, J.J., Dela, F., Madsbad, S., and Vaag, A.A. (2003). Insulin secretion and cellular glucose metabolism after prolonged low-grade intralipid infusion in young men. J. Clin. Endocrinol. Metab. *88*, 2775–2783. https://doi.org/10.1210/jc.2002-021430.

67. Tricò, D., Mengozzi, A., Baldi, S., Bizzotto, R., Olaniru, O., Toczyska, K., Huang, G.C., Seghieri, M., Frascerra, S., Amiel, S.A., et al. (2022). Lipid-induced glucose intolerance is driven by impaired glucose kinetics and insulin metabolism in healthy individuals. Metabolism *134*, 155247. https://doi.org/10.1016/j.metabol.2022.155247.

68. Storgaard, H., Jensen, C.B., Vaag, A.A., Vølund, A., and Madsbad, S. (2003). Insulin secretion after short- and long-term low-grade free fatty acid infusion in men with increased risk of developing type 2 diabetes. Metabolism *52*, 885–894. https://doi.org/10.1016/s0026-0495(03)00102-1.

69. Kashyap, S., Belfort, R., Gastaldelli, A., Pratipanawatr, T., Berria, R., Pratipanawatr, W., Bajaj, M., Mandarino, L., DeFronzo, R., and Cusi, K. (2003). A sustained increase in plasma free fatty acids impairs insulin secretion in nondiabetic subjects genetically predisposed to develop type 2 diabetes. Diabetes *52*, 2461–2474. https://doi.org/10.2337/diabetes.52.10.2461.

70. Michaliszyn, S.F., Bonadonna, R.C., Sjaarda, L.A., Lee, S., Farchoukh, L., and Arslanian, S.A. (2013). beta-Cell lipotoxicity in response to free fatty acid elevation in prepubertal youth: African American versus Caucasian contrast. Diabetes *62*, 2917–2922. https://doi.org/10.2337/db12-1664.

71. Mar, S., Filatov, E., Nian, C., Sasaki, S., Zhang, D., and Lynn, F.C. (2023). Tracking insulin- and glucagon-expressing bihormonal cells during differentiation using an INSULIN and GLUCAGON double reporter human embryonic stem cell line. Preprint at bioRxiv. https://doi.org/10.1101/2023.04.19.537542.

72. Shannon, P., Markiel, A., Ozier, O., Baliga, N.S., Wang, J.T., Ramage, D., Amin, N., Schwikowski, B., and Ideker, T. (2003). Cytoscape: a software environment for integrated models of biomolecular interaction networks. Genome Res. *13*, 2498–2504. https://doi.org/10.1101/gr.1239303.

73. Kong, A.T., Leprevost, F.V., Avtonomov, D.M., Mellacheruvu, D., and Nesvizhskii, A.I. (2017). MSFragger: ultrafast and comprehensive peptide identification in mass spectrometry-based proteomics. Nat. Methods *14*, 513–520. https://doi.org/10.1038/nmeth.4256.

74. Yu, F., Haynes, S.E., Teo, G.C., Avtonomov, D.M., Polasky, D.A., and Nesvizhskii, A.I. (2020). Fast Quantitative Analysis of timsTOF PASEF Data with MSFragger and IonQuant. Mol. Cell. Proteomics *19*, 1575–1585. https://doi.org/10.1074/mcp.TIR120.002048.

75. da Veiga Leprevost, F., Haynes, S.E., Avtonomov, D.M., Chang, H.Y., Shanmugam, A.K., Mellacheruvu, D., Kong, A.T., and Nesvizhskii, A.I. (2020). Philosopher: a versatile toolkit for shotgun proteomics data analysis. Nat. Methods *17*, 869–870. https://doi.org/10.1038/s41592-020-0912-y.

76. Lyon, J., Spigelman, A.F., Manning Fox, J.E., and MacDonald, P.E. (2021). ADI Isletcore protocols for the isolation, assessment and cryopreservation of human pancreatic islets of langerhans for research purposes. Protocols.io. https://doi.org/10.17504/protocols.io.bupanvie.

77. Castro-Gutierrez, R., Alkanani, A., Mathews, C.E., Michels, A., and Russ, H.A. (2021). Protecting Stem Cell Derived Pancreatic Beta-Like Cells From Diabetogenic T Cell Recognition. Front. Endocrinol. (Lausanne) *12*, 707881. https://doi.org/10.3389/fendo.2021.707881.

78. Kolic, J., Beet, L., Overby, P., Cen, H.H., Panzhinskiy, E., Ure, D.R., Cross, J.L., Huizinga, R.B., and Johnson, J.D. (2020). Differential Effects of Voclosporin and Tacrolimus on Insulin Secretion From Human Islets. Endocrinology *161*, bqaa162. https://doi.org/10.1210/endocr/bqaa162.

79. Demichev, V., Messner, C.B., Vernardis, S.I., Lilley, K.S., and Ralser, M. (2020). DIA-NN: neural networks and interference correction enable deep proteome coverage in high throughput. Nat. Methods *17*, 41–44. https://doi.org/10.1038/s41592-019-0638-x.

80. Bern, M.W., and Kil, Y.J. (2011). Two-dimensional target decoy strategy for shotgun proteomics. J. Proteome Res. *10*, 5296–5301. https://doi.org/10.1021/pr200780j.

81. Demichev, V., Szyrwiel, L., Yu, F., Teo, G.C., Rosenberger, G., Niewienda, A., Ludwig, D., Decker, J., Kaspar-Schoenefeld, S., Lilley, K.S., et al. (2022). dia-PASEF data analysis using FragPipe and DIA-NN for deep proteomics of low sample amounts. Nat. Commun. *13*, 3944. https://doi.org/10.1038/s41467-022-31492-0.

82. van de Bunt, M., Manning Fox, J.E., Dai, X., Barrett, A., Grey, C., Li, L., Bennett, A.J., Johnson, P.R., Rajotte, R.V., Gaulton, K.J., et al. (2015). Transcript Expression Data from Human Islets Links Regulatory Signals from Genome-Wide Association Studies for Type 2 Diabetes and Glycemic Traits to Their Downstream Effectors. PLoS Genet. *11*, e1005694. https://doi.org/10.1371/journal.pgen.1005694.

83. Lamble, S., Batty, E., Attar, M., Buck, D., Bowden, R., Lunter, G., Crook, D., El-Fahmawi, B., and Piazza, P. (2013). Improved workflows for high throughput library preparation using the transposome-based Nextera system. BMC Biotechnol. *13*, 104. https://doi.org/10.1186/1472-6750-13-104.

84. Yoon, J.S., Sasaki, S., Velghe, J., Lee, M.Y.Y., Winata, H., Nian, C., and Lynn, F.C. (2022). Calcium-dependent transcriptional changes in human pancreatic islet cells reveal functional diversity in islet cell subtypes. Diabetologia *65*, 1519–1533. https://doi.org/10.1007/s00125-022-05718-1.

85. Stekhoven, D.J., and Bühlmann, P. (2012). MissForest–non-parametric missing value imputation for mixed-type data. Bioinformatics *28*, 112–118. https://doi.org/10.1093/bioinformatics/btr597.

86. Love, M.I., Huber, W., and Anders, S. (2014). Moderated estimation of fold change and dispersion for RNA-seq data with DESeq2. Genome Biol. *15*, 550. https://doi.org/10.1186/s13059-014-0550-8.

87. Leek, J.T., Johnson, W.E., Parker, H.S., Fertig, E.J., Jaffe, A.E., Zhang, Y., Storey, J.D., and Collado Torres, L. (2022).). sva: Surrogate Variable Analysis. R package version 3.46.0.

88. Langfelder, P., and Horvath, S. (2008). WGCNA: an R package for weighted correlation network analysis. BMC Bioinformatics *9*, 559. https://doi.org/10.1186/1471-2105-9-559.

89. Kim, S. (2015). ppcor: An R Package for a Fast Calculation to Semi-partial Correlation Coefficients. Commun. Stat. Appl. Methods *22*, 665–674. https://doi.org/10.5351/CSAM.2015.22.6.665.

90. Yu, G., Wang, L.G., Han, Y., and He, Q.Y. (2012). clusterProfiler: an R package for comparing biological themes among gene clusters. Omics *16*, 284–287. https://doi.org/10.1089/omi.2011.0118.

91. van Gurp, L., Fodoulian, L., Oropeza, D., Furuyama, K., Bru-Tari, E., Vu, A.N., Kaddis, J.S., Rodríguez, I., Thorel, F., and Herrera, P.L. (2022). Generation of human islet cell type-specific identity genesets. Nat. Commun. *13*, 2020. https://doi.org/10.1038/s41467-022-29588-8.

STAR★METHODS

KEY RESOURCES TABLE

REAGENT or RESOURCE	SOURCE	IDENTIFIER
Biological samples		
Human Islet of Langerhans	Alberta Diabetes IsletCore	Table S1
Stem Cell-Derived Islets	Mar et al.[71]	Data S1-Source Data
C57BL/6J mice	The Jackson Laboratory	RRID:IMSR_JAX:000664
Critical commercial assays		
Human Insulin-Specific RIA	Millipore Sigma	Cat. # HI-14K; RRID:AB_2801577
Rat Insulin RIA	Millipore Sigma	Cat. # RI-13K; RRID:AB_2884035
Deposited data		
RNAseq	This paper	European Genome-phenome Archive (EGA): EGAS00001007241
Proteomics	This paper	ProteomeXchange via MassIVE: PXD045422
Perifusion Functional Dataset	This paper	Data S1-Source Data
Software and algorithms		
R (version 4.1.1)	R Core Team	https://www.r-project.org
Adobe Illustrator	Adobe Systems	https://www.adobe.com/products/illustrator.html
Cytoskape	Shannon et al.[72]	https://cytoscape.org
FragPipe (version 3.4)	MSFragger[73,74]	https://fragpipe.nesvilab.org
Philosopher (version 3.8)	MSFragger[75]	https://philosopher.nesvilab.org
GraphPad Prism 9	Graphpad Software	https://www.graphpad.com
Rank Rank Hypergeometric Overlap	The Graeber Lab	https://systems.crump.ucla.edu/rankrank/

RESOURCE AVAILABILITY

Lead contact

Further information and requests for reagents should be directed and will be fulfilled by the lead contact, James D. Johnson (james.d.johnson@ubc.ca).

Materials availability

The study did not generate new unique reagents.

Data and code availability

- All data are available, both via a purpose-build web portal (www.humanislets.com) and the appropriate public databases. The raw RNA sequencing data has been deposited in the European Genome-phenome Archive (EGA) under study number EGAS00001007241. The raw proteomics data has been deposited to ProteomeXchange via MassIVE under dataset number PXD045422. Source raw data are provided with this paper (Data S1 – Source Data).
- All original code has been deposited at Github repository https://github.com/hcen/Kolic_macronutrients and is publicly available as of the date of publication.
- Any additional information required to reanalyze the data reported in this paper is available from the lead contact upon request.

EXPERIMENTAL MODELS

Human islet culture

Islets were isolated from the pancreas of cadaveric human donors at the Alberta Diabetes Institute IsletCore and used with approval of the Human Research Ethics Board at the University of Alberta (Pro00013094; Pro00001754), the University of British Columbia (UBC) Clinical Research Ethics Board (H13-01865), the University of Oxford's Oxford Tropical Research Ethics Committee

(OxTREC Ref.2–15), the Oxfordshire Regional Ethics Committee B (REC reference: 09/H0605/2), or by the Stanford Center for Biomedical Ethics (IRB Protocol: 57310). T2D diagnosis was family-declared at the time of organ donation. All families of organ donors provided written informed consent for use in research. Islet isolation was performed according to the detailed methods deposited in the protocols.io repositor.[76] Islets were shipped in CMRL media (Thermo Fisher Scientific) overnight from Edmonton to UBC. Upon arrival, islets were immediately purified by handpicking under a stereomicroscope and suspended in RPMI 1640 medium (Thermo Fisher Scientific, Cat#, 11879-020) supplemented with 5.5 mmol glucose, 10% fetal bovine serum (FBS), and 100 units/mL penicillin/streptomycin. Islets were cultured in 10 cm non-adhesive petri dishes (Thermo Fisher Scientific, Cat # FB0875713) for 24-72 hours prior to experiments to allow for recovery from shipment. Total islet culture time (as well as additional donor and isolation characteristics) are listed in Table S1.

Mouse islet isolation and culture

C57BL/6J mice were purchased from the Jackson Laboratory and housed in the UBC Modified Barrier Facility (temperature-controlled) using protocols approved by the UBC Animal Care Committee and in accordance with international guidelines. The mice were on a 12-hour light/dark cycle with chow diet (LabDiet #5053) and drinking water *ad libitum*. Mouse islets were isolated by ductal collagenase (Type XI; Sigma, Cat. # C7657) injection followed by hand-picking. To allow for recovery post digestion, islets were cultured overnight in islet culture media (RPMI media with 11.1 mM D-glucose supplemented with 10% vol/vol fetal bovine serum (Thermo Fisher Scientific, Cat. #12483020) and 1% vol/vol Penicillin-Streptomycin (Gibco, Cat #15140-148)) at 37°C with 5% CO_2.

Human embryonic stem cell-derived islet-like cluster culture and differentiation

A human embryonic stem cell line, based on the WiCell WA01 line, that contained knock-in add-on of EGFP downstream of the insulin coding sequence[51] was differentiated, as previously described, through a 6-stage protocol to day 21, media components are shown in Table S12.[51,77] After the initial 21 days of differentiation, cells were cultured in CMRL with 5.6 mM glucose (Thermo Fisher Scientific) containing 1% fatty acid-free BSA (Proliant), 1:100 Glutamax, 1:100 NEAA, 1 mM pyruvate, 10 mM HEPES, 1:100 ITS (Thermo Fisher Scientific), 10 μg/ml of heparin sulfate, 1 mM N-acetyl cysteine, 10 μM zinc sulfate, 1.75 μL 2-mercaptoethanol, 2 μM T3 and 0.155 mM ascorbic acid (Sigma Aldrich) until Day 27. Between Day 28-50, aggregates were grown in CMRL with 5.6 mM glucose containing 1% fatty acid-free BSA, 1:100 Glutamax, 1:100 NEAA, 1 mM Pyruvate, 10 mM HEPES, 1:100 ITS, 10 μg/ml heparin sulfate, 1 mM N-acetyl cysteine, 10 μM zinc sulfate, 1.75 μL 2-mercaptoethanol, 10 nM T3, 1:2000 Trace elements A (Cellgro), 1:2000 trace elements B (Cellgro), 1:2000 Lipid Concentrate (Thermo Fisher Scientific) and 0.5 μM ZM447439 (Selleckchem).[55]

Dispersion/reaggregation of the stem cell-derived aggregates was carried out on Day 25 as previously described.[71] Briefly, aggregates were dissociated with Accumax (Thermo Fisher Scientific) for 8 minutes at 37 °C with occasional shaking. Cells were washed with DPBS, centrifuged (5min/200xg) then resuspended in DPBS with 0.2% FBS and 10μM Y-27632. Cells were filtered through a strainer-capped FACS tube prior to FACS purification of the GFP+ fraction. FAC-sorted cells were collected in Stage 6.2 media containing 10μM Y-27632 and then aggregated in AggreWell 400 24-well plates (STEMCELL Technologies) using the manufacturer's protocol at 1000 cells per aggregate.

METHOD DETAILS

Deep dynamic phenotyping of human and mouse islets, and stem cell-derived islet-like clusters

Our standard approach[78] compared the response to 6 or 15 mM glucose stimulation and direct depolarization with 30 mM KCl. In parallel, we also measured insulin secretion in response to 5 mM leucine (Sigma, Cat. # L8912, dissolved in 1M HCl, then pH adjusted with 1M NaOH) – an essential branched chain amino acid previously shown to increase insulin secretion in human islets[5]; or a 1:1 mixture of 0.75 mM oleic acid (Sigma, Cat. # 364525) and 0.75 mM palmitic acid (Sigma, Cat. #P5585) at either 3 mM or 6 mM glucose (see figure legends). Fatty acids stock solutions were prepared in 50% ethanol at 65°C for 30 minutes, and then added to a solution of fatty acid free bovine serum albumin (BSA) (Sigma, Cat. # A7030) in a 6:1 molar ratio in a 37 °C water bath for 60 min.

We loaded perifusion columns with either 65 human islets, 100 (hESC)-stem cell-derived islet-like clusters, or 100 mouse islets and perifused them at 0.4 mL/min with 3 mM glucose Krebs-Ringer Modified Buffer (KRB) solution as described previously[78] for 60 minutes to equilibrate the islets to the KRB and flow rate, and then with the indicated conditions. First-phase insulin release was defined as the amount of insulin secreted during the first 15 minutes of 15 mM glucose stimulation, while the remaining 25 min of stimulation were defined as second-phase release. Peak insulin secretion was defined as single point whereby the amount of insulin released during the first 15 minutes of a solution change was the highest. Samples were stored at -20 °C and insulin secretion was quantified using human (Millipore Cat. # HI-14K) or rat (Millipore Cat. # RI-13K) insulin radioimmunoassay kits. All insulin secretion units have been converted to pmol/islet, to allow for direct comparison between species.

Proteomics

Library generation

We generated a pooled protein sample of 10 non-diabetic donors (as indicated in Table S1) at a concentration of 1.5ug/uL. Briefly, 100 μl of SDS lysis buffer (4 % SDS, 100 mM Tris, pH = 8) was added to each sample. Samples were then vortexed for 1 minute, boiled at 100°C for 10 minutes, vortexed again for 1 minute and then centrifuged at 4°C at 10,000 RPM for 10 minutes. The supernatants

were collected and tested for protein concentration by BCA protein assay (ThermoFisher Scientific, Cat. # PI-23225). Measurements were performed on a Spark plate reader (TECAN). All protein samples were stored at -80°C.

Next, 297 μg of the pooled protein sample was used for further processing. The Trapped Ion Mobility – Time of Flight Mass Spectrometer (TimsTOF Pro; Bruker Daltonics, Germany) was set to Parallel Accumulation-Serial Fragmentation (PASEF) scan mode for DDA acquisition scanning 100 – 1700 m/z with 5 PASEF ramps. The capillary voltage was set to 1800V, drying gas to 3L/min, and drying temperature to 180°C. The MS and MS/MS spectra were acquired from m/z 100 – 1700. As for TIMS setting, ion mobility range (1/k0) was set to 0.70 – 1.35 V•s/cm^2, 100ms ramp time and accumulation time (100% duty cycle), and ramp rate of 9.42Hz; this resulted in 0.64s of total cycle time.

Linear precursor repetitions were applied with a target intensity of 21,000 and 2500 intensity threshold. The active exclusion was enabled with a 0.4 min release. The collision energy was ramped linearly as a function of mobility from 27eV at 1/k0 = 0.7 V•s/cm^2 to 55eV at 1/k0 = 1.35 V•s/cm^2. Isolation widths were set at 2.07 m/z at < 400 m/z and 3.46 m/z at > 1000 m/z. Mass accuracy: error of mass measurement is typically within 3 ppm and is not allowed to exceed 7 ppm. For calibration of ion mobility dimension, the ions of Agilent ESI-Low Tuning Mix ions were selected (m/z [Th], 1/k0 [Th]: 622.0290, 0.9915; 922.0098, 1.1986; 1221.9906, 1.3934). TimsTOF Pro was run with timsControl v. 3.0.0 (Bruker). LC and MS were controlled with HyStar 6.0 (6.0.30.0, Bruker).

Acquired data were then searched using FragPipe[79] computational platform (v. 17.1) with MSFragger[73,74] (v. 3.4), Philosopher[75] (v. 3.8), and EasyPQP (v. 0.1.27) components to build a spectral library. Protein sequence database Homo sapiens from Uniprot (reviewed sequences only; downloaded on January 15, 2021) and common contaminants protein, containing in total 20428 sequences were used, where reversed protein sequences were appended to the original database as decoys. For the MSFragger analysis, both precursor and fragment mass tolerances were set to 50 ppm. Enzyme specificity was set to "trypsin", with up to 2 missed cleavages allowed.

For each analysis, the MS/MS search results were further processed using Philosopher, where final reports were generated and filtered at 1% protein FDR plus 1% PSM/ion/peptide-level FDR[80] for each corresponding PSM.tsv, ion.tsv, and peptide.tsv files. Finally.tsv files were used as input to EasyPQP for the generation of consensus spectrum libraries. The final spectral library was filtered to 1% protein and 1% peptide-level FDR.

Experimental samples

For each donor approximately 300 hand-picked islets were transferred into a 1.5 ml microcentrifuge tube. Culture media was removed, islets were washed twice in 1X PBS (Invitrogen, Cat. #10010049) and cell pellets were immediately flash frozen in liquid nitrogen and then stored at -80°C. Samples were processed and protein was extracted and measured as described above.

Next, 20 μg of each lysed sample was taken for further processing. Reduction of disulfide bonds was done by incubation with DTT (30 mM) for 35 min at 37°C, followed by alkylation by CAA (50 mM) for 20 min at 37°C. Samples were loaded onto 10% Mini-PROTEAN® TGX™ Precast Protein Gels (BioRad) and run for 30 min at 85V. Proteins were visualized by Coomassie blue (stained for 20 min). Lanes were cut out and destained in Ambic:EtOH (60:40), dehydrated and digested with Trypsin, first round 0.32ug/lane for 15 hours at 37°C, second round 0.128ug/lane for 2 hours, at 37°C (0.448ug in total/lane). Digestion was stopped with 10% FA, and samples were extracted with a series of extraction solutions, twice by 50% ACN, 50% of 0.1% TFA, and twice by 80% ACN, 20% of 0.1% TFA. Samples were then concentrated via vacuum centrifugation. Extracted peptide samples were then cleaned up via STAGE-tip purification, briefly: Resolubilized acidified sample was forced through a conditioned and equilibrated column with 8-12 mm of C18 packing, washed with 1% TFA twice, and eluted into clean plates by buffer containing 40% ACN, 0.1% TFA, then dried down.

Each sample was reconstituted in 0.5% ACN, 0.1% formic acid for LC-MS/MS analysis of 150 ng total on-column injections (with n = 3 technical replicates). The digest was separated using NanoElute UHPLC system (Bruker Daltonics) with Aurora Series Gen2 (CSI) analytical column (25 cm x 75 μm 1.6 μm FSC C18, with Gen2 nanoZero and CSI fitting; Ion Opticks, Parkville, Victoria, Australia) heated to 50°C and coupled to a Trapped Ion Mobility – Time of Flight mass spectrometer (timsTOF Pro; Bruker Daltonics, Germany) operated in Data-Independent Acquisition - Parallel Accumulation-Serial Fragmentation (DIA-PASEF) mode. A standard 60 min gradient was run from 2% B to 12% B over 30 min, then to 33% B from 30 to 60 min, then to 95% B over 0.5 min, and held at 95% B for 7.72 min. Before each run, the analytical column was conditioned with 4 column volumes of buffer A. Where buffer A consisted of 0.1% aqueous formic acid and 0.5 % acetonitrile in water, and buffer B consisted of 0.1% formic acid in 99.4 % acetonitrile. The NanoElute thermostat temperature was set at 7°C. The analysis was performed at 0.3 μL/min flow rate.

The TimsTOF Pro was set to Parallel Accumulation-Serial Fragmentation (PASEF) scan mode for DIA acquisition scanning 100 – 1700 m/z. The capillary voltage was set to 1800V, drying gas to 3L/min, and drying temperature to 180°C. The MS1 scan was followed by 17 consecutive PASEF ramps containing 22 non-overlapping 35 m/z isolation windows, covering the m/z range 319.5 – 1089.5 (more information in DIA windows). As for TIMS setting, ion mobility range (1/k0) was set to 0.70 – 1.35 V•s/cm^2, 100ms ramp time and accumulation time (100% duty cycle), and ramp rate of 9.42 Hz; this resulted in 1.91s of total cycle time. The collision energy was ramped linearly as a function of mobility from 27eV at 1/k0 = 0.7 V•s/cm^2 to 55eV at 1/k0 = 1.35 V•s/cm^2. Mass accuracy: error of mass measurement is typically within 3 ppm and is not allowed to exceed 7 ppm. For calibration of ion mobility dimension, the ions of Agilent ESI-Low Tuning Mix ions were selected (m/z [Th], 1/k0 [Th]: 622.0290, 0.9915; 922.0098, 1.1986; 1221.9906, 1.3934). TimsTOF Pro was run with timsControl v. 3.0.0 (Bruker). LC and MS were controlled with HyStar 6.0 (6.0.30.0, Bruker).

Acquired diaPASEF data were then searched using FragPipe[75] computational platform (v. 17.1) with MSFragger[80,81] (v. 3.4), Philosopher[75] (v. 3.8), EasyPQP (v. 0.1.27) and DIA-NN (v. 1.8) to obtain DIA quantification, with use of the spectral library generated from the before mentioned highly fractionated sample. Quantification mode was set to "Any LC (high precision)". All other settings were left default.

RNAseq and Nanostring

Islets for RNA-seq were processed in two centers, initially in Oxford (n=49) with later donors being processed at Stanford (n = 47). For those processed in Oxford the methods have been described.[82] Briefly, freshly isolated human islets were collected at the Alberta Diabetes Institute IsletCore (www.isletcore.ca) in Edmonton, Canada. Freshly isolated islets were processed for RNA and DNA extraction after 1-3 days in culture in CMRL media. RNA was extracted from human islets using Trizol (Ambion, UK or Sigma-Aldrich, Canada). To clean remaining media from the islets, samples were washed three times with phosphate-buffered saline (Sigma-Aldrich, UK). After the final cleaning step 1 mL Trizol was added to the cells. The cells were lysed by pipetting immediately to ensure rapid inhibition of RNase activity and incubated at room temperature for 10 min. Lysates were then transferred to clean 1.5 mL RNase-free centrifuge tubes (Applied Biosystems, UK). RNA quality (RIN score) was determined using an Agilent 2100 Bioanalyser (Agilent, UK), with a RIN score > 6 deemed acceptable for inclusion in the study. Samples were stored at −80 °C prior to sequencing. PolyA selected libraries were prepared from total RNA at the Oxford Genomics Centre using NEBNext ultra directional RNA library prep kit for Illumina with custom 8 bp indexes.[83] Libraries were multiplexed (three samples per lane), clustered using TruSeq PE Cluster Kit v3, and paired-end sequenced (75bp) using Illumina TruSeq v3 chemistry on the Illumina HiSeq2000 platform. For the samples processed at Stanford, freshly isolated human islets were collected at the Alberta Diabetes Institute IsletCore in Edmonton, Canada, the same as the Oxford samples. The islets were picked to >95% purity and washed with PBS, then stored in 1 mL of Trizol (Ambion, UK). Islets in trizol were stored at -80 C until they were processed for RNA using the phenol-chloroform extraction method. The extracted RNA was resuspended in water and then dnased to remove contaminating DNA. RNA quality (RIN score) and concentration were determined using an Agilent 2100 Bioanalyzer (Agilent, US), with a RIN score > 5 deemed acceptable for inclusion in the study. Samples were stored at −80 °C prior to sequencing. PolyA selected cDNA libraries were prepared from total RNA and then sequenced using NovaSeq 6000 by Novogene. The raw sequencing data (fastq files) were checked first in regarding of the number of reads (>= 20 million per sample and extra sequencing were performed if necessary) and then in regarding of quality control, including quality score per base and length distribution using fastqc (v0.11.9). Reads were aligned to human genome reference (GRCh38) using STAR (v2.7.9a) with ENSEMBL gene annotations (v101). Gene expression levels were counted using featureCounts (v2.0.1) on exonic reads only. Differential expression was compared using the Wald test in DESeq2 (v1.26.0). *p* values were adjusted using the Benjamini and Hochberg method.

Fifty islets from each donor batch were used to assess islet quality by profiling expression of 132 human islet genes as described.[84] Gene expression was measured using nCounter prep kits and nCounter SPRINT profiler according to manufacturer's protocol (NanoString, USA).

QUANTIFICATION AND STATISTICAL ANALYSIS

Co-expression analysis

Analysis was performed with both RNA-seq and proteomics data to assess relationships between 'modules' of omics features and donor metadata, including technical islet isolation parameters, clinical metadata, and functional outcomes. There were three main steps: data processing, network construction and functional characterization, and module-donor metadata correlation analysis.

Raw protein abundance matrices (134 donors, 8489 proteins; Uniprot IDs) were filtered to remove proteins with greater than 50% missing values, reducing the number of proteins to 7919. Next, the proteomics data were normalized by sample median, log transformed (base 10), and missing values were imputed with the *missForest* R package[85] using a random forest-based algorithm. The RNA-seq counts matrix (96 donors, 17 673 genes; Entrez IDs) was filtered based on abundance (15% with lowest mean counts removed), normalized with the 'variance stabilizing transformation' from the *DeSeq2* R package,[86] and then filtered based on variance (15% with lowest variance removed). The RNA-seq data was measured in seven batches; batch effects were adjusted for using the ComBat method in the *sva* R package.[87] One of the batches ($n = 6$ samples) had a sequencing depth of about 50% compared to the other samples, resulting in ~5000 genes with zero counts that prevented effective batch effect adjustment. These six samples were removed from the dataset. Donor metadata included clinical metadata (age, sex, BMI, diabetes diagnosis, HbA1c), technical islet isolation metadata (culture time, cold ischemia time, digestion time, purity, and insulin content), and functional outcomes (AUC values from perifusion experiments after glucose, leucine, and oleate/palmitate exposures). Continuous variables with right-skew distributions were log-transformed (base 10). A constant value was added to the perifusion outcomes that had negative values to ensure all data were > 0 before log-transformation.

Two co-expression networks were constructed using the same methods, one for each omics type, using the WGCNA R package.[88] First, the soft threshold parameter was selected by choosing the lowest value at which the R^2 value of the scale free topology model fit did not substantially increase (threshold parameter = 8 and 20 for proteomics and RNA-seq respectively; selected via visual examination of an elbow plot). Next, pairwise distances between omics features were calculated by computing signed adjacency and topological overlap matrices using the soft threshold parameter and the 'bicor' correlation function. Finally, modules of co-expressed (or co-abundant) features were defined by first hierarchically clustering features based on the distance matrix, and then detecting

modules of densely connected features using the dynamic tree cut algorithm. Modules were annotated with KEGG pathways and GO terms (biological process, cellular component, and molecular function) by performing overrepresentation analysis with the list of features in each module.

The pairwise partial correlation between module expression/abundance levels and donor metadata, accounting for the technical isolation parameters (purity, culture time, digestion time, and cold ischemia time), were calculated with the partial Pearson correlation function in the *ppcor* R package.[89] Module expression/abundance levels were summarized with *WGCNA* ‘eigengenes’, which are the first principal component scores for each module, re-scaled so that positive and negative scores can be interpreted as higher and lower expression/abundance levels respectively. Correlation p-values were adjusted using the Benjamini-Hochberg method, with the FDR set to 5%.

Multi-omics data analyses

RNAseq data was analyzed by DESeq2 R package (version 1.36.0).[86] Six samples exhibiting large batch difference were excluded, leaving a total of 90 individuals (82 ND and 8 T2D) included in the analyses. Genes with low counts (<5 raw counts in more than 50% samples) were removed. Raw counts of the genes were normalized by variance stabilizing transformation (VST). T2D group was contrasted to ND group, including batch as a covariate in the default Wald test. Genes with Benjamini-Hochberg adjusted p-values < 0.05 were considered as differentially expressed.

For proteomics data analyses, proteins that were undetected in more than 50% samples were removed. Benjamini-Hochberg adjusted p-values < 0.05 of student t-test were used to identify differentially expressed proteins between the various identified groups.

To identify proteins associated with islet parameters, linear regression was conducted using $\log_2$ protein abundances of each protein as a dependent variable, each islet parameter as an independent variable, and diabetes status as a covariate to adjust for the effect of T2D. The coefficients of islet parameters and their FDR-adjusted p-values were reported. Similarly, to identify RNAs associated with islet parameters, the VST normalized gene counts were used as dependent variables instead. The batches of sequencing were included as additional covariates. Pearson correlations were also conducted instead of linear regression.

To assess the how well RNA expression reflects the abundance of the corresponding protein, we conducted Pearson correlation between proteins and RNAs. For across-gene correlation, mean protein abundances and mean RNA TPMs (transcript per million) were correlated. We used protein signal/protein length as a raw estimate of relative abundances across different proteins and TPM as an estimate of relative RNA levels across different genes. For within-gene across sample correlations, we correlated each gene’s VST normalized RNA counts with $\log_2$ protein abundances, and the p values were adjusted by Benjamini-Hochberg. Instead of Pearson correlation, we also checked within-gene association using linear regression adjusting for batches of sequencing, and the results were mostly the same (not shown).

Gene set enrichment analysis (GSEA) was performed on the transcriptomic or proteomics changes (T2D/ND) using KEGG or Hallmark gene set libraries. The direction-signed $-\log_{10}$(p values) were used as rank scores. Over representation analysis (ORA) was performed on genes with positive, negative or non-significant RNA-protein correlations using Gene Ontology – Cellular Component (GO-CC) gene set library. Genes that are commonly detected in RNAseq and proteomics were used as background gene list. Both GSEA and ORA were conducted in clusterProfiler R package.[90]

To evaluate the concordant changes of RNAs and proteins in T2D, in addition to comparing differentially expressed RNAs and proteins, we applied the threshold-free method called rank-rank hypergeometric overlap test.[27] RNAs and proteins were ranked by direction-signed $-\log_{10}$(p value) or $\log_2$ fold change (T2D/ND). The ranked lists were processed in the online tool (https://systems.crump.ucla.edu/rankrank/) and RRHO R package using a step size of 100.

Cell type deconvolution was conducted using the BisqueRNA R package using a marker-based decomposition approach. RNAseq DESeq2 normalized counts (without VST) or protein abundances (without $\log_2$ transformation) were used as the input. Islet cell type markers (alpha, beta, gamma, delta) were from van Gurp et al.,[91] and acinar cell markers were from Segerstolpe.[18]

General statistical analyses and data visualization

Statistical analyses and data presentation for perifusion studies of dynamic insulin secretion were carried out using GraphPad Prism 9 (Graphpad Software, San Diego, CA, USA) or R (v 4.1.1). using Student’s *t*-test for parametric data and Mann-Whitney test for non-parametric data. For all statistical analyses, differences were considered significant if the p-value was less than 0.05: * p< 0.05; ** p< 0.01; *** p< 0.001; **** p<0.0001. Data were presented as means ± SEM with individual data points from biological replicates (average of two technical replicates), unless otherwise indicated in the figure legends.

Cell Metabolism, Volume *36*

Supplemental information

Proteomic predictors of individualized nutrient-specific insulin secretion in health and disease

Jelena Kolic, WenQing Grace Sun, Haoning Howard Cen, Jessica D. Ewald, Jason C. Rogalski, Shugo Sasaki, Han Sun, Varsha Rajesh, Yi Han Xia, Renata Moravcova, Søs Skovsø, Aliya F. Spigelman, Jocelyn E. Manning Fox, James Lyon, Leanne Beet, Jianguo Xia, Francis C. Lynn, Anna L. Gloyn, Leonard J. Foster, Patrick E. MacDonald, and James D. Johnson

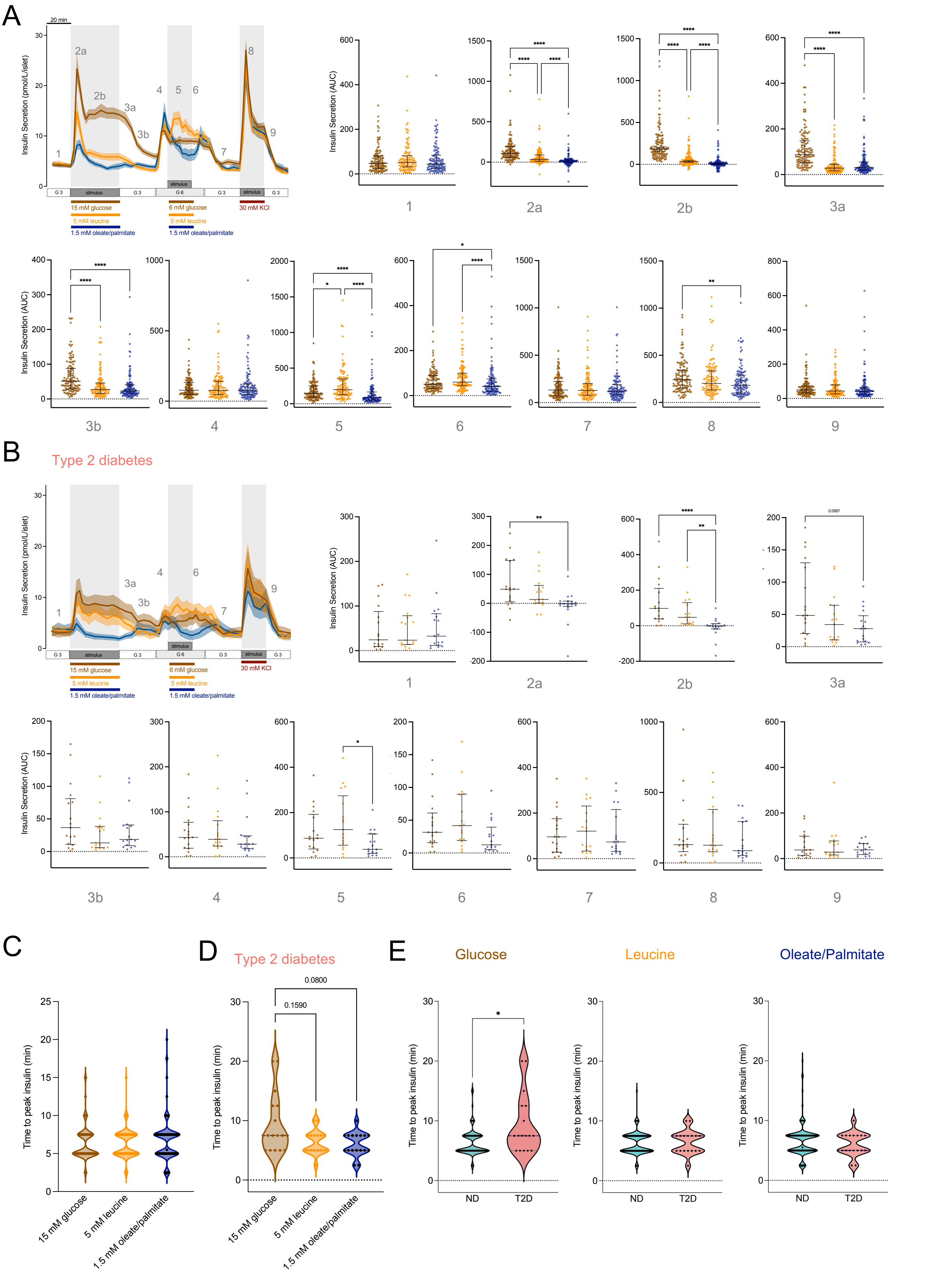

A
B
Type 2 diabetes
C
D
Type 2 diabetes
E
Glucose
Leucine
Oleate/Palmitate
Insulin Secretion (pmol/L/islet)
Insulin Secretion (AUC)
Time to peak insulin (min)
15 mM glucose
5 mM leucine
1.5 mM oleate/palmitate
6 mM glucose
30 mM KCl
20 min
stimulus
G 3
G 6
ND
T2D
0.1590
0.0800
0.0957

Figure S1

Fig. S1. Related to Figure 1: Average dynamic insulin secretion perifusion AUC calculations in non-diabetic donors and donors with type-2 diabetes. (A) Averaged traces of dynamic insulin secretion measurements in response to glucose (15 or 6 mM), leucine (5 mM), oleate and palmitate (1.5 mM. 1:1 mixture) or KCl (30 mM) in islets isolated from ND donors (n=123). Numbers in grey represent the following time intervals: 1. baseline (0-20 min) 2a. 1st phase (20-35 min) 2b. second phase (35-60 min) 3a. 3mM glucose (60-75 min) 3b. 3mM glucose (75-90 min) 4. 6 mM glucose (90-100 min) 5. 6 mM glucose (100-120 min) 6. 6 mM glucose (120-130 min) 7. 3mM glucose (130-160 min) 8. 30 mM KCl (160-180 min) and 9. 3 mM glucose (190-200 min). Corresponding areas under the curve (AUC) for each indicated section, with median and interquartile range are shown. **(B)** Shows the same as (A) except in islets from donors previously diagnosed with T2D (n=17). **(C)** Time-to-reach peak insulin secretion following a 15 mM glucose stimulus (brown), 5 mM leucine stimulus (orange) or 1.5 mM oleate/palmitate stimulus (blue) in non-diabetic donors (n=123). **(D)** Shows the same as (C) except in islets from donors diagnosed with type 2 diabetes (n=17). **(E)** Shows the direct comparisons of time-to-reach peak between non-diabetic donors (n=123) and donors with type-2 diabetes (n=17) for each macronutrient tested. * indicates $p<0.05$, ** indicates $p<0.01$, *** indicates $p<0.001$, and **** indicates $p<0.0001$.

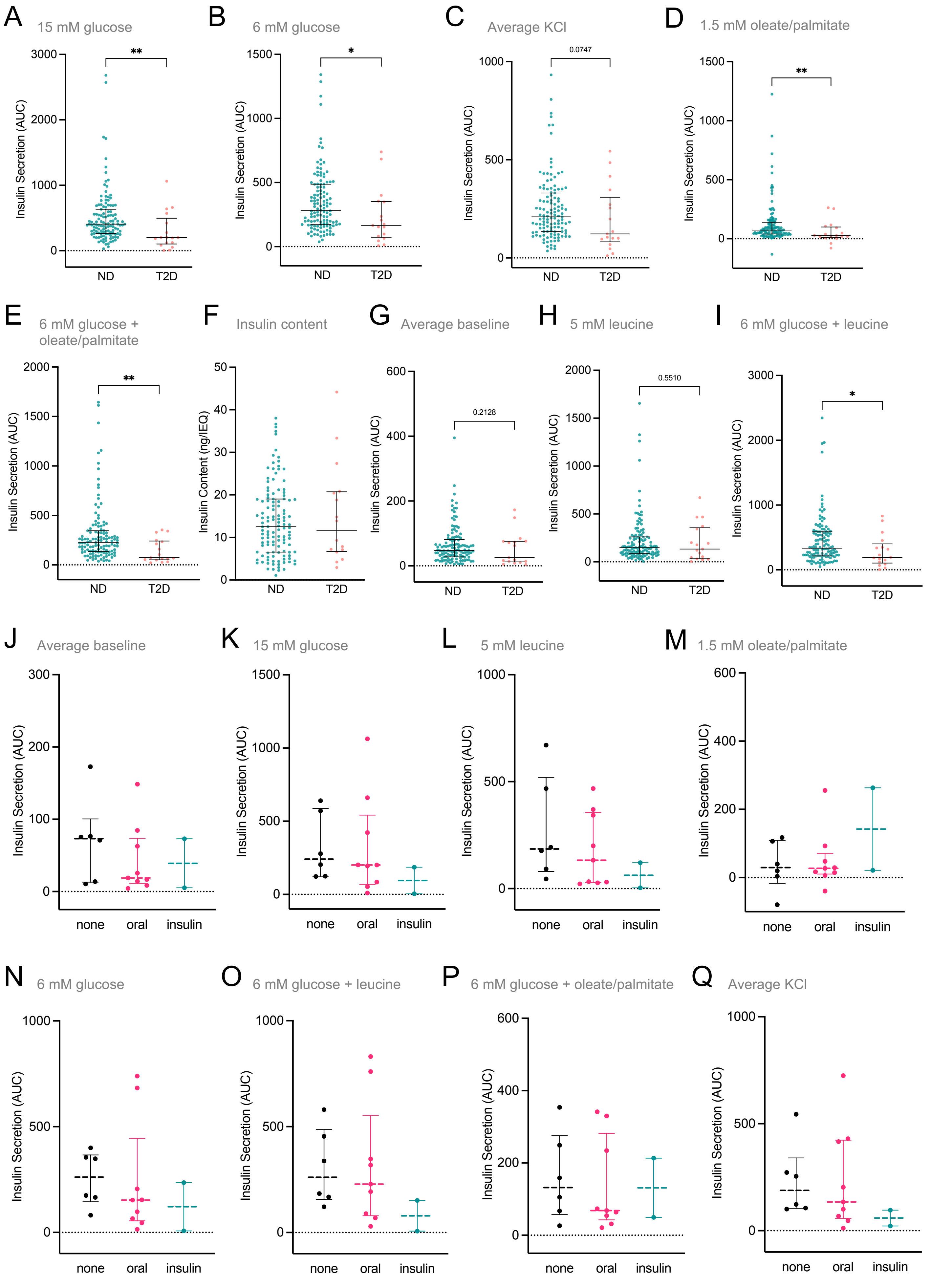
A 15 mM glucose
B 6 mM glucose
C Average KCl
D 1.5 mM oleate/palmitate
E 6 mM glucose + oleate/palmitate
F Insulin content
G Average baseline
H 5 mM leucine
I 6 mM glucose + leucine
J Average baseline
K 15 mM glucose
L 5 mM leucine
M 1.5 mM oleate/palmitate
N 6 mM glucose
O 6 mM glucose + leucine
P 6 mM glucose + oleate/palmitate
Q Average KCl
Insulin Secretion (AUC)
Insulin Content (ng/IEQ)
ND
T2D
none
oral
insulin
0.0747
0.2128
0.5510

Figure S2

Fig. S2. Related to Figure 1: Comparing nutrient-stimulated insulin secretion in ND donors and donors with T2D. (A-I) Direct comparisons of AUC values, or islet insulin content (ng/IEQ) between ND donors and donors with T2D. **(J-Q)** Shows direct comparisons of AUC values of donors with T2D grouped by known medication status. “None” indicates that the donors were not taking any known glucose-lowering medications. “Oral” indicates that donors were taking oral hypoglycemic mediations (metformin in most cases). “Insulin” indicates that the donors had progressed to needing exogenous insulin for glucose control. Median and interquartile range are shown. * indicates $p<0.05$, and ** indicates $p<0.01$.

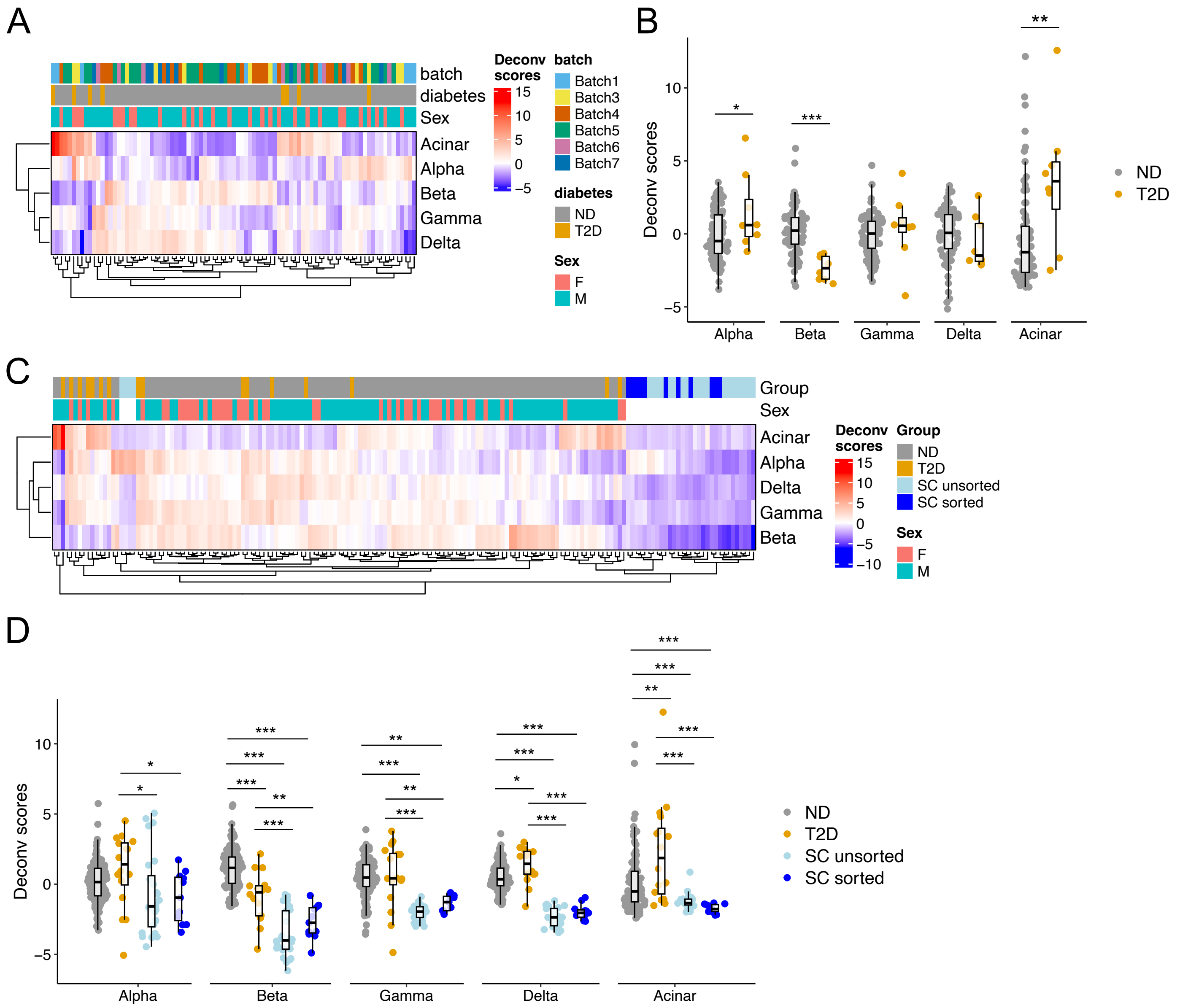

Fig. S3. Related to Figure 1 and Figure 5: Islet cell type deconvolution scores of human islets and stem cell-derived islet clusters from RNAseq or proteomics. (A) Heatmap showing the deconvolution scores from human islet RNAseq. Batches, diabetes status and sex are annotated on top. **(B)** Deconvolution scores from human islet RNAseq comparing ND and T2D donors. **(C)** Heatmap showing the deconvolution scores from human islet or stem cell (SC)-derived islet-like cluster proteomics. **(D)** Deconvolution scores from proteomics comparing ND and T2D human islets and unsorted or sorted stem cell (SC)-derived islet-clusters. * indicates $p<0.05$, ** indicates $p<0.01$, and *** indicates $p<0.001$.

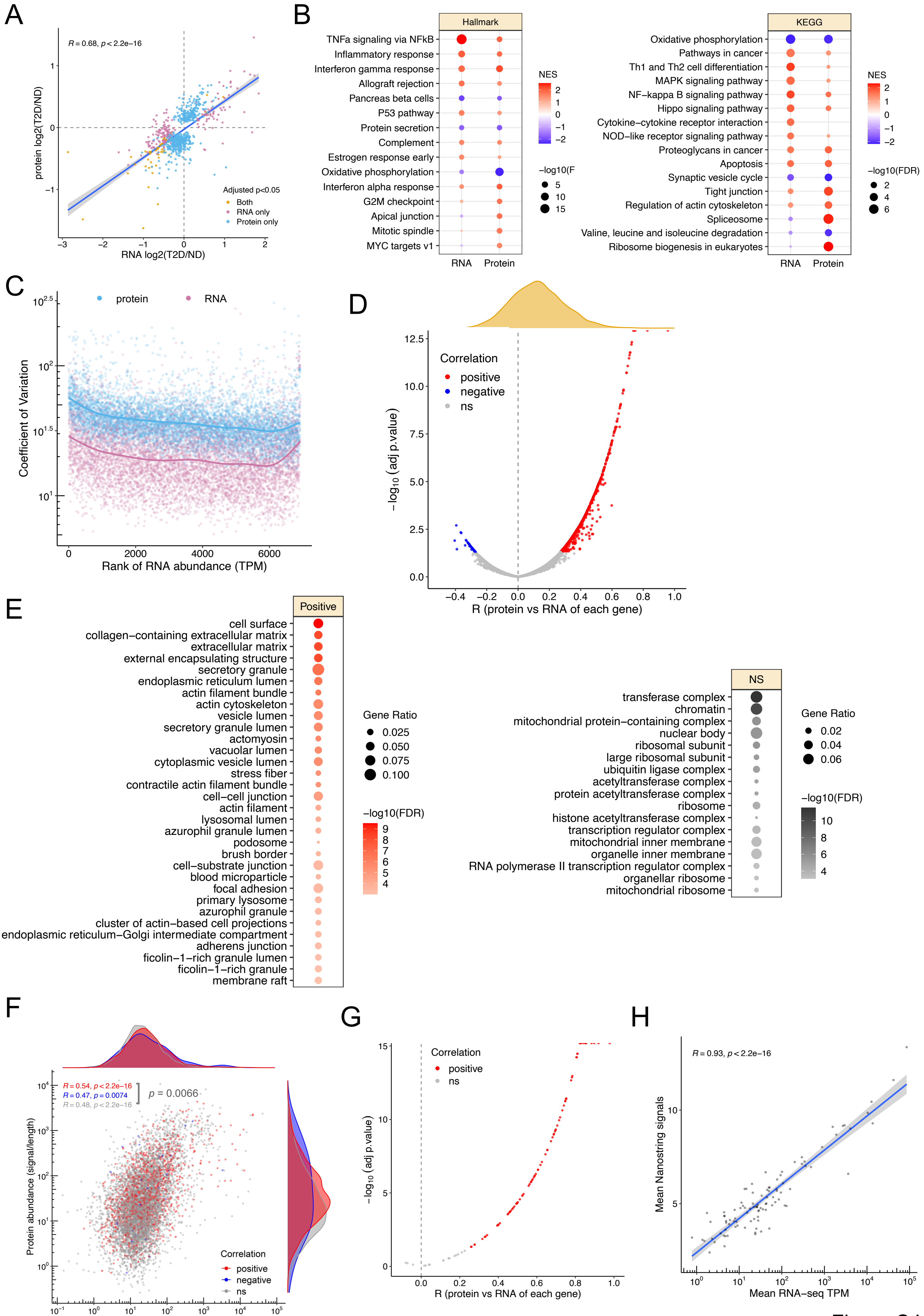

A
R = 0.68, p < 2.2e−16
protein log2(T2D/ND)
RNA log2(T2D/ND)
Adjusted p<0.05
Both
RNA only
Protein only
B
Hallmark
TNFa signaling via NFkB
Inflammatory response
Interferon gamma response
Allograft rejection
Pancreas beta cells
P53 pathway
Protein secretion
Complement
Estrogen response early
Oxidative phosphorylation
Interferon alpha response
G2M checkpoint
Apical junction
Mitotic spindle
MYC targets v1
RNA
Protein
NES
KEGG
Oxidative phosphorylation
Pathways in cancer
Th1 and Th2 cell differentiation
MAPK signaling pathway
NF−kappa B signaling pathway
Hippo signaling pathway
Cytokine−cytokine receptor interaction
NOD−like receptor signaling pathway
Proteoglycans in cancer
Apoptosis
Synaptic vesicle cycle
Tight junction
Regulation of actin cytoskeleton
Spliceosome
Valine, leucine and isoleucine degradation
Ribosome biogenesis in eukaryotes
−log10(FDR)
C
protein
RNA
Coefficient of Variation
Rank of RNA abundance (TPM)
D
Correlation
positive
negative
ns
−log10 (adj p.value)
R (protein vs RNA of each gene)
E
Positive
cell surface
collagen−containing extracellular matrix
extracellular matrix
external encapsulating structure
secretory granule
endoplasmic reticulum lumen
actin filament bundle
actin cytoskeleton
vesicle lumen
secretory granule lumen
actomyosin
vacuolar lumen
cytoplasmic vesicle lumen
stress fiber
contractile actin filament bundle
cell−cell junction
actin filament
lysosomal lumen
azurophil granule lumen
podosome
brush border
cell−substrate junction
blood microparticle
focal adhesion
primary lysosome
azurophil granule
cluster of actin−based cell projections
endoplasmic reticulum−Golgi intermediate compartment
adherens junction
ficolin−1−rich granule lumen
ficolin−1−rich granule
membrane raft
Gene Ratio
−log10(FDR)
NS
transferase complex
chromatin
mitochondrial protein−containing complex
nuclear body
ribosomal subunit
large ribosomal subunit
ubiquitin ligase complex
acetyltransferase complex
protein acetyltransferase complex
ribosome
histone acetyltransferase complex
transcription regulator complex
mitochondrial inner membrane
organelle inner membrane
RNA polymerase II transcription regulator complex
organellar ribosome
mitochondrial ribosome
F
R = 0.54, p < 2.2e−16
R = 0.47, p = 0.0074
R = 0.48, p < 2.2e−16
p = 0.0066
Protein abundance (signal/length)
RNA abundance (mean TPM)
Correlation
positive
negative
ns
G
Correlation
positive
ns
−log10 (adj p.value)
R (protein vs RNA of each gene)
H
R = 0.93, p < 2.2e−16
Mean Nanostring signals
Mean RNA−seq TPM

Figure S4

Fig. S4. Related to Figure 1: Comparative analyses of human islet transcriptomes and proteomes. (A) Correlation between the log2 fold changes of differentially expressed RNAs and proteins (T2D vs ND, adjusted $p < 0.05$). **(B)** Hallmark and selected KEGG gene sets enriched from the transcriptomic or proteomic changes in T2D (T2D vs ND, FDR<0.005 from transcriptome or proteome). **(C)** Variance (Coefficient of Variation) of RNA and protein of each gene (ranked by the RNA abundance). **(D)** Within gene, across sample RNA-protein correlations among the 90 donor samples (82 ND and 8 T2D) and 7609 genes that were commonly measured in RNA-seq and proteomics. 1422 (19%) or 37 (0.5%) genes are positive or negatively correlated, respectively. **(E)** GO - Cellular Component (GO_CC) functional enrichment analysis of the gene sets enriched by the positive (left) or non-significant (right) RNA-protein correlations. (FDR<0.0001). **(F)** Across-gene correlation between RNAs and proteins among the genes that have positive, negative, or non-significant (ns) within-gene correlations in (D). Statistical difference between their correlation coefficient (R) were calculated using the cocor R package **(G)** Within gene, across sample correlation between RNAs measured by RNA-seq and NanoString. 82 out of the 99 commonly detected genes (83%) are positively correlated among the 64 common donor samples. **(H)** Across-gene correlation between RNAs measured by RNA-seq and NanoString.

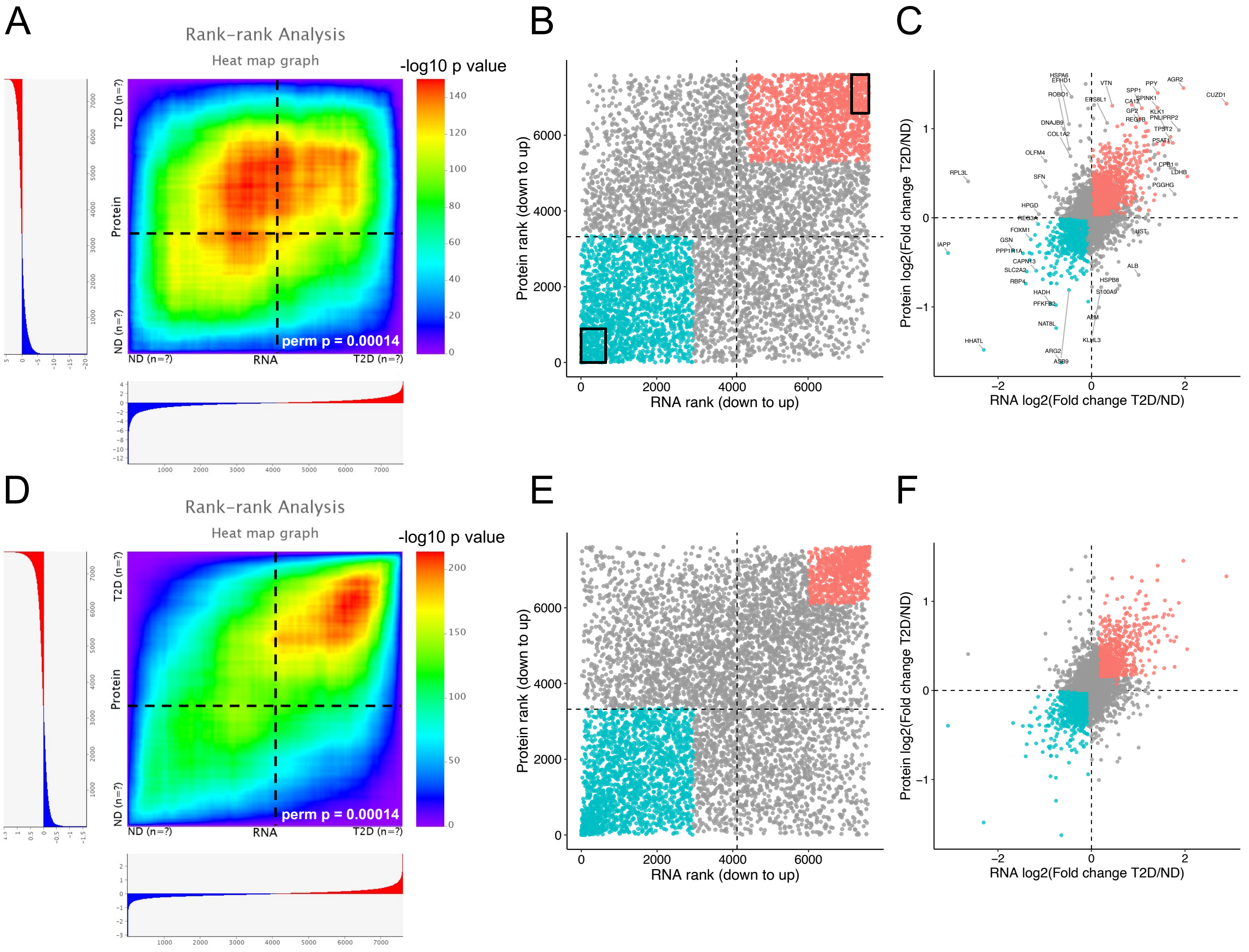

Figure S5. Related to Figure 1: Rank-rank hypergeometric overlap (RRHO) test comparing proteomic and transcriptomic changes in T2D. Proteins and RNAs commonly detected in our proteomics and RNA-seq datasets were ranked by –log10 p value **(A-C)** or by log2 fold change **(D-F)** in our differential expression analysis (T2D/ND). **(A)** Hypergeometric overlap map showing the hypergeometric p values (adjusted by Benjamini-Yekutieli FDR) at each rank threshold. **(B)** Rank-Rank scatter plot with the most statistically significant overlapping gene set colored in pink (~1500 up-up) or blue (~1800 down-down). Proteins and RNAs within the black square were altered in T2D (using a less stringent cut off - unadjusted p value < 0.05 in differential expression analysis), which are only a small subset of the overlapping gene set determined by RRHO. **(C)** The log2 fold changes of proteins and RNAs with the most statistically significant overlapping gene set colored in pink (up-up) or blue (down-down). **(D-F)** Same as (A-C) but using log2 fold changes to rank the proteins and RNAs in RRHO test. In E there are are ~800 up-up and ~1800 down-down overlapping gene sets.

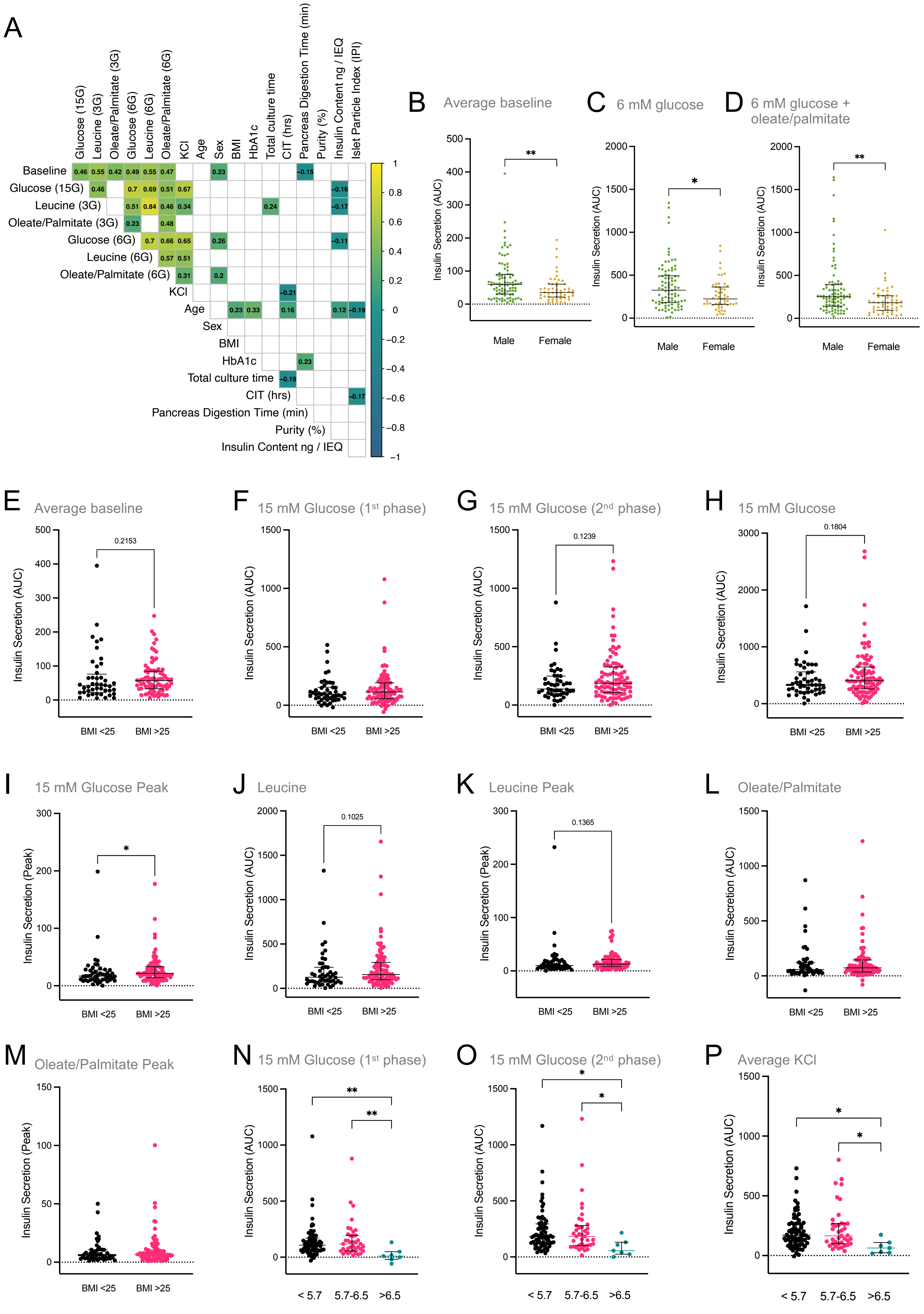
A
Glucose (15G)
Leucine (3G)
Oleate/Palmitate (3G)
Glucose (6G)
Leucine (6G)
Oleate/Palmitate (6G)
KCl
Age
Sex
BMI
HbA1c
Total culture time
CIT (hrs)
Pancreas Digestion Time (min)
Purity (%)
Insulin Content ng / IEQ
Islet Particle Index (IPI)
Baseline
B
Average baseline
Insulin Secretion (AUC)
Male
Female
C
6 mM glucose
D
6 mM glucose + oleate/palmitate
E
Average baseline
0.2153
BMI <25
BMI >25
F
15 mM Glucose (1st phase)
G
15 mM Glucose (2nd phase)
0.1239
H
15 mM Glucose
0.1804
I
15 mM Glucose Peak
Insulin Secretion (Peak)
J
Leucine
0.1025
K
Leucine Peak
0.1365
L
Oleate/Palmitate
M
Oleate/Palmitate Peak
N
15 mM Glucose (1st phase)
< 5.7
5.7-6.5
>6.5
O
15 mM Glucose (2nd phase)
P
Average KCl
Figure S6

Fig. S6. Related to Figure 1 and Figure 2: Donor metrics and islet function. (A) Multiple comparisons matrix showing correlations between indicated parameters. Only significant R-values are shown. **(B-D)** Shows direct comparisons of AUC values of indicated areas of the perifusion curve. between all donors separated by biological sex (male (n=89) and female (n=51)). Only measurements that show significant differences between the male and female donors are shown. **(E-M)** Shows direct comparisons of selected AUC values or peak values of indicated areas of the perifusion curve between all donors separated by BMI. **(N-P)** Shows direct comparisons of 1st and 2nd phase 15 mM glucose-stimulated insulin secretion as well as KCl-stimulated insulin secretion between all donors stratified by HbA1c. * indicates $p<0.05$, ** indicates $p<0.01$.

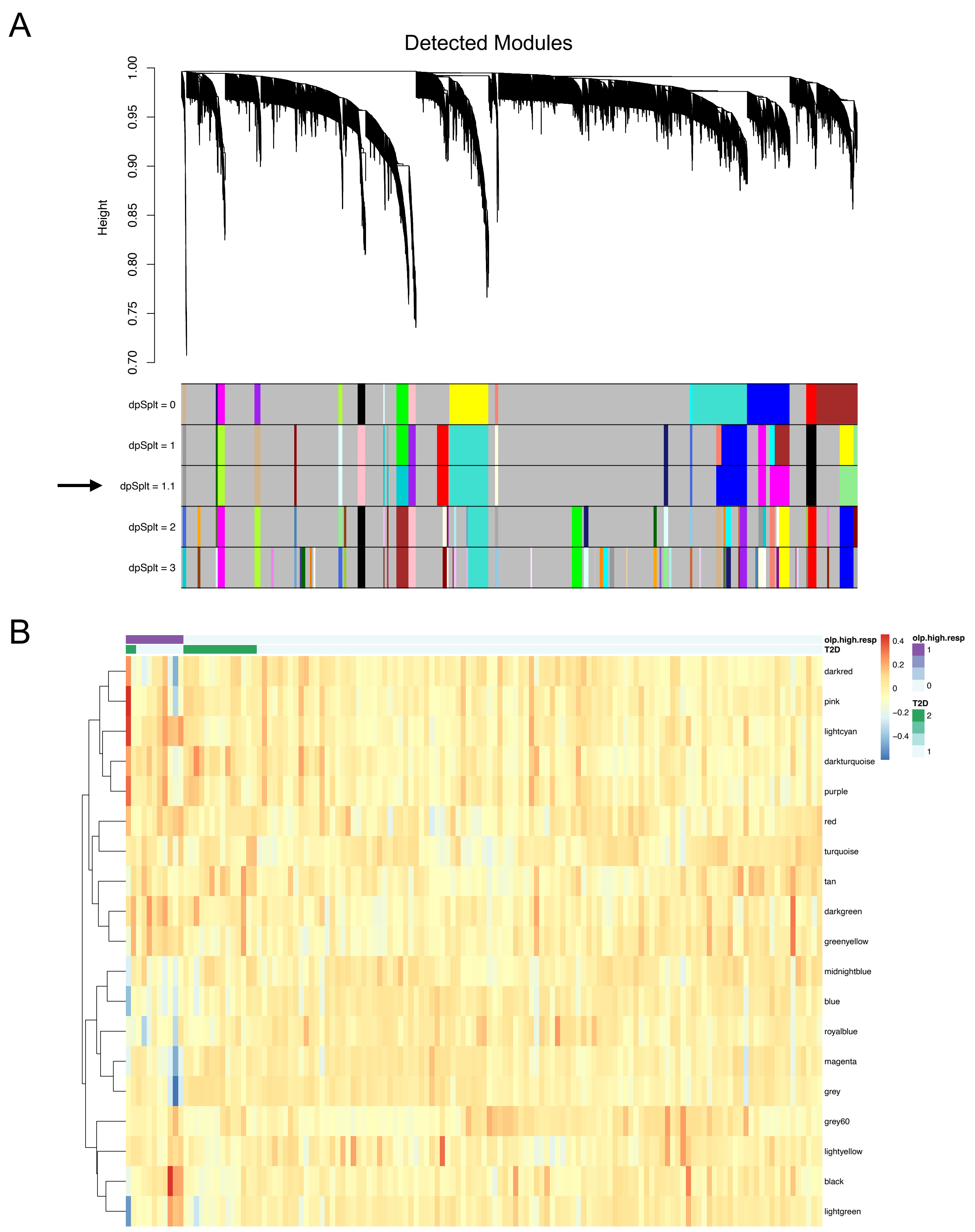
A
Detected Modules
Height
1.00
0.95
0.90
0.85
0.80
0.75
0.70
dpSplt = 0
dpSplt = 1
dpSplt = 1.1
dpSplt = 2
dpSplt = 3
B
olp.high.resp
T2D
darkred
pink
lightcyan
darkturquoise
purple
red
turquoise
tan
darkgreen
greenyellow
midnightblue
blue
royalblue
magenta
grey
grey60
lightyellow
black
lightgreen
0.4
0.2
0
−0.2
−0.4
olp.high.resp
1
0
T2D
2
1

Figure S7

Fig. S7. Related to Figure 2: Detailed proteomics co-expression network results. (A) Diagram showing hierarchically clustered proteins and their module membership. The height of the diagram corresponds to the dissimilarity of the two merged proteins for each node in the dendrogram. The bands below the dendrogram show detected modules for 4 different values of the 'deepSplit' parameter (dpSplit = 0, 1, 2, 3), as well as modules merged based on the correlation of their eigengenes for deepSplit parameter = 1 (dpSplit = 1.1; these module definitions were used for all downstream analyses). **(B)** Heatmap of module eigengenes for all donors. Donors are annotated with whether they were oleate palmitate high responders (purple = yes, light blue = no) and whether they were diagnosed with T2D (green = yes, light blue = no). Modules (rows) are hierarchically clustered while donors (columns) are sorted first according to whether they are oleate palmitate high responders, and second according to whether they were reported to have T2D.

A All modules

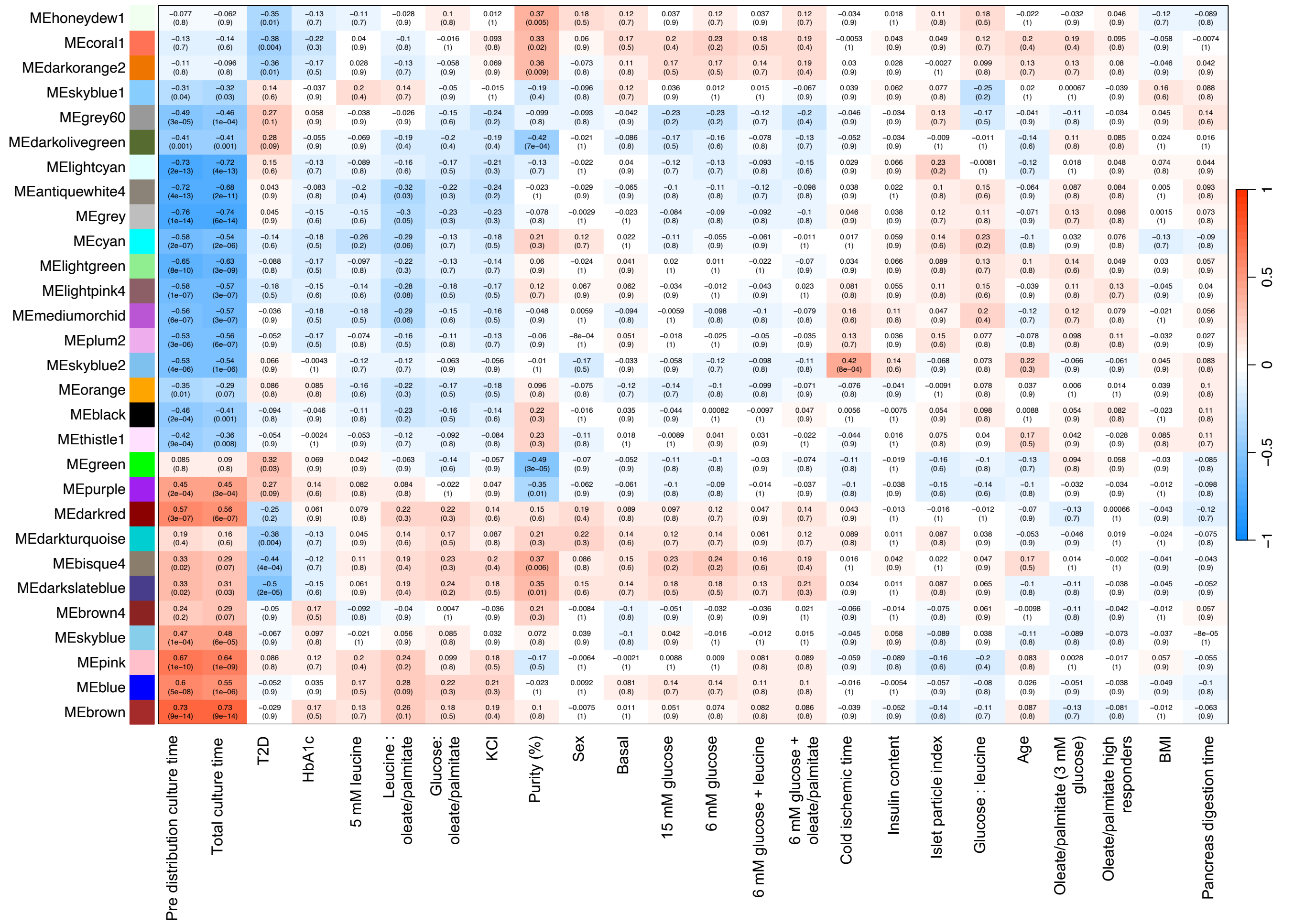

B Modules adjusted for isolation parameters

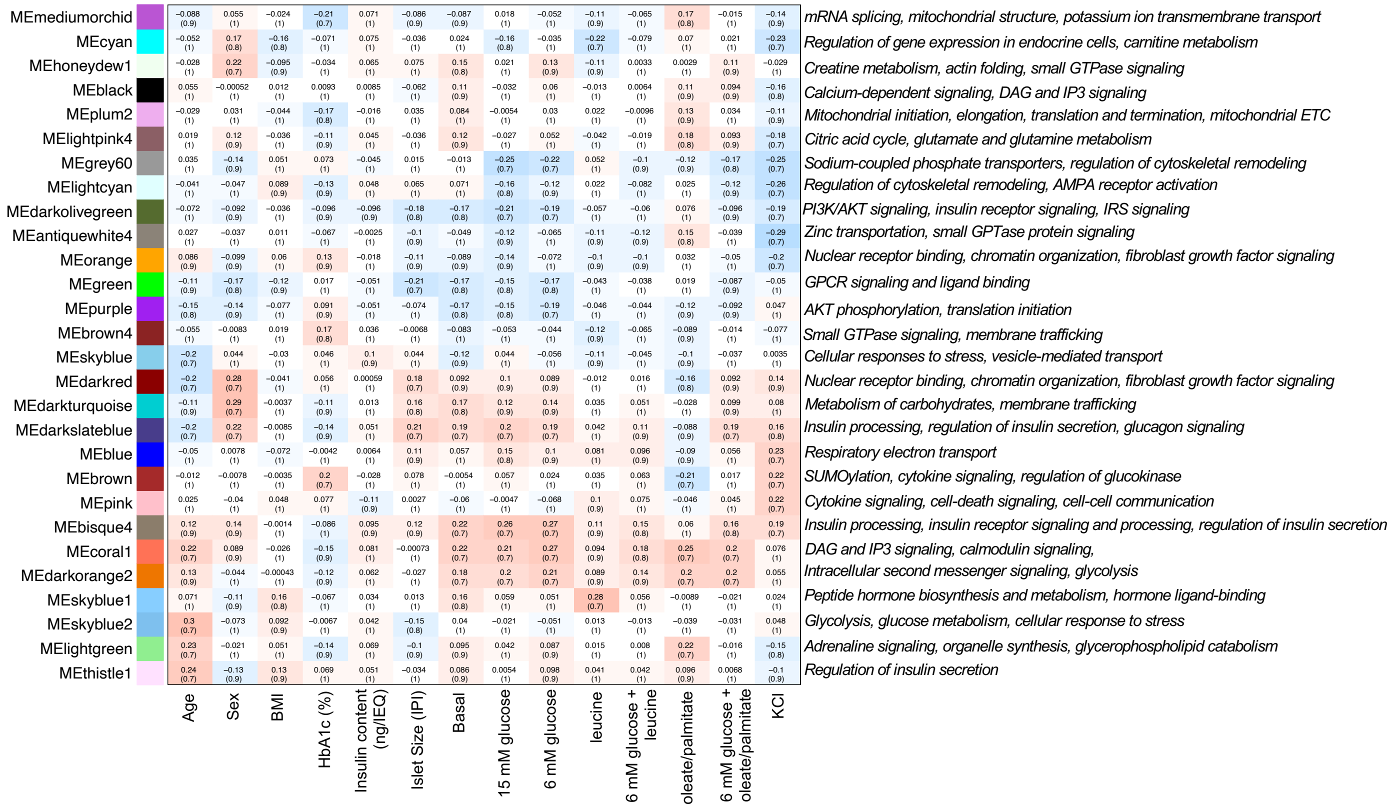

Figure S8

Fig. S8. Related to Figure 2: Association between RNAseq modules and donor metadata. Heatmap of module - metadata associations, with pairwise correlations computed as **(A)** the Pearson correlation between module eigengenes and metadata values, and **(B)** the partial Pearson correlation between module eigengenes and metadata values other than technical isolation parameters (culture time, digestion time, cold ischemia time, and percent purity), with these parameters included as covariates in the partial correlation calculations. Cell color corresponds to correlation value, and each cell is annotated with the correlation value and FDR-adjusted p-value (FDR = 0.05) in brackets. Heatmap row (modules) and columns (metadata values) are hierarchically clustered based on correlation magnitude. Modules in B) are additionally labeled with representative functional themes from overrepresentation gene set analysis with the KEGG, Reactome, Gene Ontology Cellular Component, Molecular Function, and Biological Process functional libraries.

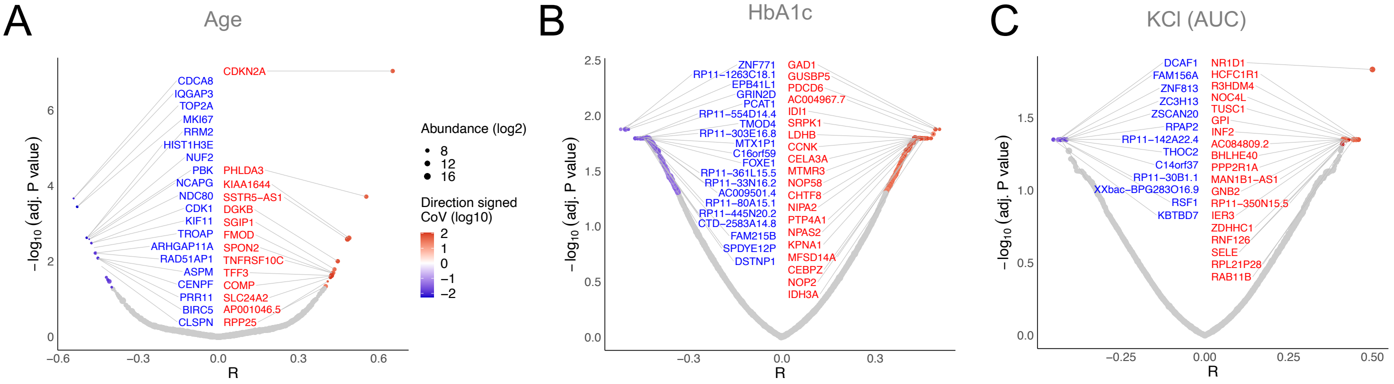

Fig. S9. Related to Figure 3: RNAseq versus donor metadata and islet function. Volcano plots for mRNAs significantly negatively (blue) or positively (red) correlated to **(A)** donor age, **(B)** donor HbA1c, and **(C)** insulin secretion stimulated by direct depolarization with KCl (30 mM). Protein abundances (log2) are depicted by size of circle, and the coefficient of variance (log10) is depicted by color gradient. Volcano plots are only shown if significant correlations were found.

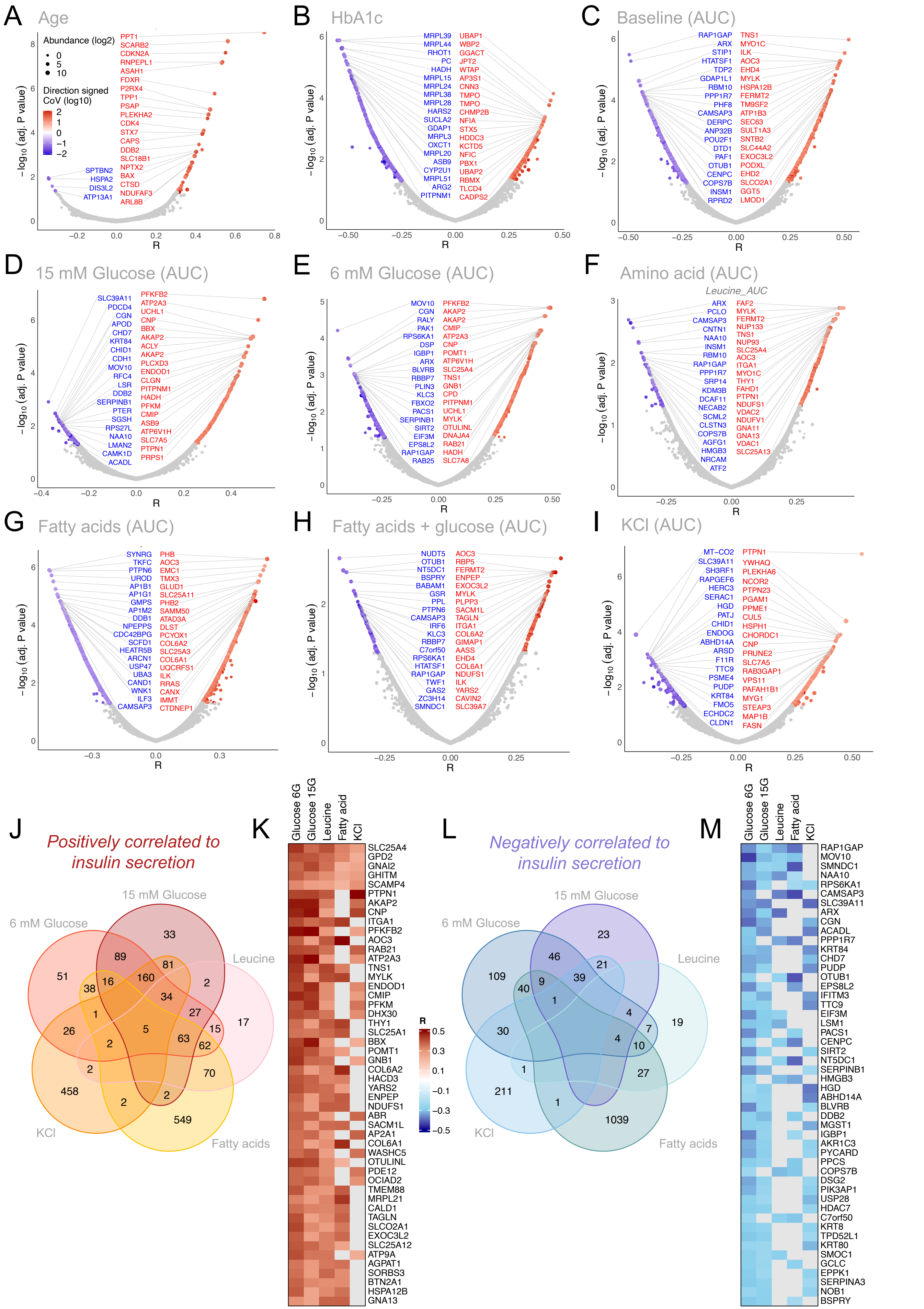

A Age
B HbA1c
C Baseline (AUC)
D 15 mM Glucose (AUC)
E 6 mM Glucose (AUC)
F Amino acid (AUC)
Leucine_AUC
G Fatty acids (AUC)
H Fatty acids + glucose (AUC)
I KCl (AUC)
J Positively correlated to insulin secretion
K
L Negatively correlated to insulin secretion
M
6 mM Glucose
15 mM Glucose
Leucine
KCl
Fatty acids
Glucose 6G
Glucose 15G
Leucine
Fatty acid
KCl

Figure S10

Fig. S10. Related to Figure 3: Correlations of individual proteins and mRNAs with insulin secretory responses. Volcano plots are shown depicting significant positive (red) and negative (blue) correlations of proteins to continuous donor characteristics: **(A)** donor age and **(B)** HbA1c; or functional parameters in response to: **(C)** 3 mM glucose **(D)** 15 mM glucose **(E)** 6 mM glucose **(F)** 5 mM leucine **(G)** 1.5 mM oleate/palmitate (1:1 mix) **(H)** 1.5 mM oleate/palmitate + 6 mM glucose and **(I)** 30 mM KCl. Protein abundances (log2) are depicted by size of circle, and the coefficient of variance (log10) is depicted by color gradient. **(J)** Venn diagram showing the overlap of the number of protein abundances that positively correlate to the indicated nutrient stimuli. **(K)** Heat map depicts the top 50 most positively correlative proteins to the indicated stimuli. **(L-M)** Shows the same as (J-K), but for negative correlations.

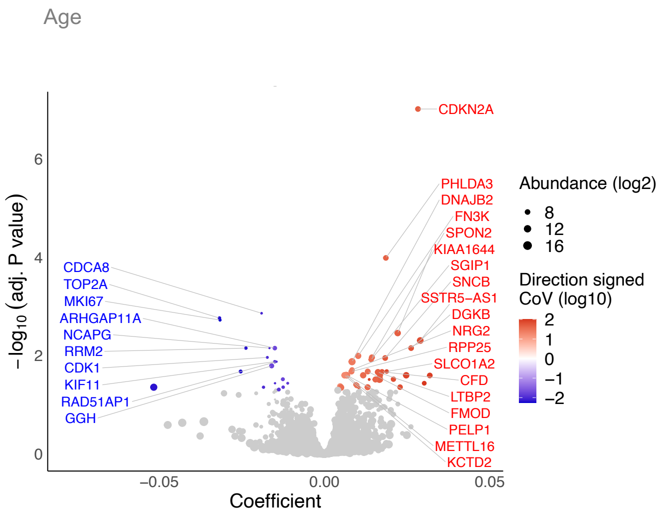

Fig. S11. Related to Figure 3: Association between RNAseq donor metadata and islet function. Volcano plot depicting significant positive (red) and negative (blue) linear regression coefficients (adjusting for T2D status) of transcripts associated with donor age. RNA expression (abundances) are depicted by size of circle (log2), and the coefficient of variation (log10) is depicted by color gradient. Volcano plots are only shown if significant associations were found.

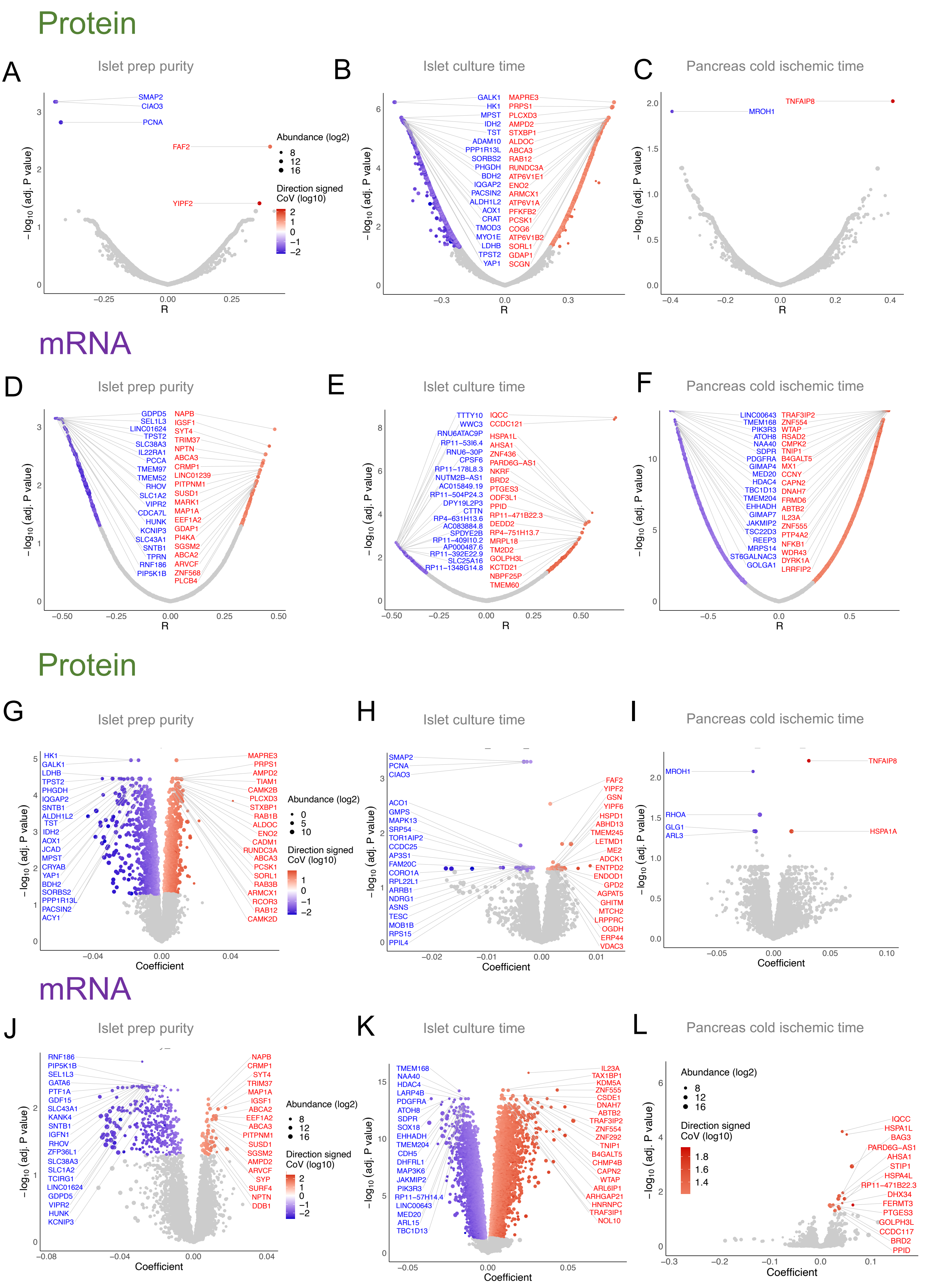

Protein
A
Islet prep purity
SMAP2
CIAO3
PCNA
FAF2
YIPF2
Abundance (log2)
8
12
16
Direction signed CoV (log10)
2
1
0
−1
−2
−log10 (adj. P value)
R
B
Islet culture time
GALK1
HK1
MPST
IDH2
TST
ADAM10
PPP1R13L
SORBS2
PHGDH
BDH2
IQGAP2
PACSIN2
ALDH1L2
AOX1
CRAT
TMOD3
MYO1E
LDHB
TPST2
YAP1
MAPRE3
PRPS1
PLCXD3
AMPD2
STXBP1
ALDOC
ABCA3
RAB12
RUNDC3A
ATP6V1E1
ENO2
ARMCX1
ATP6V1A
PFKFB2
PCSK1
COG6
ATP6V1B2
SORL1
GDAP1
SCGN
C
Pancreas cold ischemic time
TNFAIP8
MROH1
mRNA
D
Islet prep purity
GDPD5
SEL1L3
LINC01624
TPST2
SLC38A3
IL22RA1
PCCA
TMEM97
TMEM52
RHOV
SLC1A2
VIPR2
CDCA7L
HUNK
KCNIP3
SLC43A1
SNTB1
TPRN
RNF186
PIP5K1B
NAPB
IGSF1
SYT4
TRIM37
NPTN
ABCA3
CRMP1
LINC01239
PITPNM1
SUSD1
MARK1
MAP1A
EEF1A2
GDAP1
PI4KA
SGSM2
ABCA2
ARVCF
ZNF568
PLCB4
E
Islet culture time
TTTY10
WWC3
RNU6ATAC9P
RP11−5316.4
RNU6−30P
CPSF6
RP11−178L8.3
NUTM2B−AS1
AC015849.19
RP11−504P24.3
DPY19L2P3
CTTN
RP4−631H13.6
AC083884.8
SPDYE2B
RP11−409I10.2
AP000487.6
RP11−392E22.9
SLC25A16
RP11−1348G14.8
IQCC
CCDC121
HSPA1L
AHSA1
ZNF436
PARD6G−AS1
NKRF
BRD2
PTGES3
ODF3L1
PPID
RP11−471B22.3
DEDD2
RP4−751H13.7
MRPL18
TM2D2
GOLPH3L
KCTD21
NBPF25P
TMEM60
F
Pancreas cold ischemic time
LINC00643
TMEM168
PIK3R3
ATOH8
NAA40
SDPR
PDGFRA
GIMAP4
MED20
HDAC4
TBC1D13
TMEM204
EHHADH
GIMAP7
JAKMIP2
TSC22D3
REEP3
MRPS14
ST6GALNAC3
GOLGA1
TRAF3IP2
ZNF554
WTAP
RSAD2
CMPK2
TNIP1
B4GALT5
MX1
CCNY
CAPN2
DNAH7
FRMD6
ABTB2
IL23A
ZNF555
PTP4A2
NFKB1
WDR43
DYRK1A
LRRFIP2
Protein
G
Islet prep purity
HK1
GALK1
LDHB
TPST2
PHGDH
IQGAP2
SNTB1
ALDH1L2
TST
IDH2
AOX1
JCAD
MPST
CRYAB
YAP1
BDH2
SORBS2
PPP1R13L
PACSIN2
ACY1
MAPRE3
PRPS1
AMPD2
TIAM1
CAMK2B
PLCXD3
STXBP1
RAB1B
ALDOC
ENO2
CADM1
RUNDC3A
ABCA3
PCSK1
SORL1
RAB3B
ARMCX1
RCOR3
RAB12
CAMK2D
Abundance (log2)
0
5
10
Direction signed CoV (log10)
1
0
−1
−2
Coefficient
H
Islet culture time
SMAP2
PCNA
CIAO3
ACO1
GMPS
MAPK13
SRP54
TOR1AIP2
CCDC25
AP3S1
FAM20C
CORO1A
RPL22L1
ARRB1
NDRG1
ASNS
TESC
MOB1B
RPS15
PPIL4
FAF2
YIPF2
GSN
YIPF6
HSPD1
ABHD13
TMEM245
LETMD1
ME2
ADCK1
ENTPD2
ENDOD1
GPD2
AGPAT5
GHITM
MTCH2
LRPPRC
OGDH
ERP44
VDAC3
I
Pancreas cold ischemic time
MROH1
TNFAIP8
RHOA
GLG1
ARL3
HSPA1A
mRNA
J
Islet prep purity
RNF186
PIP5K1B
SEL1L3
GATA6
PTF1A
GDF15
SLC43A1
KANK4
SNTB1
IGFN1
RHOV
ZFP36L1
SLC38A3
SLC1A2
TCIRG1
LINC01624
GDPD5
VIPR2
HUNK
KCNIP3
NAPB
CRMP1
SYT4
TRIM37
MAP1A
IGSF1
ABCA2
EEF1A2
ABCA3
PITPNM1
SUSD1
SGSM2
AMPD2
ARVCF
SYP
SURF4
NPTN
DDB1
Abundance (log2)
8
12
16
Direction signed CoV (log10)
2
1
0
−1
−2
K
Islet culture time
TMEM168
NAA40
HDAC4
LARP4B
PDGFRA
ATOH8
SDPR
SOX18
EHHADH
TMEM204
CDH5
DHFRL1
MAP3K6
JAKMIP2
PIK3R3
RP11−57H14.4
LINC00643
MED20
ARL15
TBC1D13
IL23A
TAX1BP1
KDM5A
ZNF555
CSDE1
DNAH7
ABTB2
TRAF3IP2
ZNF554
ZNF292
TNIP1
B4GALT5
CHMP4B
CAPN2
WTAP
ARL6IP1
ARHGAP21
HNRNPC
TRAF3IP1
NOL10
L
Pancreas cold ischemic time
Abundance (log2)
8
12
16
Direction signed CoV (log10)
1.8
1.6
1.4
IQCC
HSPA1L
BAG3
PARD6G−AS1
AHSA1
STIP1
HSPA4L
RP11−471B22.3
DHX34
FERMT3
PTGES3
GOLPH3L
CCDC117
BRD2
PPID

Figure S12

Fig. S12. Related to Figure 3: Proteome and transcriptome versus islet isolation parameters. Volcano plots for proteins significantly negatively (blue) or positively (red) correlated to **(A)** islet preparation purity, **(B)** total islet culture time and **(C)** cold ischemic time of the pancreas. **(D-F)** Shows the same as (A-C) except for correlations with the transcriptome. Protein abundances (log2) are depicted by size of circle, and the coefficient of variance (log10) is depicted by color gradient. All donors (independent of diabetes diagnosis) were used in these analyses. **(G-I)** depicts volcano plots with significant positive (red) and negative (blue) linear regression coefficients (adjusting for T2D status) of proteins to islet preparation purity, total islet culture time and cold ischemic time of the pancreas. **(J-L)** Shows the same as (G-I) except for associations with the transcriptome. Protein and transcript abundances (log2) are depicted by size of circle, and the coefficient of variation (log10) is depicted by color gradient.

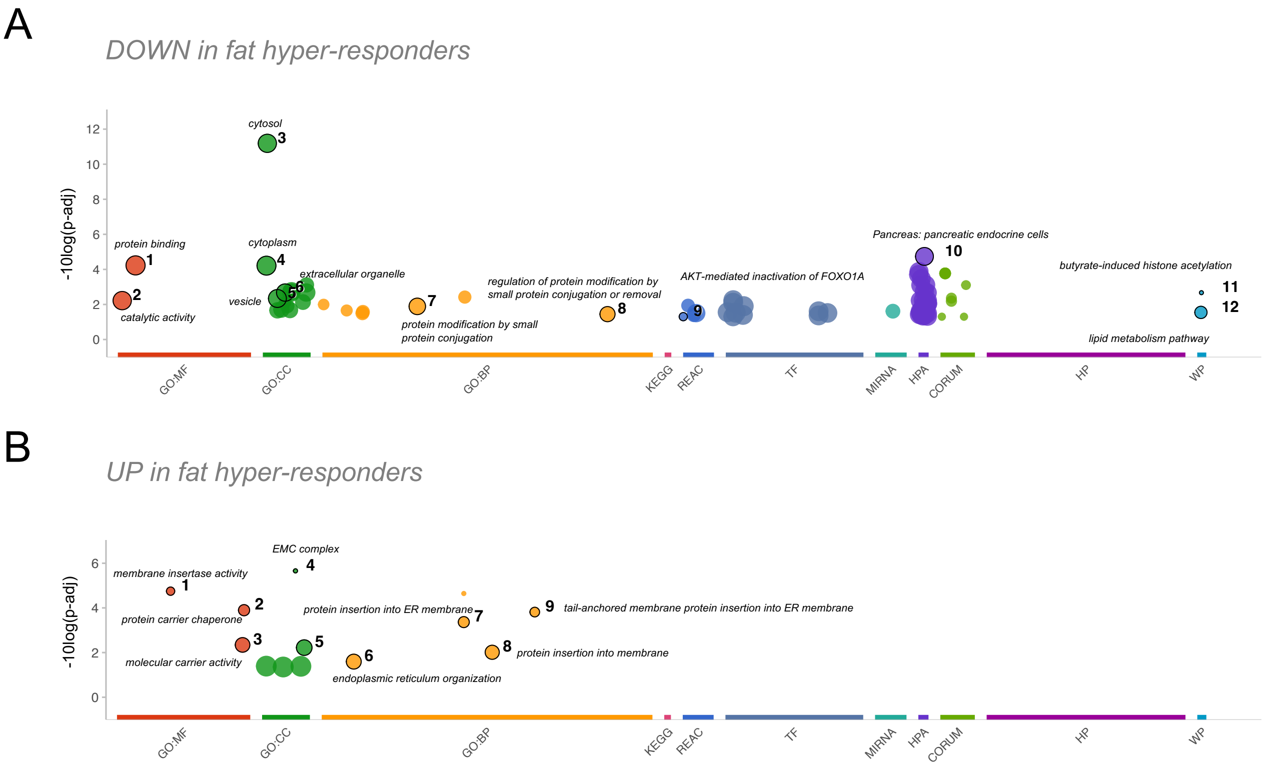

Fig. S13. Related to Figure 4: Pathway analysis of differentially abundant proteins in fat hyper-responders. (A) Pathway analysis of proteins that are less abundant in fat hyper-responsive donors showing enriched biological functions and pathways obtained from multiple databases including (among others), Gene Ontology (GO), KEGG and Reactome. **(B)** Shows the same as (A) except for proteins that are more abundant in fat hyper-responsive donors.

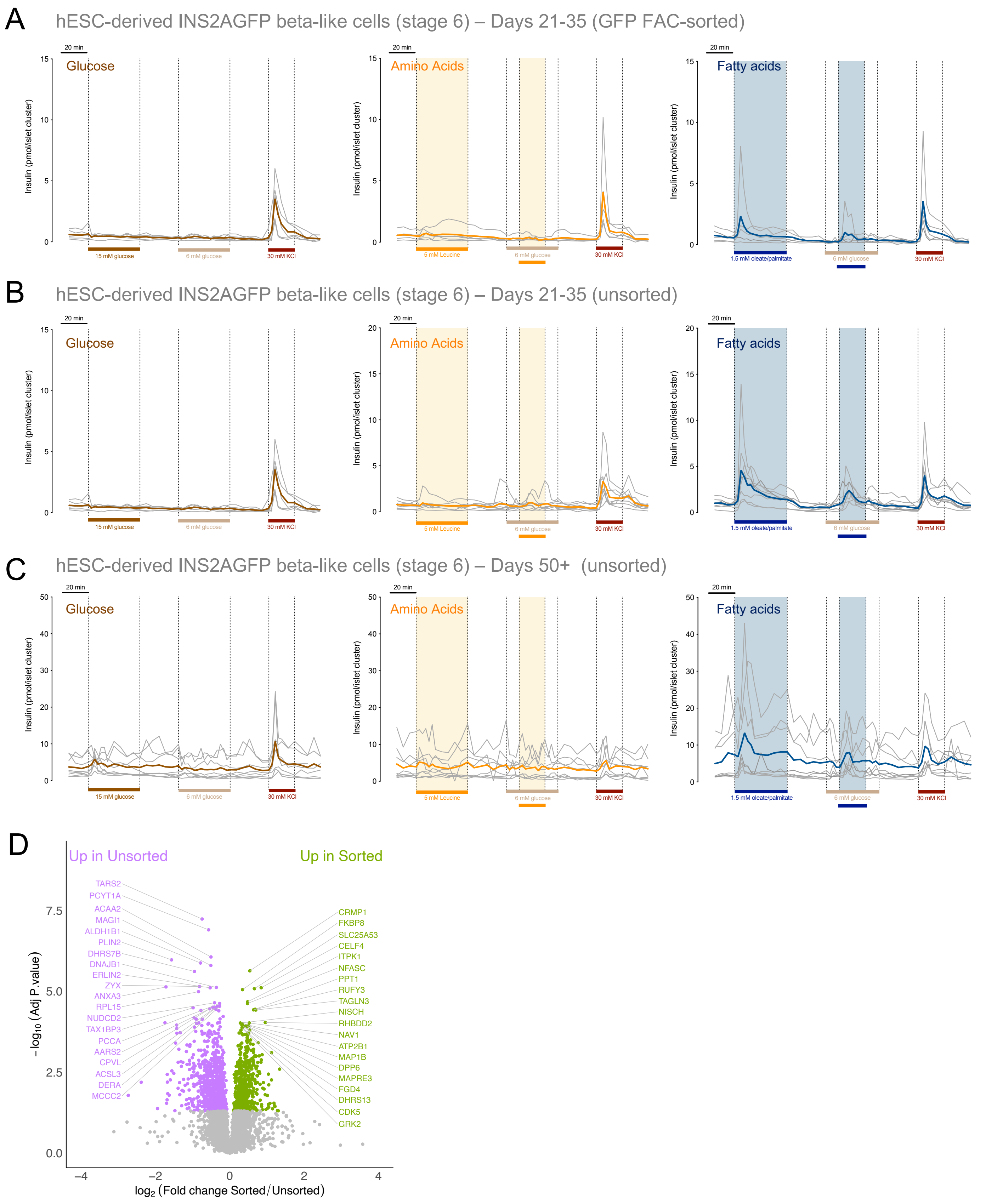
A
hESC-derived INS2AGFP beta-like cells (stage 6) – Days 21-35 (GFP FAC-sorted)
B
hESC-derived INS2AGFP beta-like cells (stage 6) – Days 21-35 (unsorted)
C
hESC-derived INS2AGFP beta-like cells (stage 6) – Days 50+ (unsorted)
20 min
Glucose
Amino Acids
Fatty acids
Insulin (pmol/islet cluster)
15 mM glucose
6 mM glucose
30 mM KCl
5 mM Leucine
1.5 mM oleate/palmitate
D
Up in Unsorted
Up in Sorted
TARS2
PCYT1A
ACAA2
MAGI1
ALDH1B1
PLIN2
DHRS7B
DNAJB1
ERLIN2
ZYX
ANXA3
RPL15
NUDCD2
TAX1BP3
PCCA
AARS2
CPVL
ACSL3
DERA
MCCC2
CRMP1
FKBP8
SLC25A53
CELF4
ITPK1
NFASC
PPT1
RUFY3
TAGLN3
NISCH
RHBDD2
NAV1
ATP2B1
MAP1B
DPP6
MAPRE3
FGD4
DHRS13
CDK5
GRK2
$-\log_{10}$(Adj P.value)
$\log_2$(Fold change Sorted/Unsorted)

Figure S14

Fig. S14. Related to Figure 5: Stem cell-derived islet individual response traces. (A) Individual (grey) and averaged traces (colored) of dynamic insulin secretion measurements in response to glucose (15 or 6 mM), leucine (5 mM), oleate and palmitate (1.5 mM, 1:1 mixture) or KCl (30 mM) in stem cell-derived islet (5 differentiations, GFP-Fluorescence-activated cell (FAC) sorted, stage 6 (days 21-25). Basal glucose was 3 mM. **(B)** Same as (A) except in 8 differentiations of unsorted clusters (stage 6, days 21-30). **(C)** Same as (B) except in 8 differentiations of older-maturing clusters (stage 6, days 55+). **(D)** Volcano plot showing differential protein abundance between GFP-FAC sorted stem cell derived islet like clusters (purple, n=11) and GFP-unsorted clusters (green, n=25). The top 30 most significant differentially abundant proteins are highlighted by labelling with gene name.

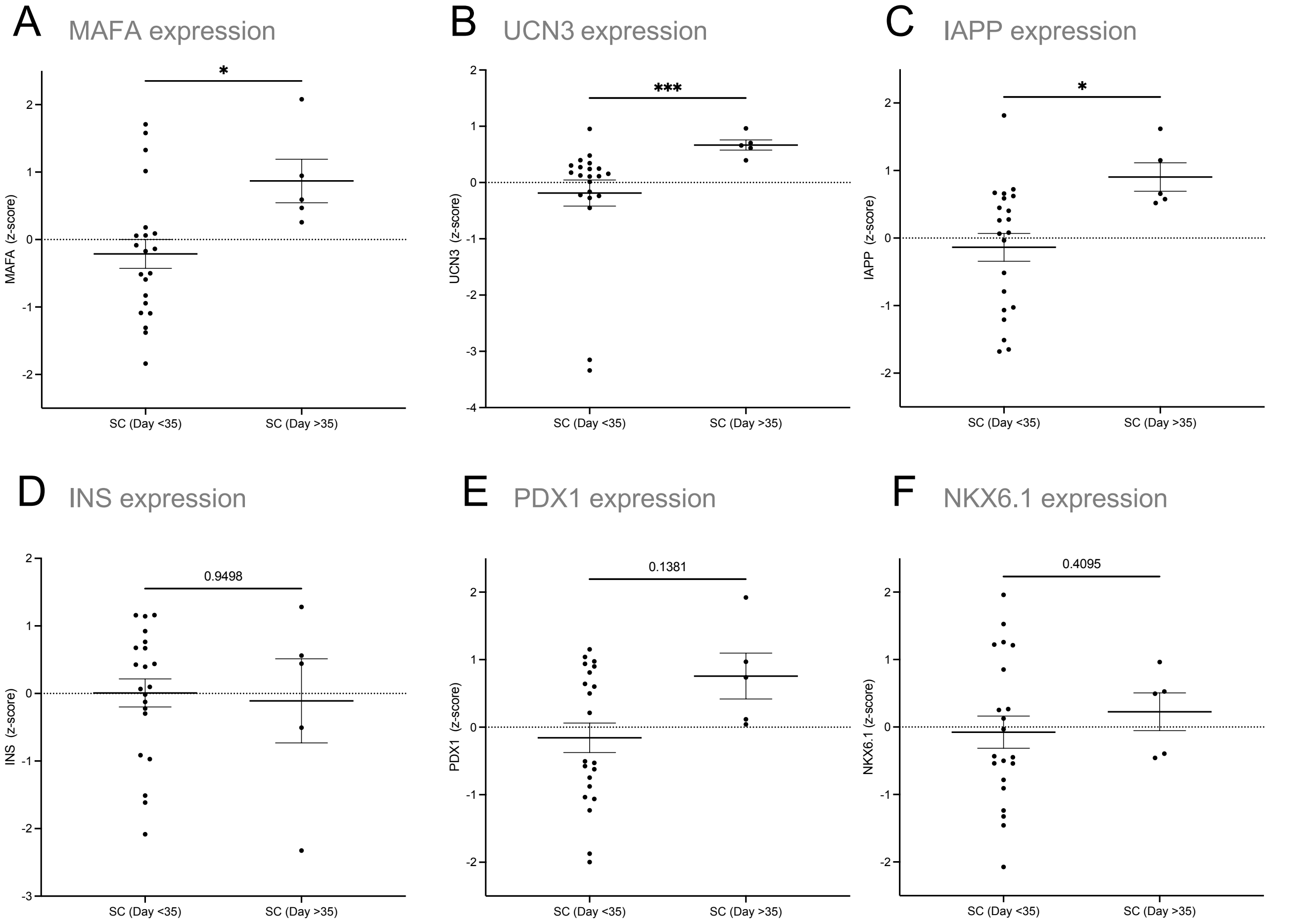

Fig. S15. Related to Figure 5: Transcriptional maturation of stem cell derived islets. Calculated z-scores of key maturation transcripts **(A)** MAFA, **(B)** UCN3, **(C)** IAPP, **(D)** INS, **(E)** PDX1, and **(F)** NKX6.1 are compared between stem cell-derived islet clusters less than and greater than 35 days of age. * indicates $p<0.05$, and *** indicates $p<0.001$.
